# Hypercarbon‐Centered Gold(I)‐Copper(I) Clusters Exhibiting a Dissolution/Crystallization‐Induced Photoluminochromism

**DOI:** 10.1002/chem.202600011

**Published:** 2026-02-24

**Authors:** Zhen Lei, Pei Zhao, Soichi Kikkawa, Xiao‐Li Pei, Wen‐Ting Liu, Hitoshi Ube, Seiji Yamazoe, Masahiro Ehara, Mitsuhiko Shionoya

**Affiliations:** ^1^ Department of Chemistry, Graduate School of Science The University of Tokyo Bunkyo‐ku Japan; ^2^ Research Center for Computational Science Institute For Molecular Science and SOKENDAI Okazaki Japan; ^3^ Department of Chemistry, Graduate School of Science Tokyo Metropolitan University Hachioji Japan; ^4^ Current address: Fujian Provincial Key Laboratory of Advanced Inorganic Oxygenated Materials College of Chemistry Fuzhou University Fuzhou P. R. China; ^5^ Current address: Research Institute For Science and Technology Tokyo University of Science Noda Japan

**Keywords:** chromism, copper, dissolution/crystallization, gold cluster, photoluminescence

## Abstract

Chemical molecules may show very different properties in the solid state and in solution, which is mainly caused by the difference in structure between different physical states. Well‐defined metal cluster molecules are ideal models for the investigation of the dynamic dissolution/crystallization process, as they usually exhibit photoluminescence (PL) that is sensitive to reversible changes in coordination geometry and multi‐electron structure. Here we report the dissolution/crystallization‐induced photoluminochromism (PLC) of carbon‐centered Au^I^_6_Cu^I^_2_ clusters bearing pyridylphosphine or pyridyl *N*‐heterocyclic carbene (NHC) ligands. These clusters, with an octahedral structure doubly capped with copper(I), exhibit greenish‐yellow emission in the solid state. Remarkably, the PL of the NHC‐protected clusters in solution changes only slightly, whereas the phosphine‐protected clusters exhibit red PL accompanied by a large bathochromic shift (>100 nm), a reversible process upon dissolution equilibration. X‐ray absorption spectroscopy and theoretical calculations suggest that the octahedral CAu^I^_6_ structure in the crystal is twisted into a triangular prismatic structure in solution. This remarkable ligand effect on the dissolution/crystallization‐induced PLC would provide advanced design guidance for stimuli‐responsive chromic materials.

## Graphical abstract

A novel photoluminochromism (PLC, > 100 nm shifts) of carbon‐centered gold cluster bearing pyridylphosphine ligands is reported. Upon dissolution/crystallization, the greenish‐yellow emissive solids and red emissive solution may reversibly convert into each other, and such processes are prohibited when utilizing pyridyl *N*‐heterocyclic carbene (NHC) as ligands. The origin of such chromism is a structure twist in the metal core of the cluster.

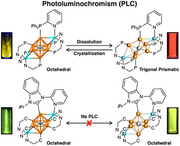

## 1 Introduction

Metal complexes often exhibit chromism in response to external stimuli and have attracted attention for their potential as functional materials involving bond cleavage, conformational and electronic structure changes, isomerization, and charge‐transfer interactions [1, 2, 3, 4, 5, 6, 7]. In addition to common external stimuli such as heat, light, pressure, solvent/vapor, and applied electric potential, the chromism occurring between the crystalline and solution states of a compound can be reversibly induced by the difference between the molecular arrangement in the crystal and the solvation structure in the solution. Therefore, the study of the structure and mechanism of such chromic molecules will provide new guidance for the design of chromophores. However, in many cases, geometric isomerization or slight conformational changes can have a significant effect on the electronic structure and therefore the physical properties of a molecule, making it difficult to understand the structure‐property relationship. In this regard, sub‐nanometer to nanometer sized metal clusters have attracted great attention as excellent multi‐electron molecular systems via dynamic metal‐metal and metal‐ligand interactions [8, 9]. For example, luminescent gold(I) clusters often exhibit mechanochromism, thermochromism, vapochromism, solvatochromism, and piezochromism [10, 11, 12, 13, 14, 15, 16, 17, 18, 19, 20, 21]. in response to various external stimuli. Over the past few years, we have developed a series of phosphorescent *C*‐centered hexagold(I) clusters protected by *N*‐heterocyclic carbene (NHC) [22, 23, 24, 25, 26, 27, 28], and reported that certain clusters exhibit vapochromism and mechanochromism in response to several organic solvents and mechanical stress [29]. This cluster exhibits a large photoluminescence (PL) wavelength shift in the solid state due to small changes in the conformation of the wingtip groups of the NHC ligands and the Au^I^···Au^I^ distance [29].

**FIGURE 1:**
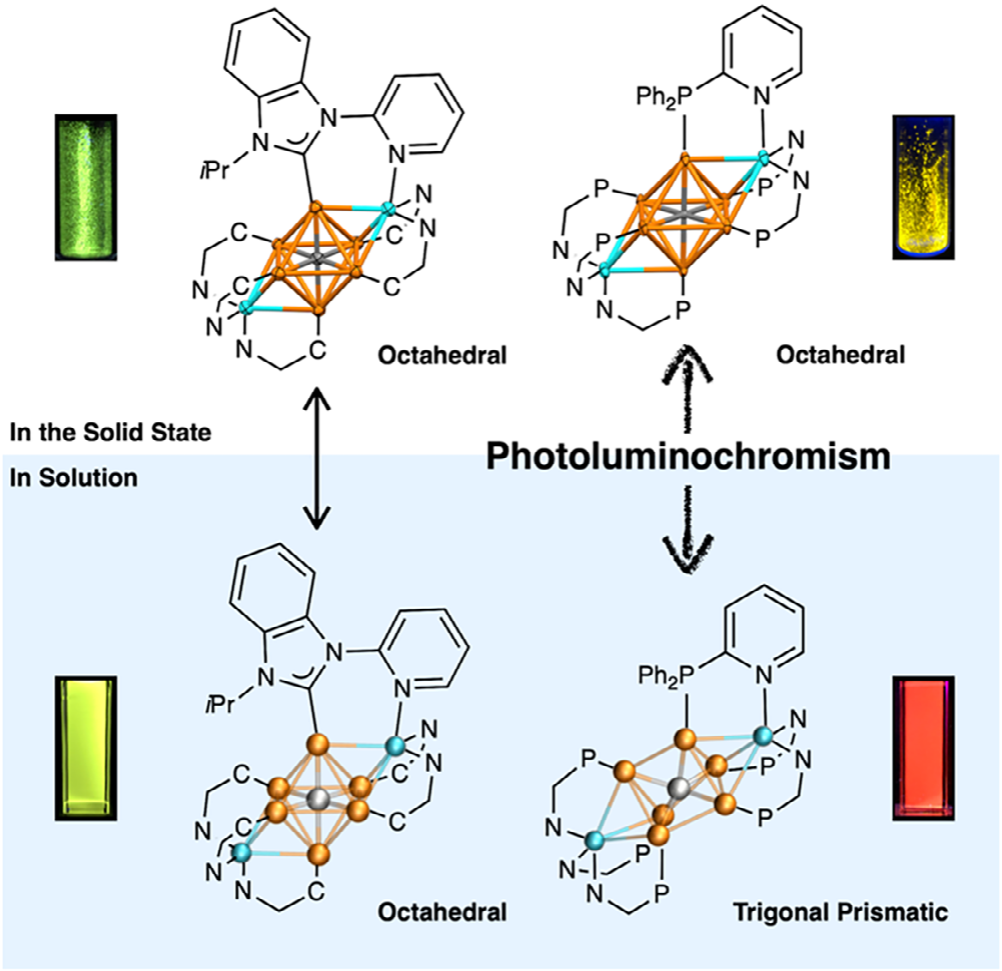
Schematic diagram of this study. Carbon(C)‐centered heterometallic Au^I^ clusters bearing *N*‐heterocyclic carbene (NHC) ligands could maintain identical molecular structures in both the solid and solution states, while the same clusters supported by phosphine ligands exhibit chromic PL, as supported by EXAFS and ScXRD analysis and DFT/TD‐DFT calculations. C^N and P^N represent the NHC ligand **BIPy** and the phosphine ligand dppy, respectively. Color code: orange, Au; cyan, Cu; gray, C.

There are many excellent examples of heterometallic clusters that exhibit PL in both the solid state and solution [10, 30, 31, 32, 33, 34, 35, 36, 37, 38, 39, 40]. However, all the examples of molecular chromism are related to the behaviors of molecules either in the solid or solution states. To our knowledge, there are no examples in which chromism has been observed during the dissolution process of the crystals in a solvent. During this process, changes in the crystalline packing and solvation structures can significantly affect the optical properties, and knowledge of the structure‐property relationship provides important design guidelines for the development of chromic materials. Here, we report the dissolution/crystallization‐induced photoluminochromism (PLC, Figure 1). A series of clusters, [(C)(Au^I^‐L)_6_M_2_](BF_4_)_4_ (**1**, L = *N*‐isopropyl‐*N*’‐2‐pyridylbenzimidazolylidene (**BIPy**), M = Cu^I^; **2**, L = 2‐pyridyldiphenylphosphine (dppy), M = Cu^I^; **3**, L = **BIPy**, M = Ag^I^; **4**, and L = dppy, M = Ag^I^), all share a bicapped octahedral structure in the crystalline state and show greenish‐yellow PL. After dissolution, a large bathochromic shift (> 100 nm) was observed in PL for phosphine‐protected clusters, and the luminescence properties were reversibly restored by recrystallization. Notably, these chromic properties are ligand‐specific, and are suppressed in **1** and **3** by using the *N*‐heterocyclic carbene **BIPy** as a ligand. To clarify the origin of this chromism, the structures of the clusters in solution were investigated by Au L_3_‐edge extended X‐ray fine structure (EXAFS) analysis, which showed that the chromism was accompanied by changes in the coordination number (C.N.) of the gold atoms, suggesting that the chromism may be caused by structural changes of the core part of the clusters. Finally, we focused on the conformational isomer of **2**, a bicapped trigonal prism [(C)(Au^I^‐dppy)_6_Cu^I^_2_](BF_4_)_4_ (2*), which indeed exhibits red PL in both the solid and solution states. Combining these experimental results with density functional theory (DFT) and timedependent (TD)‐DFT calculations, we clarified that clusters **2** and **4**, which adopt an octahedral structure in the solid state, are twisted into a triangular prism structure in solution, resulting in a bathochromic shift in the emission wavelength, and that this process is reversible. In this study, we have determined the molecular structures in solution of a series of heterometallic gold(I) clusters that exhibit ligand‐specific luminescence based on solid evidence, and discovered a reversible PLC phenomenon between the solid and solution states. This is expected to provide a new opportunity for the development of new types of metal cluster materials.

## 2 Results and Discussion

### 2.1 Synthesis, Crystallization and ScXRD Structure Analysis

Gold(I)‐copper(I) clusters [(C)(Au^I^‐**BIPy**)_6_Cu^I^_2_](BF_4_)_4_ (**1**) and [(C)(Au^I^‐dppy)_6_Cu^I^_2_](BF_4_)_4_ (**2**) were synthesized via a route similar to those of [(C)(Au^I^‐**BIPy**)_6_Ag^I^_2_](BF_4_)_4_ (**3**) and [(C)(Au^I^‐dppy)_6_Ag^I^_2_](BF_4_)_4_ (**4**), respectively (Scheme S1) [26, 37]. Typically, the hexagold(I) cluster [(C)(Au^I^‐**BIPy**)_6_](BF_4_)_2_ or [(C)(Au^I^‐dppy)_6_](BF_4_)_2_ was dissolved in dry CH_2_Cl_2_, and then three equivalents of [Cu^I^(CH_3_CN)_4_]BF_4_ were added. After stirring for 5 min, the mixture was filtered, and the filtrate was gently layered with dry Et_2_O. After about two weeks, pale yellow crystals of **1** and orange crystals of **2** were obtained, respectively.

**FIGURE 2:**
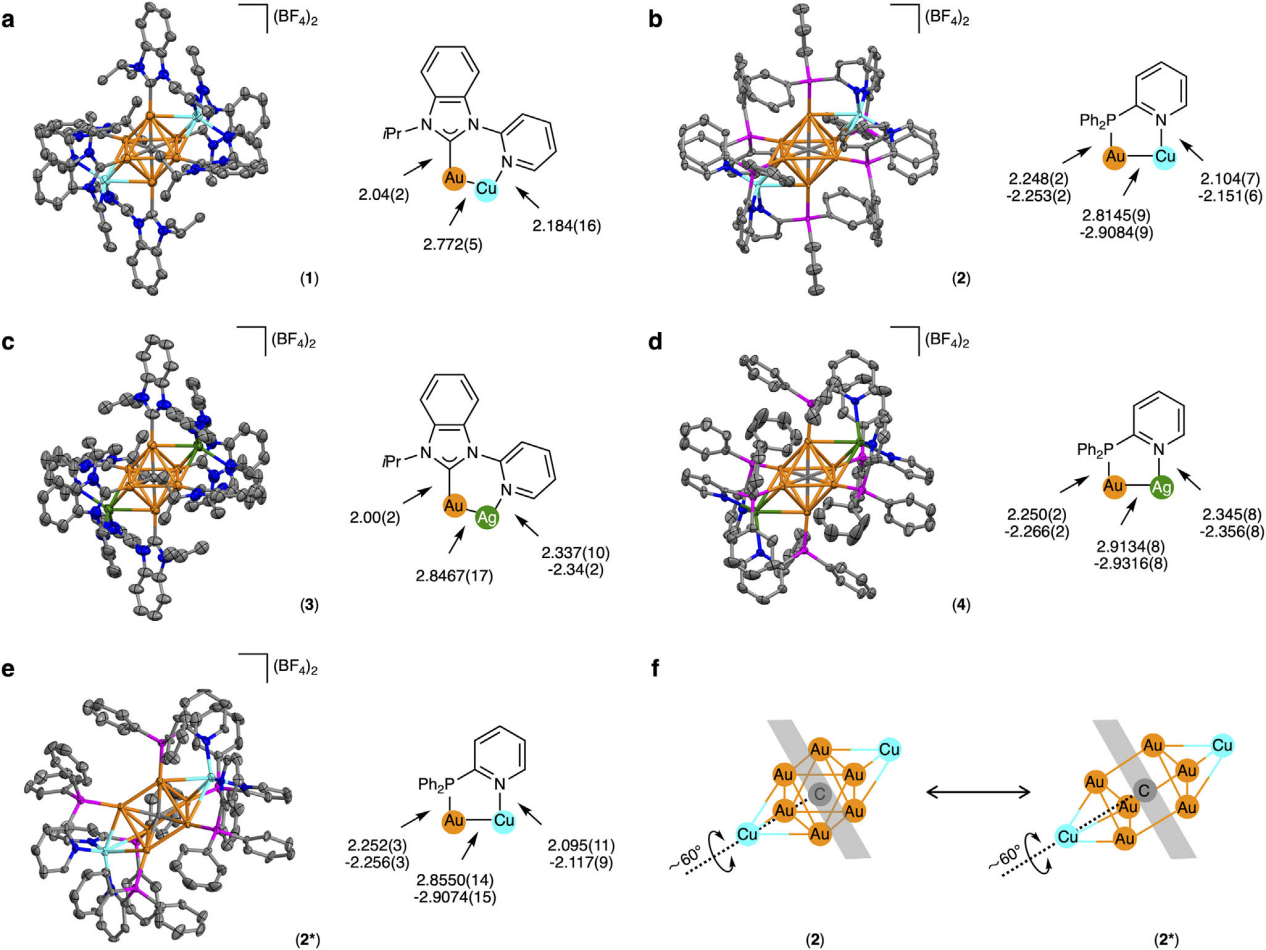
Molecular structures of heterometallic gold(I) clusters. (a)–(e) Molecular structures (BF_4_^−^ anions are simplified), ligand structures, and key structural parameters (Å) of **1**–**4** and 2*, respectively. (f) Simplified scheme showing the structural correlation between **2** and 2*. Color code: orange, Au; cyan, Cu; green, Ag; gray, C; blue, N; magenta, P.

The molecular structures of clusters **1** and **2** were determined by single crystal X‐ray diffraction (ScXRD) structural analysis [41]. As shown in Figure 2a–d, both **1** and **2** have a bicapped octahedral framework, which is very similar to those of **3** and **4** [26, 37]. However, because the atomic radius of copper ion is smaller than that of silver ion, the coordination environments of the copper ions in **1** and **2** are more compact than those of the silver ions in **3** and **4**, respectively. For example, the Au···Cu distances in **1** and **2** are 2.772(5) and 2.8145(9)–2.9084(9) Å, respectively, which are significantly shorter than the Au···Ag distances in **3** (2.8467(17) Å) and **4** (2.9134(8)–2.9316(8) Å), respectively. In addition, the N─Cu bond lengths in **1** and **2** are also shorter than the N─Ag bond lengths in **3** and **4**, respectively. On the other hand, comparison of the structures of **BIPy**‐protected **1** and **3**, and dppy‐protected **2** and **4** revealed that the coordination of the phosphine ligands on the cluster surface is generally weaker than those of carbene ligands. In other words, the **BIPy**‐protected Au‐Cu cluster **1** shows the most compact structure among **1**–**4** because the Au─C (carbene) bond distances in **1** and **3** are approximately 10% shorter than the Au─P bond distances in **2** and **4**. Another notable structural feature is the intramolecular Au···H−C interactions in **1** [42, 43, 44]. Cluster **1** has an extremely short gold‐hydrogen distance of 2.513 Å, which is the shortest among the hexagold(I) clusters (Figure S1). Other structural parameters of **1**–**4**, such as the Au─C (central C^4−^) and the Au···Au distances, are nearly comparable (Table S1).

The solution‐phase structures of **1** and **2** were characterized by NMR spectroscopy, MS spectrometry, and UV‐vis absorption spectroscopy, confirming the formation of heterometallic clusters. For example, in the ^1^H NMR spectrum of **1**, the signal at 1.49 ppm corresponding to the methyl group of [(C)(Au^I^‐**BIPy**)_6_](BF_4_)_2_ splits into two (1.31 and 1.41 ppm, Figure S2). This indicates that the pyridyl groups interact with secondary Cu^I^ ions, resulting in a preferred orientation of the methyl group, which is similar to related gold‐silver clusters such as **3** [26, 27]. For **2**, an intense signal corresponds to [(C)(Au^I^‐dppy)_6_Cu^I^](BF_4_)^2+^ was observed in the ESI‐MS spectrum (calcd.: 1461.6, found: 1461.7; Figure S8), providing strong evidence that the gold‐copper clusters were generated.

Notably, slow evaporation of a mixture of [(C)(Au^I^‐dppy)_6_](BF_4_)_2_ and [Cu^I^(CH_3_CN)_4_]BF_4_ in CH_2_Cl_2_/CH_3_OH/*n*‐hexane afforded red platelet crystals of 2* (Figure S11). This is the conformational isomer of **2**, which has the same chemical formula as **2**, [(C)(Au^I^‐dppy)_6_Cu^I^_2_](BF_4_)_4_ (Figure 2e). Cluster 2* was found to have a bicapped trigonal prismatic framework, unlike **2**, which has a bicapped octahedral framework [45]. Specifically, the central CAu^I^_6_ is twisted by approximately 60° from **2** to 2*, and the number of observed aurophilic interactions is reduced from 12 to 9 (Figure 2f). As a result, the Au─C (central C^4−^) bonds become slightly longer, and the Au···Au distances are significantly shorter, compensating for the stability of the whole cluster (Table S1). Other structural parameters of 2* were almost identical to those of **2**, but it was unexpected that the ^31^P NMR and UV‐vis absorption spectra of 2* were completely consistent with those of **2** (Figures S13–S18). Furthermore, recrystallization of 2* under the crystallization conditions of **2** produced only **2**. These results prompted us to investigate whether there are structural differences or interconversions between **2** and 2* in different states.

### 2.2 Photoluminescence (PL) and PLC

**FIGURE 3:**
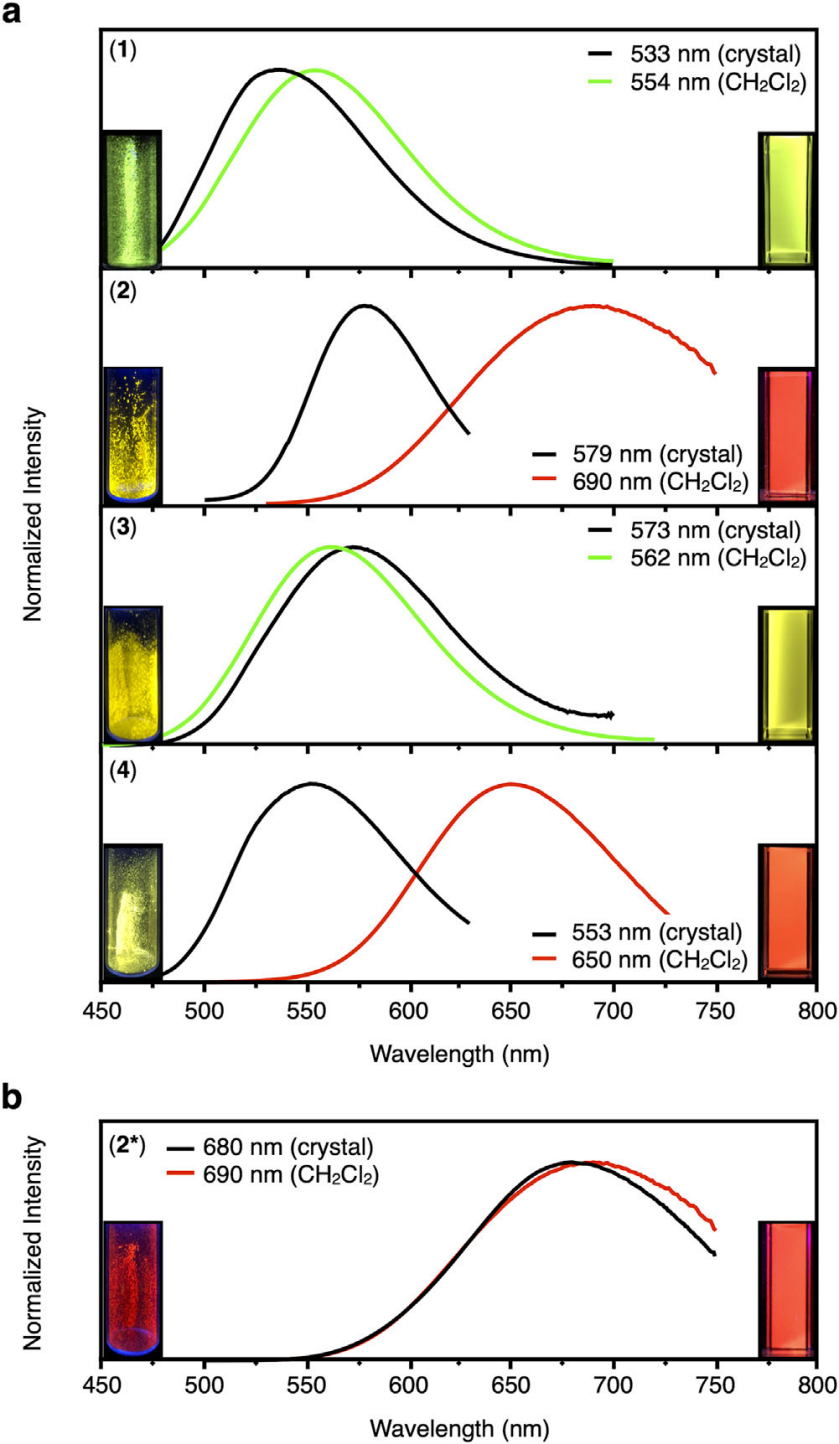
PL spectra of heterometallic gold(I) clusters. PL spectra of clusters **1**–**4** (a) and 2* (b) in the crystalline solid state and in CH_2_Cl_2_ at 300 K. Insets: corresponding photographs taken at room temperature.

Clusters **1**–**4** exhibit distinct PL properties in both crystalline and solution states under ambient conditions. Crystals of **1**–**4** show strong greenish‐yellow PL with PL maxima in the range from 533 to 579 nm (Figure 3a). However, in CH_2_Cl_2_, the PL of phosphine‐protected **2** and **4** was significantly red‐shifted by approximately 100 nm to deep red, whereas only a slight change was observed for NHC‐protected **1** and **3** (Figures S19–S22) [26, 37]. The photoluminescence quantum yields (PLQYs) for the copper‐containing **1** and **2** were 0.05 and 0.15 in CH_2_Cl_2_, respectively, which were significantly lower than those for the silver‐containing **3** (0.86) and **4** (0.31). Meanwhile, the PL lifetimes of **1** (1.15 µs) and **2** (3.54 µs) were comparable to those of **3** (1.66 µs) and **4** (3.74 µs), respectively. Table S2 shows radiative and nonradiative rate constants calculated from the PLQYs and PL lifetimes of **1**–**4**. Among **1**–**4**, the PL of **1** has a relatively low radiative rate constant and the highest nonradiative rate constant, which is in sharp contrast to the PL of **3**, which is also protected by **BIPy**. This is probably due to a slight mismatch in size between the copper ions and the structure of [(C)(Au^I^‐**BIPy**)_6_](BF_4_)_2_.

On the other hand, cluster 2* exhibits deep red PL both in the solid state and in CH_2_Cl_2_. Specifically, the maximal PL wavelengths in the two states of 2* are 680 and 690 nm, respectively, and the PL profile of 2* in CH_2_Cl_2_ is almost the same as that of **2** (Figures S23–S25). Considering that clusters **2** and 2* can be isomers of each other, the PL properties of **2** and 2* in the solid state are highly dependent on the core structures of the clusters, but in CH_2_Cl_2_, clusters **2** and 2* are thought to have the same structure, that is, the bicapped, octahedral **2** in the solid state is twisted into the same bicapped trigonal prism as 2* in CH_2_Cl_2_.

To verify this observation, we first measured the PL of the clusters in CH_2_Cl_2_ at low temperatures (Figures S26,27). For **BIPy**‐protected **1** and **3**, the PL profiles remained almost unchanged when the temperature was decreased from 300 to 180 K, whereas for dppy‐protected **2** and **4**, the PL maxima significantly blue‐shifted by about 50 nm upon cooling. As expected, cluster 2* exhibited a blue shift at 180 K similar to that of **2**. We next investigated the solvent effects on the PL of the clusters (Figures S28–S30). In CH_3_OH, (CH_3_)_2_CO and a mixed solvent of CH_2_Cl_2_/CH_3_OH (v:v = 9:1), all the clusters showed PL comparable to those in CH_2_Cl_2_. The PL of copper‐containing clusters **1**, **2**, and 2* was expected to be quenched by CH_3_CN, which has strong coordination ability for copper ions, whereas silver‐containing clusters **3** and **4** were expected to be robust. Furthermore, we tried to look into the aggregation processes of the clusters (Figure S31). In CH_2_Cl_2_/*n*‐hexane (v:v = 1:9), clusters **1**, **3**, and **4** show almost the same emissions as those in the solid states, in which the clusters adopted bicapped octahedral core structures. Notably, cluster **2** exhibited PL in a broad range from 550 to 750 nm, suggesting a dual peak corresponding to the PL of intermediate structures between octahedral **2** and triangular prismatic 2* in the solid states, respectively (*λ*_max_^em^ = 579 and 680 nm; Figure S31). Similarly, in the case of 2*, two types of structures coexist in the presence of *n*‐hexane_._ Next, we prepared PMMA (polymethyl methacrylate) films using clusters **1**–**4**, and 2*, and measured their PL spectra (Figure S32). The films showed PL in the yellowish‐green to yellow region nearly equivalent to the cluster crystals. Meanwhile, the PMMA film of 2* also showed yellowish‐green PL, which is quite different from the deep red PL of the crystals of 2*. This is similar to the recrystallization of 2* under the crystallization conditions that yield only **2**, which emits yellowish‐green light. The trigonal prismatic framework of 2* does not appear to be stabilized in PMMA. Overall, these observations confirmed that the structures of **2** and **4** in solution are likely metastable at room temperature.

Based on the molecular structures of **1**–**4** and 2* determined by ScXRD, their PL in the solid and solution states etc., we propose a novel PLC phenomenon induced by dissolution/crystallization as follows. The phosphine‐protected heterometallic clusters **2** and **4** show yellow PL in the solid state, but when dissolved in CH_2_Cl_2_, the PL is red shifted by about 100 nm. Taking into account the structure and PL properties of 2*, it can be concluded that this chromism is originated from the structural twist of the metal core in **2** and **4**. On the other hand, the heterometallic clusters **1** and **3**, which use the more rigid NHC ligand **BIPy**, form sterically compact and dense structures, which prevent structural twisting during the dissolution process.

### 2.3 EXAFS Studies

To clarify the geometric structures of **1**–**4** and 2* in solution, their local structures in the solid and solution states were investigated by Au L_3_‐edge XAFS. Furthermore, curve fitting analysis of the Fourier‐transformed EXAFS (FT‐EXAFS) spectra was performed to determine the structural parameters such as the coordination numbers (C.N.) and bonding distances (*r*) of **1**–**4** and 2* in the solid and solution states [46, 47, 48, 49, 50, 51, 52, 53, 54].

**FIGURE 4:**
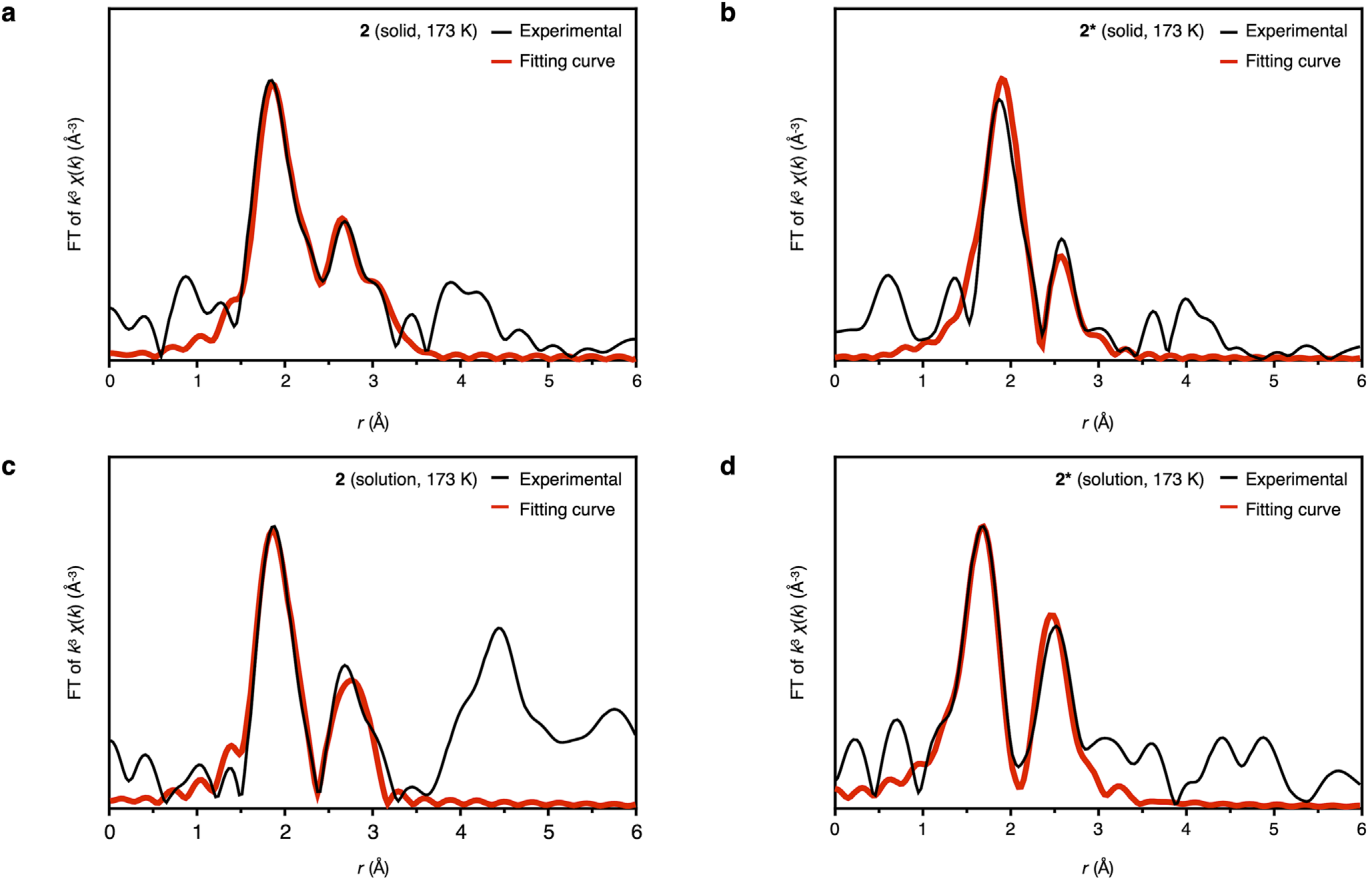
EXAFS spectra. Au L_3_‐edge FT‐EXAFS spectra of (a) **2** in the solid state; (b) 2* in the solid state; (c) **2** in solution (CH_2_Cl_2_/CH_3_OH, v:v = 9:1) and (d) 2* in solution (CH_2_Cl_2_/CH_3_OH, v:v = 9:1) at 173 K, respectively.

First, the FT‐EXAFS spectra of solid samples **1**–**4** and 2* showed that Au─X (X = C and/or P) and Au···Au distances were observed in the range of *r* = 1.5–3.0 Å, as shown in Figures 4 and S33–S37. The Au···Au peak intensity in the range of *r* = 2.5–3.0 Å gradually decreased with increasing measurement temperature, which is due to the large thermal fluctuation of the Au···Au interactions within the clusters. As summarized in Table 1, the C.N. and *r* values are in good accordance with those obtained from ScXRD analysis, especially when measured at the low temperature of 10 K. Curve fitting results indicate that the bicapped octahedral framework of **1**–**4** has a C.N. of about 4 for Au···Au and long Au···Au distances (> 2.90 Å), whereas the bicapped trigonal prismatic framework of 2* has a C.N. of about 3 and short Au···Au distances (< 2.90 Å). Similar results were obtained from measurements at 173 and 300 K. This means that the curve‐fitting analysis can distinguish between different core structures of the clusters.

**TABLE 1:** Coordination numbers (C.N.) of Au atoms and Au···Au distances (*r*) in **1**–**4** and 2* in the solid state and in solution (CH_2_Cl_2_/CH_3_OH, v:V = 9:1) at different temperatures obtained by curve‐fitting analysis of the Au L_3_‐edge EXAFS spectra.

	In the solid state					
	300 K		173 K		10 K	
	C.N.	*r* (Å)	C.N.	*r* (Å)	C.N.	*r* (Å)
**1**	3.8 ± 0.7	2.93 ± 0.10	3.9 ± 0.6	2.95 ± 0.07	4.4 ± 0.4	2.96 ± 0.05
**2**	4.4 ± 0.3	2.94 ± 0.05	4.2 ± 0.5	2.94 ± 0.07	4.0 ± 0.2	2.95 ± 0.03
**3**	3.9 ± 0.8	2.96 ± 0.13	4.0 ± 0.9	2.93 ± 0.10	3.9 ± 0.4	2.93 ± 0.05
**4**	4.2 ± 0.8	2.93 ± 0.06	4.0 ± 0.5	2.94 ± 0.07	4.1 ± 0.2	2.92 ± 0.03
2*	3.0 ± 0.4	2.82 ± 0.06	3.1 ± 0.5	2.90 ± 0.07	2.8 ± 0.2	2.88 ± 0.03
	**In solution**				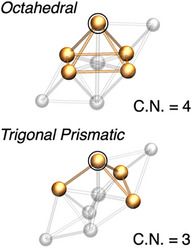	
	**300 K**		**173 K**			
	**C.N**.	***r*** **(Å)**	**C.N**.	***r*** **(Å)**		
**1**	4.3 ± 0.9	2.94 ± 0.13	3.6 ± 0.6	2.99 ± 0.06		
**2**	2.9 ± 0.4	2.82 ± 0.06	3.4 ± 0.3	2.89 ± 0.04		
**3**	4.3 ± 0.9	2.93 ± 0.06	3.5 ± 0.6	2.99 ± 0.08		
**4**	3.1 ± 0.5	2.86 ± 0.07	3.4 ± 0.7	2.91 ± 0.10		
2*	2.9 ± 0.3	2.85 ± 0.05	3.5 ± 0.6	2.89 ± 0.05		

Next, the FT‐EXAFS spectra of **1**–**4** and 2* in solution (CH_2_Cl_2_/CH_3_OH, v:v = 9:1) were measured at 173 and 300 K, and the curve‐fitting analysis results are summarized in Table 1 and Figures S33–S37. For **BIPy**‐protected **1** and **3** with a bicapped octahedral framework, the C.N. and *r* values of the Au···Au interactions were about 4 and > 2.90 Å, respectively, which were almost comparable to those in the solid state. In the case of dppy‐protected 2* with a bicapped trigonal prismatic framework, the C.N. and *r* values of the Au···Au interactions in solution were found to be almost the same as those in the solid state (about 3 and < 2.90 Å, respectively). That is, the structures of **1**, **3**, and 2* are maintained in the dissolution process. On the other hand, the structural parameters of **2** and **4** changed significantly after dissolution. Specifically, the C.N. and *r* values of the Au···Au interactions in solution for **2** and **4** were found to be about 3 and generally < 2.90 Å, respectively, which are significantly different from those for **2** and **4** in the solid state, but similar to those for 2*, which has a bicapped trigonal prismatic framework in both the solid and solution states. These results strongly suggest that the geometric structures of **2** and **4** are isomerized from a bicapped octahedral framework to a bicapped triangular prismatic framework in the process of dissolution.

### 2.4 Theoretical Calculations

Density functional theory (DFT) and time‐dependent (TD)DFT calculations of the heterometallic gold(I) clusters were performed to unravel the origin of the experimentally observed structural changes and PLC. Geometry optimizations of **1**–**4** well reproduced the ScXRD structures, and the bond distances in the optimized structures are only slightly longer than those in the experimental data. We also examined the counterpart trigonal prismatic configurations denoted as 1*–4* (Figure S38 and Table S3).

The octahedral structure is always more stable than the trigonal prismatic structure in the gas phase, regardless of capping metal atoms (Cu^I^ or Ag^I^) and their coordinating ligands (*N*‐heterocyclic carbene or phosphine, Table S4). The results are consistent with the fact that the metal clusters with octahedral structures are easily characterized by ScXRD. Phosphine ligands tend to have a smaller energy difference between the two different structures compared to *N*‐heterocyclic carbene ligands. We also evaluated the solvent effect of the experimentally used mixed solvent on the stability of these structures. For cluster 2’/2* (Figure S38), the trigonal prismatic structure is 2.9 kcal/mol more stable than the octahedral structure in the mixed solvent (CH_2_Cl_2_/*n*‐hexane, v = 1:1, Table S4), whereas the other clusters prefer the octahedral structure. It should be noted here that this stability depends on the DFT functionals and the solvent (Table S5).

We then analyzed the C─Au bonds, as well as the Au···Au and Cu/Ag···Au interactions in these heterometallic clusters (Table S3). In the octahedral clusters, the C─Au bonds have bond lengths ranging from 2.13 to 2.18 Å and a bond order of approximately 0.37, indicating covalent character. The bond orders of Au···Au, Cu···Au, and Ag···Au are in the range of 0.10–0.17, 0.10–0.13, and 0.10–0.11, respectively, reflecting the internuclear distances of 2.94–3.32 Å, 2.90–3.10 Å, and 2.99–3.06 Å, respectively. As the bond orders of C─Au, Au···Au, and Cu/Ag···Au in the triangular prism clusters are similar to those in the octahedral clusters, a greater number of adjacent Au···Au interactions in the octahedral arrangement (12) compared to the triangular prism arrangement (9) is believed to contribute to the higher stability of the former. The bond orders of the Au···Au interactions in the triangular prism structures range from 0.15 to 0.17, slightly larger than in the octahedral clusters. This partially compensates for the reduced number of Au···Au interactions in the triangular prism clusters, thereby stabilizing the clusters.

**FIGURE 5:**
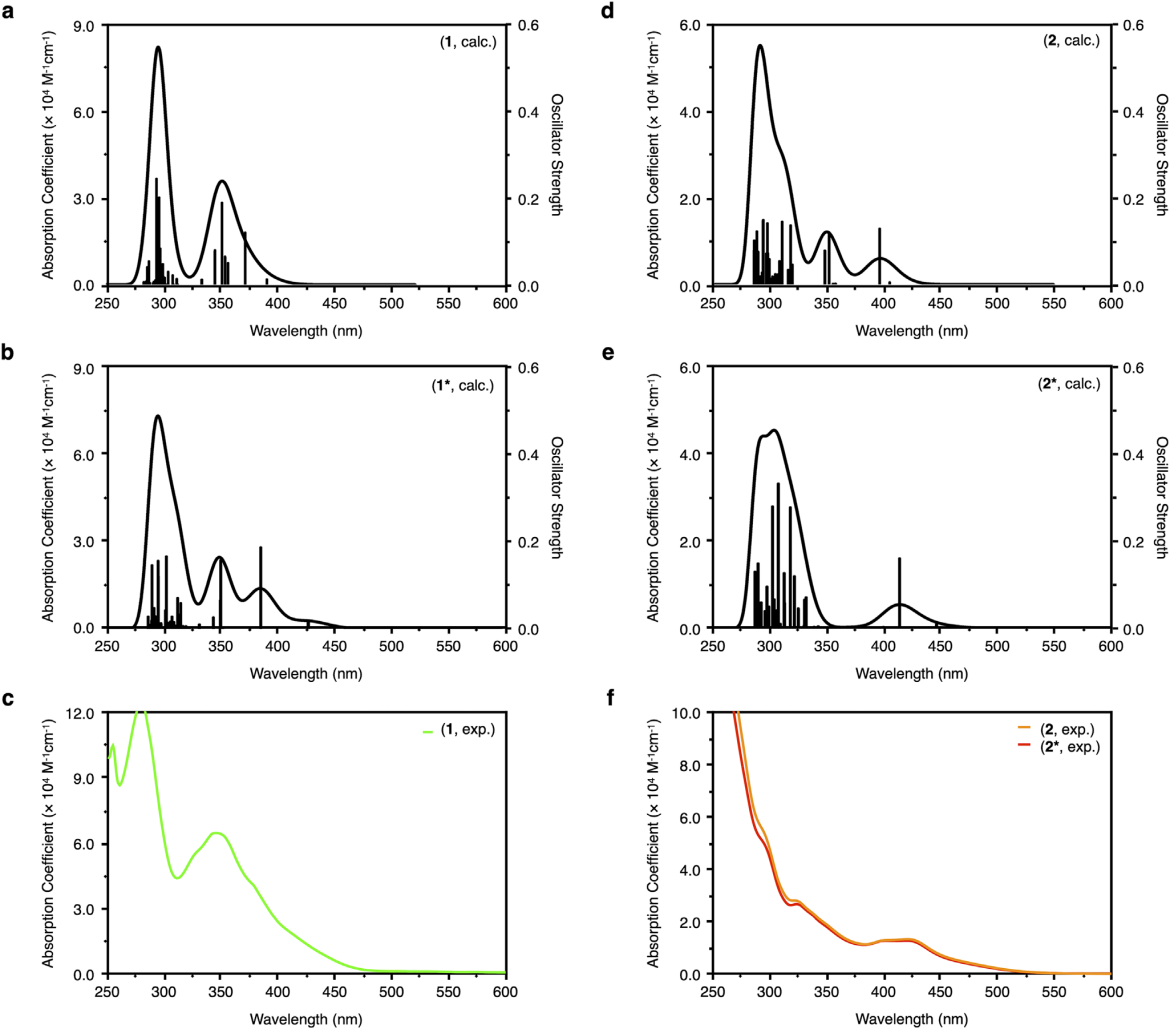
Calculated (by MN15) and experimental UV‐vis absorption spectra. Calculated UV‐vis absorption spectra of (a) **1** and (b) 1*; (c) Experimental UV‐vis absorption spectrum of **1**; Calculated UV‐vis absorption spectra of (d) **2** and (e) 2*; (f) Experimental UV‐vis absorption spectra of **2** and 2*.

Figure 5 shows the TD‐DFT simulated absorption spectra of **1**/1* and **2**/2*, and those of **3**/**3** ^*^ and **4**/**4** ^*^ are shown in Figure S39. Similar spectral characteristics are observed in the simulated spectra of **1** and 1*; however, the experimental spectrum of **1** more closely resembles the simulated spectrum of **1**, due to the presence of a single strong peak at 316 nm in **1**, compared to two peaks at 314 and 354 nm in 1*. The nearly overlapping experimental spectra of **2** and 2* suggest a structural transformation in CH_2_Cl_2_. While the simulated spectra are also similar, **2** shows an additional peak at 313 nm that is absent in 2*. The experimental spectra align more closely with the simulated spectrum of 2*, supporting the conclusion that cluster **2** converts to 2* in CH_2_Cl_2_, in agreement with the earlier discussion.

Here, we qualitatively characterize the absorption spectra of **1** and 2*. The calculated absorption spectrum of **1** shows a strong peak around 316 nm, primarily composed of two excitations at 316 and 339 nm. These can be correlated with a broad experimental peak near 350 nm, featuring a shoulder on the lower‐energy side. The excitation at 339 nm is attributed to the transition from HOMO−2 to LUMO (Table S6). Orbital composition analysis shows that the HOMO−2 consists of C‐center (24%), Au_6_ (45%), and BIPy (29%), while the LUMO is composed of Au_6_ (51%), Cu_2_ (14%), and BIPy (34%) (Table S7). This suggests a charge reorganization transition from the C‐center and the Au_6_ toward the metal kernel and shell ligands (Figure S40). The excitation at 316 nm arises from a linear combination of transitions involving two pairs of nearly degenerate orbitals (HOMO/HOMO−1 → LUMO+1/LUMO+2). Since LUMO+1 and LUMO+2 share similar orbital characteristics with the LUMO, this transition exhibits a charge‐transfer behavior similar to the 339 nm excitation. The most intense peak centered at 252 nm originates from multiple excitations and corresponds to the experimental absorption observed at 275 nm. The simulated absorption spectrum of 2* shows a weak peak at 388 nm, corresponding to the broad experimental absorption band around 420 nm. It originates from the HOMO−2 → LUMO transition and primarily exhibits charge‐transfer character from the C‐center and Au atoms (HOMO−2: C‐center 26%, Au_6_ 31%, dppy 40%) to the Au and Cu atoms (LUMO: Au_6_ 62%, Cu_2_ 12%, dppy 25%) (Tables S12, S13 and Figure S43).

Finally, the phosphorescence energies of the octahedral and triangular prism structures of all the heterometallic clusters examined were evaluated by DFT calculations of the triplet states (Table 2). The large redshift (0.14 eV) observed in PL spectrum of the triangular prism structure of 2* was well reproduced by DFT calculations to be 0.19 eV. The intermediate structure (**2^int^**) between **2** and 2* has a twist of about 30° around the CAu^I^_6_ core, indicating that the CAu^I^_6_ structure is important in controlling the phosphorescence. The phosphorescence of **4** observed in a previous study is also thought to originate from 4*. The calculated value (661 nm) reproduced the experimental one (650 nm) [26, 37].

**TABLE 2:** Calculated phosphorescence energies (*E*) and wavelengths (*λ*) of clusters (**1**–**4** and 1*–4*) in the gas phase. Available experimental values are also given.

	DFT		expt.	
Clusters	*E* (eV)	*λ* (nm)	*E* (eV)	*λ* (nm)
**1**	2.09	595	2.24	554
1*	1.92	647	—	—
**2**	2.15	577	1.80	690
**2^int^**	1.99	624	—	—
2*	1.96	633	1.80	690
**3**	2.07	600	2.21	562
3*	1.81	687	—	—
**4**	2.59	479	1.91	650
4*	1.87	661	—	—

## 3 Conclusion

In summary, we reported a new type of luminochromism in a series of heterometallic gold(I) clusters that is induced by dissolution/crystallization and shows ligand‐dependence. Although all the series of clusters exhibit greenish‐yellow PL in the solid state, and have a bicapped octahedral structure as shown by ScXRD analysis, their solutions show PL shifted in different directions by up to about 100 nm. The solution structures of these clusters were elucidated by EXAFS measurements and theoretical calculations, providing solid evidence that the twisting from an octahedral to a triangular prism structure is the fundamental cause of the bathochromic shift and that this process is reversible. Such clusters are expected to contribute greatly to the development of new types of PL materials that exhibit the luminochromic phenomenon.

## Conflicts of Interest

The authors declare no conflicts of interest.

## Supporting information



The authors have cited additional references within the Supporting Information [1–19].

**Supporting Information File 1**: chem70778‐sup‐0002‐Data.zip

## Data Availability

The data that support the findings of this study are available in the supplementary material of this article.
