# Supplementary material for: Hypercarbon‐Centered Gold(I)‐Copper(I) Clusters Exhibiting a Dissolution/Crystallization‐Induced Photoluminochromism
Source: Chemistry. 2026 Feb 24;32(11):e00011. doi: 10.1002/chem.202600011 (PMC13005659; doi:10.1002/chem.202600011)
Supplement: Supplementary file 1 — The authors have cited additional references within the Supporting Information [1–19]. [file CHEM-32-e00011-s002.pdf]

Supporting Information

Hypercarbon-Centered Gold(I)-Copper(I) Clusters Exhibiting A Dissolution/Crystallization-Induced Photoluminochromism

Zhen Lei,[a,d]†* Pei Zhao,[b]† Soichi Kikkawa,[c] Xiao-Li Pei,[a,e] Wen-Ting Liu,[d] Hitoshi Ube,[a,e] Seiji Yamazoe,[c]* Masahiro Ehara,[b]* and Mitsuhiko Shionoya[a,e]*

[a] Department of Chemistry, Graduate School of Science, The University of Tokyo, 7-3-1 Hongo, Bunkyo-ku, Tokyo 113-0033, Japan

[b] Research Center for Computational Science, Institute for Molecular Science and SOKENDAI, Myodaiji, Okazaki, Aichi 444-8585, Japan. E-mail: ehara@ims.ac.jp

[c] Department of Chemistry, Graduate School of Science, Tokyo Metropolitan University, Minami Osawa, Hachioji, Tokyo 192-0397, Japan. E-mail: yamazoe@tmu.ac.jp

[d] Current address: Fujian Provincial Key Laboratory of Advanced Inorganic Oxygenated Materials, College of Chemistry, Fuzhou University, Fuzhou 350108, P. R. China. E-mail: zhenlei@fzu.edu.cn

[e] Current address: Research Institute for Science and Technology, Tokyo University of Science, 2641 Yamazaki, Noda, Chiba 278-8510, Japan. E-mail: shionoya@rs.tus.ac.jp

† These authors contributed equally to this work.

Table of contents

1. Materials and reagents

2. Physical measurements and instrumentation

3. Synthesis and characterization

4. X-ray crystallography

5. X-ray absorption spectroscopy

6. Computation details

7. Supporting schemes, figures, and tables

Scheme S1. Synthesis and crystallization of clusters **1-4** and **2***.
Figure S1. Schematic illustration of intramolecular Au···H−C interactions in **1-4** and **2***
Table S1. Key structural parameters of **1-4**, **2***, and related $CAu^I_6M^I_2$ clusters (M = Ag, Cu)
Figure S2. ^{1}H NMR spectrum of **1** (500 MHz, CD_2Cl_2/CD_3OD (9:1, v:v), 300 K)
Figure S3. ^{13}C NMR spectrum of **1** (125 MHz, CD_2Cl_2/CD_3OD (9:1, v:v), 300 K)
Figure S4. ^{1}H NMR spectrum of **2** (500 MHz, CD_2Cl_2/CD_3OD (9:1, v:v), 300 K)
Figure S5. ^{31}P NMR spectrum of **2** (202 MHz, CD_2Cl_2/CD_3OD (9:1, v:v), 300 K)
Figure S6. ^{13}C NMR spectrum of **2** (125 MHz, CD_2Cl_2/CD_3OD (9:1, v:v), 300 K)
Figure S7. ESI MS spectrum of **1** in CH_2Cl_2
Figure S8. ESI MS spectrum of **2** in CH_2Cl_2
Figure S9. UV-vis absorption spectrum of **1** in CH_2Cl_2
Figure S10. UV-vis absorption spectrum of **2** in CH_2Cl_2
Figure S11. A photo of crystals of **2** and **2*** under sunlight
Figure S12. ^{1}H NMR spectrum of **2*** (500 MHz, CD_2Cl_2/CD_3OD (9:1, v:v), 300 K)
Figure S13. ^{31}P NMR spectrum of **2*** (202 MHz, CD_2Cl_2/CD_3OD (9:1, v:v), 300 K)
Figure S14. ^{13}C NMR spectrum of **2*** (125 MHz, CD_2Cl_2/CD_3OD (9:1, v:v), 300 K)
Figure S15. ESI MS spectrum of **2*** in CH_2Cl_2
Figure S16. UV-vis absorption spectrum of **2*** in CH_2Cl_2
Figure S17. Comparison of UV-vis absorption spectra of **1** and **3** in CH_2Cl_2
Figure S18. Comparison of UV-vis absorption spectra of **2**, **2*,** and **4** in CH_2Cl_2
Figure S19. Excitation and photoluminescence spectra of **1** in the solid state
Figure S20. Excitation and photoluminescence spectra of **2** in the solid state
Figure S21. Excitation and photoluminescence spectra of **1** in CH_2Cl_2
Figure S22. Excitation and photoluminescence spectra of **2** in CH_2Cl_2
Table S2. photoluminescence QYs, lifetimes, and radiative and non-radiative rate constants of **1-4** and **2*** in CH_2Cl_2
Figure S23. Excitation and photoluminescence spectra of **2*** in the solid state
Figure S24. A photo of crystals of **2** and **2*** under 365 nm excitation
Figure S25. Excitation and photoluminescence spectra of **2*** in CH_2Cl_2
Figure S26. Photoluminescence spectra of (a) **1**, (b) **2**, (c) **3**, (d) **4**, and (e) **2*** in CH_2Cl_2 at 300, 270, 240, 210, and 180 K, respectively

Figure S27. Comparison of the photoluminescence spectra of (a) **1-4** and (b) **2*** in CH_2Cl_2 at 180 and 300 K
Figure S28. Excitation and photoluminescence spectra of (a) **1**, (b) **2**, (c) **3**, (d) **4**, and (e) **2*** in CH_3OH at 300 K
Figure S29. Excitation and photoluminescence spectra of (a) **1**, (b) **2**, (c) **3**, (d) **4**, and (e) **2*** in CH_3COCH_3 at 300 K
Figure S30. Excitation and photoluminescence spectra of (a) **3** and (b) **4** in CH_3CN, and (c) **1**, (d) **2**, (e) **3**, (f) **4**, and (g) **2*** in CH_2Cl_2/CH_3OH (9:1, v:v) at 300 K
Figure S31. Comparison of the photoluminescence spectra of (a) **1-4** and (b) **2*** in CH_2Cl_2 and in CH_2Cl_2/*n*-hexane (1:9, v:v) at 300 K
Figure S32. Comparison of the photoluminescence spectra of (a) **1-4** and (b) **2*** in the crystalline solid state and cluster-containing PMMA films at 300 K
Figure S33. Au L_3-edge EXAFS oscillations, Fourier-transformed EXAFS spectra, and curve-fitting analysis results of Au L_3-edge EXAFS spectra of **1** in the solid state and in solution (CH_2Cl_2/CH_3OH, (9:1, v:v)) at different temperatures.
Figure S34. Au L_3-edge EXAFS oscillations, Fourier-transformed EXAFS spectra, and curve-fitting analysis results of Au L_3-edge EXAFS spectra of **2** in the solid state and in solution (CH_2Cl_2/CH_3OH, (9:1, v:v)) at different temperatures.
Figure S35. Au L_3-edge EXAFS oscillations, Fourier-transformed EXAFS spectra, and curve-fitting analysis results of Au L_3-edge EXAFS spectra of **3** in the solid state and in solution (CH_2Cl_2/CH_3OH, (9:1, v:v)) at different temperatures.
Figure S36. Au L_3-edge EXAFS oscillations, Fourier-transformed EXAFS spectra, and curve-fitting analysis results of Au L_3-edge EXAFS spectra of **4** in the solid state and in solution (CH_2Cl_2/CH_3OH, (9:1, v:v)) at different temperatures.
Figure S37. Au L_3-edge EXAFS oscillations, Fourier-transformed EXAFS spectra, and curve-fitting analysis results of Au L_3-edge EXAFS spectra of **2*** in the solid state and in solution (CH_2Cl_2/CH_3OH, (9:1, v:v)) at different temperatures.
Figure S38. Comparison of the crystal structures (left) of compounds (a) **1**, (b) **2**, (c) **3**, (d) **4**, and (e) **2***.
Table S3. Bond lengths (*d*, in Å) and Wiberg bond orders (WBO) of C–Au, Au···Au, and Au···Cu/Ag in the octahedral and triangular prism structures.
Table S4. Relative energies (in kcal mol^{-1}) of the octahedral (**1-4**) and triangular prism (**1*-4***) structures.
Table S5. Functional dependence of relative energies of the clusters (**1-4** and **1*-4***) in the gas phase and various solvent phases.
Figure S39. Comparison of the calculated (by MN15) and experimental UV-vis absorption spectra.
Table S6. Excited states of **1** with oscillator strength (*f*) greater than 0.02
Figure S40. Selected molecular orbitals of **1** (isovalue = 0.02)
Table S7. Orbital composition analysis with Mulliken partition for selected molecular orbitals of **1**
Table S8. Excited states of **1*** with oscillator strength (*f*) greater than 0.02
Figure S41. Selected molecular orbitals of **1*** (isovalue = 0.02)
Table S9. Orbital composition analysis with Mulliken partition for selected molecular orbitals of **1***
Table S10. Excited states of **2** with oscillator strength (*f*) greater than 0.02

Figure S42. Frontier molecular orbitals of **2** (isovalue = 0.02)
Table S11. Orbital composition analysis with Mulliken partition for selected molecular orbitals of **2**
Table S12. Excited states of **2*** with oscillator strength (*f*) greater than 0.02
Figure S43. Selected molecular orbitals of **2*** (isovalue = 0.02)
Table S13. Orbital composition analysis with Mulliken partition for selected molecular orbitals of **2***
Table S14. Excited states of **3** with oscillator strength (*f*) greater than 0.02
Figure S44. Selected molecular orbitals of **3** (isovalue = 0.02)
Table S15. Orbital composition analysis with Mulliken partition for selected molecular orbitals of **3**
Table S16. Excited states of **3*** with oscillator strength (*f*) greater than 0.02
Figure S45. Selected molecular orbitals of **3*** (isovalue = 0.02)
Table S17. Orbital composition analysis with Mulliken partition for selected molecular orbitals of **3***
Table S18. Excited states of **4** with oscillator strength (*f*) greater than 0.02
Figure S46. Selected molecular orbitals of **4** (isovalue = 0.02)
Table S19. Orbital composition analysis with Mulliken partition for selected molecular orbitals of **4**
Table S20. Excited states of **4*** with oscillator strength (*f*) greater than 0.02
Figure S47. Selected molecular orbitals of **4*** (isovalue = 0.02).
Table S21. Orbital composition analysis with Mulliken partition for selected molecular orbitals of **4***

8. References

1. Materials and reagents

All commercially available reagents were used as received. Benzimidazole, 2-bromopyridine, potassium carbonate, sodium carbonate, anhydrous magnesium sulfate, chloroauric acid trihydrate, potassium hydroxide, sodium tetrafluoroborate, trimethylamine and all the dry solvents were purchased from FUJIFILM Wako Pure Chemical Corporation. 2-Iodopropane, tetrahydrothiophene (tht) and tetrakis(acetonitrile)copper tetrafluoroborate were obtained from Tokyo Chemical Industry Co., LTD. Trimethylsilyldiazomethane (2.0 M in *n*-hexane) was purchased from Sigma-Aldrich.

2. Physical measurements and instrumentation

NMR data were recorded on a Bruker Avance III spectrometer (500 MHz). When CD_2Cl_2 was used, a mono-protonated solvent signal of CD_2Cl_2 ($CD\mathit{H}Cl_2$, 5.32 ppm) and a solvent signal of CD_2Cl_2 ($^{13}\mathit{C}D_2Cl_2$, 53.84 ppm) were used as the internal standards for ^{1}H and ^{13}C NMR measurements, respectively. Abbreviations: s, singlet; d, doublet; sept, septet; dd, double doublet; br, broad. ESI-TOF-MS spectrum were recorded on a Micromass LCT Premier XE. Experimental conditions: ion mode, positive; desolvation temperature, 150 °C; source temperature, 80 °C. UV-vis spectra were recorded on a Jasco V-770 spectrophotometer. Photoluminescence was measured on an Edinburgh FLS980 and a Jasco FP-8300 spectrofluorometer. Photoluminescence quantum yields were determined on a Hamamatsu C9920-02G spectrometer. Photoluminescence lifetimes were determined on a Hamamatsu C11367-02 spectrometer. Elemental analysis was conducted in the Microanalytical Laboratory, Department of Chemistry, Graduated School of Science, the University of Tokyo.

3. Synthesis and characterization

Clusters $[(C)(Au^{I}$-**BIPy**$)_6](BF_4)_2$ (**BIPy** = *N*-isopropyl-*N'*-2-pyridylbenzimidazolylidene),[1] $[(C)(Au^{I}\text{-dppy})_6](BF_4)_2$ (dppy = 2-pyridyldiphenylphosphine),[2] $[(C)(Au^{I}$-**BIPy**$)_6Ag^{I}_2](BF_4)_4$ (**3**),[1] and $[(C)(Au^{I}\text{-dppy})_6Ag^{I}_2](BF_4)_4$ (**4**)[2] were synthesized according to the literature procedures.

Clusters $[(C)(Au^{I}$-**BIPy**$)_6Cu^{I}_2](BF_4)_4$ (**1**), $[(C)(Au^{I}\text{-dppy})_6Cu^{I}_2](BF_4)_4$ (**2**, octahedral), and $[(C)(Au^{I}\text{-dppy})_6Cu^{I}_2](BF_4)_4$ (**2***, triangular prism) were synthesized according to the literature procedures with modifications[1-3].

For $[(C)(Au^{I}$-**BIPy**$)_6Cu^{I}_2](BF_4)_4$ (**1**): $[(C)(Au^{I}$-**BIPy**$)_6](BF_4)_2$ (27.9 mg, 10 μmol) was dissolved in dry CH_2Cl_2/CH_3OH (5.0 mL; v:v = 9:1) and $[Cu(CH_3CN)_4](BF_4)$ (9.4 mg, 30 μmol) was added to the solution with stirring. The reaction mixture was then filtered and the filtrate was overlaid with dry Et_2O to obtain yellow crystals of **1** in two weeks. Yield: 21.9 mg (71%, based on $[(C)(Au^{I}$-**BIPy**$)_6](BF_4)_2$).

Anal. Calcd for $C_{91}H_{90}N_{18}B_4F_{16}Cu_2Au_6\cdot 2C_4H_{10}O$: C, 36.70; H, 3.42; N, 7.78. Found: C, 36.84; H, 3.61; N, 7.98. ^{1}H NMR (500 MHz, CD_2Cl_2, 300 K, ppm): δ 8.13 (t, *J* = 7.7 Hz, 6H), 7.85 (d, *J*

= 8.3 Hz, 6H), 7.77 (d, J = 7.9 Hz, 6H), 7.70 (t, J = 7.8 Hz, 6H), 7.62 (d, J = 7.7 Hz, 12H), 6.29 (t, J = 6.2 Hz, 6H), 5.27 (t, J = 6.6 Hz, 6H), 1.41 (d, J = 6.7 Hz, 18H), 1.31 (d, J = 6.2 Hz, 18H). ^{13}C NMR (126 MHz, CD_2Cl_2, 300 K, ppm): δ 183.7, 151.6, 149.9, 143.3, 134.5, 132.0, 127.31, 127.26, 125.8, 124.9, 113.9, 112.9, 54.4, 22.6, 22.0. MS (ESI-TOF, positive-mode, solvent: CH_2Cl_2) m/z calcd. for $[(C)(Au\text{-}\mathbf{BIPy})_6]^{2+}$: 1308.8; found: 1308.8. Photoluminescence quantum yield (rt): 0.05 in CH_2Cl_2. Photoluminescence lifetime (rt): 1.15 μs in CH_2Cl_2.

For $[(C)(Au^I\text{-dppy})_6Cu^I_2](BF_4)_4$ (**2**, octahedral): $[(C)(Au^I\text{-dppy})_6](BF_4)_2$ (29.5 mg, 10 μmol) was dissolved in dry CH_2Cl_2/CH_3OH (5.0 mL; v:v = 9:1), and $[Cu(CH_3CN)_4](BF_4)$ (9.4 mg, 30 μmol) was added to the solution with stirring. The reaction mixture was then filtered and the filtrate was overlaid with dry Et_2O to obtain yellow crystals of **2** in one week. Yield: 27.6 mg (85%, based on $[(C)(Au^I\text{-dppy})_6](BF_4)_2$).

Anal. Calcd for $C_{103}H_{84}N_6B_4F_{16}P_6Cu_2Au_6 \cdot 4CH_2Cl_2$: C, 35.82; H, 2.58; N, 2.34. Found: C, 35.64; H, 2.82; N, 2.57. ^{1}H NMR (500 MHz, CD_2Cl_2/CD_3OD = 9:1 (v:v), 300 K, ppm): δ 7.88-7.78 (m, 6H), 7.62-7.41 (m, 42H), 7.41-7.10 (m, 30H), 7.05-6.87 (m, 6H). ^{31}P NMR (202 MHz, CD_2Cl_2/CD_3OD = 9:1 (v:v), 300 K, ppm): δ 30.40. ^{13}C NMR (125 MHz, CD_2Cl_2/CD_3OD = 9:1 (v:v), 300 K, ppm): δ 154.0, 153.3, 153.2, 153.1, 140.4, 134.6, 134.0, 133.1, 132.2, 132.1, 130.7, 130.6, 130.1, 130.0, 128.6. MS (ESI-TOF, positive-mode, solvent: CH_2Cl_2) m/z calcd. for $[(C)(Au\text{-dppy})_6Cu](BF_4)^{2+}$: 1461.6; found: 1461.7. Photoluminescence quantum yield (rt): 0.15 in CH_2Cl_2. Photoluminescence lifetime (rt): 3.54 μs in CH_2Cl_2.

For $[(C)(Au^I\text{-dppy})_6Cu^I_2](BF_4)_4$ (**2***, triangular prism): $[(C)(Au^I\text{-dppy})_6](BF_4)_2$ (29.5 mg, 10 μmol) was dissolved in dry CH_2Cl_2/CH_3OH (5.0 mL; v:v = 9:1), and $[Cu(CH_3CN)_4](BF_4)$ (9.4 mg, 30 μmol) was added to the solution with stirring. The reaction mixture was then filtered and n-C_6H_{14} (0.5 mL) was added to the filtrate. The reaction mixture was allowed to evaporate at 4 °C, giving red crystals of **2*** in a week. Yield: 25.0 mg (77%, based on $[(C)(Au\text{-dppy})_6](BF_4)_2$). It should be noted that the only difference between the synthetic routes of **2*** and **2** is the crystallization process. However, crystallization of octahedral **1**, **3**, and **4** under the conditions for crystallization of triangular prism **2*** gives only octahedral products.

^{1}H NMR (500 MHz, CD_2Cl_2/CD_3OD = 9:1 (v:v), 300 K, ppm): δ 7.82-7.72 (m, 6H), 7.60-7.40 (m, 42H), 7.40-7.06 (m, 30H), 7.02-6.80 (m, 6H). ^{31}P NMR (202 MHz, CD_2Cl_2/CD_3OD = 9:1 (v:v), 300 K, ppm): δ 30.40. ^{13}C NMR (125 MHz, CD_2Cl_2/CD_3OD = 9:1 (v:v), 300 K, ppm): δ 154.0, 153.3, 153.1, 140.4, 134.5, 134.0, 133.1, 132.2, 132.1, 130.7, 130.6, 130.1, 130.0, 128.6. MS (ESI-TOF, positive-mode, solvent: CH_2Cl_2) m/z calcd. for $[(C)(Au\text{-dppy})_6Cu](BF_4)^{2+}$: 1461.6; found: 1461.7. Photoluminescence quantum yield (rt): 0.15 in CH_2Cl_2. Photoluminescence lifetime (rt): 3.44 μs in CH_2Cl_2.

4. X-ray crystallography

Intensity data for compounds $[(C)(Au^I\text{-}\mathbf{BIPy})_6Cu^I_2](BF_4)_4$ (**1**) and $[(C)(Au^I\text{-dppy})_6Cu^I_2](BF_4)_4$ (**2**, octahedral) were collected at 93 K on a Rigaku XtaLAB Synergy-DW system (CuKα). The structures were solved by direct methods, and non-hydrogen atoms, except for the central carbon atom in **2**, were anisotropically refined by the least-squares on F^2 using the SHELXTL program. The hydrogen atoms of the organic ligands were geometrically generated.

For $[(C)(Au^{I}$-**BIPy**$)_6Cu^{I}_2](BF_4)_4$ (**1**): $C_{91}H_{90}N_{18}B_4F_{16}Cu_2Au_6$, $a = b = c$ = 21.3163(2) Å, V = 9685.8(3) Å^3, triclinic space group Pa-3, Z = 4, T = 93(2) K, 55904 reflections measured, 3258 unique (R_{int} = 0.0482), final R_1 = 0.1065, wR_2 = 0.3894 for 1813 observed reflections [$I > 2\sigma(I)$].

For $[(C)(Au^{I}\text{-dppy})_6Cu^{I}_2](BF_4)_4$ (**2**, octahedral): $C_{103}H_{84}N_6B_4F_{16}P_6Cu_2Au_6 \cdot 2CH_2Cl_2$, a = 15.7328(3), b = 18.9651(4), c = 18.0783(4) Å, β = 91.597(2)°, V = 5392.00(19) Å^3, triclinic space group $P2_1/n$, Z = 2, T = 93(2) K, 45601 reflections measured, 9412 unique (R_{int} = 0.0910), final R_1 = 0.0466, wR_2 = 0.1172 for 8839 observed reflections [$I > 2\sigma(I)$].

5. X-ray absorption spectroscopy

Au L_3-edge X-ray absorption fine structure (XAFS) spectra of the samples were recorded at the BL01B1 beamline of the SPring-8 facility of the Japan Synchrotron Radiation Research Institute. Si(111) double-crystal monochromators were used to obtain the incident X-ray beam. Solid samples were mixed with BN, pressed into a pellet and mounted on a copper holder attached to the cryostat. The XAFS spectra of solid samples were measured in the transmission mode using ionization chambers at 10, 173 and 300 K. The samples dissolved in CH_2Cl_2 and CH_3OH solutions were located in the Teflon cell, and their XAFS spectra were measured in the fluorescence mode using an ionization chamber and 19-element Ge solid state detectors at 173 and 300 K. The temperature of each solution sample was controlled by a liquid N_2 spray apparatus. The XAFS data were analyzed by xTunes software.[4] After normalization of XAFS spectra, k^3-weighted EXAFS spectra in the ranges of 3-18 Å^{-1} for the samples measured at 10 K and 3-13 Å^{-1} for the samples at 173 and 300 K were Fourier transformed into r space. The curve fitting analysis was conducted in the r range of 1.5-3.0 Å using fixed ΔE_0. The phase shifts and backscattering amplitude functions for Au-C, Au-P, Au-Cu, Au-Ag, and Au-Au were calculated by FEFF8.5L.[5]

6. Computation details

All metal clusters were optimized using the B3LYP functional.[6] The relativistic effective core potential LANL2DZ,[7] was used for Au, Ag, and Cu atoms, and the basis set for the other atoms (C, N, P, and H) was 6-31G*.[8-12] The optimization was performed based on the crystal structures, and vibrational frequency analysis was conducted to verify that the stationary points were the local minima on the potential energy surface. In the simulation of the absorption spectra, 100 excited states were analyzed to cover the spectrum in the energy range up to about 250 nm. TD-DFT calculations were performed using the MN15 functional,[13] including the solvent effect of CH_2Cl_2 using the polarizable continuum model (PCM) and a non-equilibrium linear response scheme.[14] The information of the singly excited configuration was obtained using cclib.[15] To calculate the phosphorescence energies, the B3LYP functional was used to obtain the optimal geometry of the lowest triplet excited state (T_1), and the emission energy was calculated in this structure using the Δ self-consistent-field (ΔSCF) approach. The ωB97XD[16] and M06-2X[17] functionals were also considered to investigate the functional dependence of the stability of all metal clusters. The solvent effects of CH_2Cl_2, n-hexane, diethyl ether, and mixed solvents (CH_2Cl_2/n-hexane, v = 1:1; CH_2Cl_2/diethyl ether, v = 1:1) were calculated based on the solvent excluded surface (SES) using the polarizable continuum model (PCM). For the mixed solvents, the dielectric constant of each solvent was calculated as a weighted average based on the proportion of each solvent component

(volume ratio of each component), and a custom solvent profile was created. Wiberg bond orders were calculated at the ωB97XD/6-31G*~LANL2DZ level of theory using NBO 3.1[18] as implemented in Gaussian 16. All calculations were conducted using the Gaussian 16 program suite[19].

7. Supporting schemes, figures, and tables

Scheme S1. Synthesis and crystallization of clusters **1-4** and **2***.

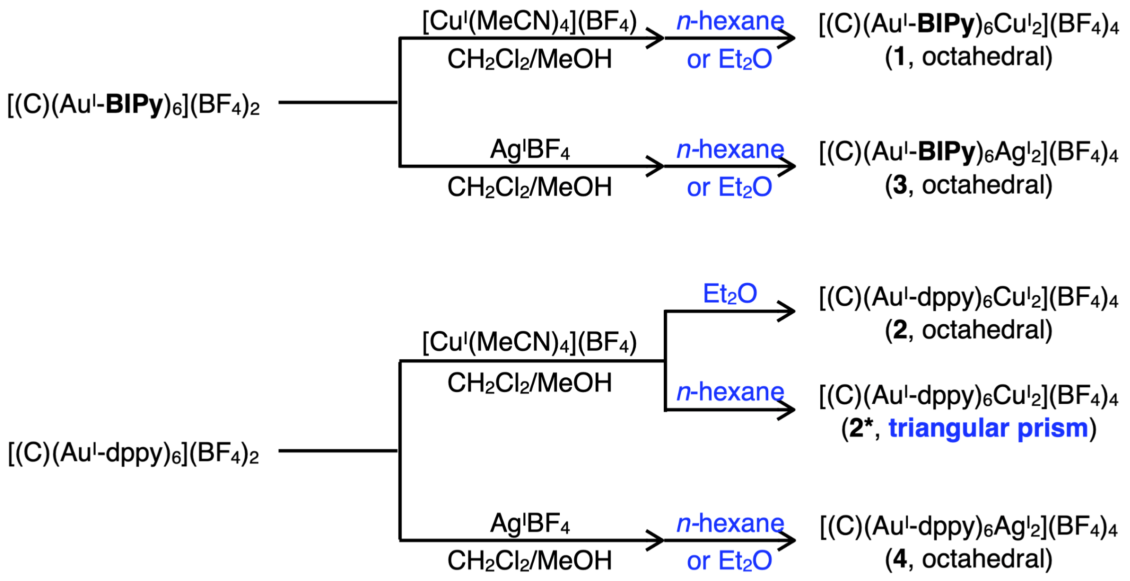

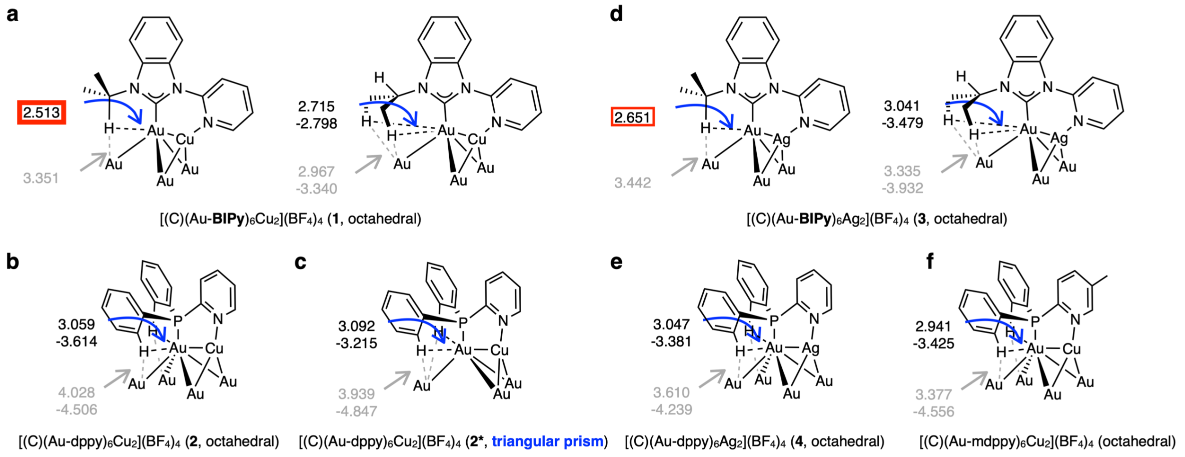

Figure S1. Schematic illustration of intramolecular Au···H−C interactions in **1-4** and **2***.

Table S1. Key structural parameters of **1-4** and **2***, and related $CAu^I_6M^I_2$ clusters (M = Ag, Cu).

	**Au–C (central C^{4-}, Å)**	**Au–Au (Å)**	**Au–C (NHC ligands) or Au–P (Å)**	**Au–M (Å, M = Ag, Cu)**	**M–N (Å, M = Ag, Cu)**
$[(C)(Au^I$-**BIPy**$)_6Cu^I_2](BF_4)_4$ (**1**, *octa.*)	2.1022(9)	2.9719(16)-2.9739(16)	2.04(2)	2.772(5)	2.184(16)
$[(C)(Au^I$-dppy$)_6Cu_2](BF_4)_4$ (**2**, *octa.*)	2.0972(3)-2.1095(3)	2.9153(4)-3.0422(4)	2.248(2)-2.253(2)	2.8145(9)-2.9084(9)	2.104(7)-2.151(6)
$[(C)(Au^I$-mdppy$)_6Cu^I_2](BF_4)_6$ (*octa.*)[3]	2.1076(2)-2.1219(2)	2.9207(4)-3.0741(3)	2.2639(17)-2.2703(17)	2.8567(8)-2.9712(7)	2.127(6)-2.206(6)
$[(C)(Au^I$-**BIPy**$)_6Ag^I_2](BF_4)_4$ (**3**, *octa.*)[1]	2.1100(6)	2.9474(10)-3.0200(11)	2.00(2)	2.8467(17)	2.337(10)-2.34(2)
$[(C)(Au^I$-**BIMPy**$)_6Ag^I_2](BF_4)_4$ (*octa.*)[1]	2.1102(4)-2.1185(4)	2.9471(5)-3.0252(5)	2.025(10)-2.027(10)	2.8372(8)-2.8729(8)	2.393(8)-2.402(9)
$[(C)(Au^I$-dppy$)_6Ag^I_2](BF_4)_4$ (**4**, *octa.*)[2]	2.0980(4)-2.1144(4)	2.9406(5)-3.0164(5)	2.250(2)-2.266 (2)	2.9134(8)-2.9316 (8)	2.345(8)-2.356(8)
$[(C)(Au^I$-dppy$)_6Cu_2](BF_4)_4$ (**2***, *triangular prism*)[3]	2.137(13)-2.168(7)	2.7529(6)-2.8672(7)	2.252(3)–2.256(3)	2.8550(14)-2.9074(15)	2.095(11)-2.117(9)

(mdppy = 2-(5-methylpyridyl)diphenylphosphine, **BIMPy** = *N*-isopropyl-*N'*-2-(5-methylpyridyl)benzimidazolylidene)

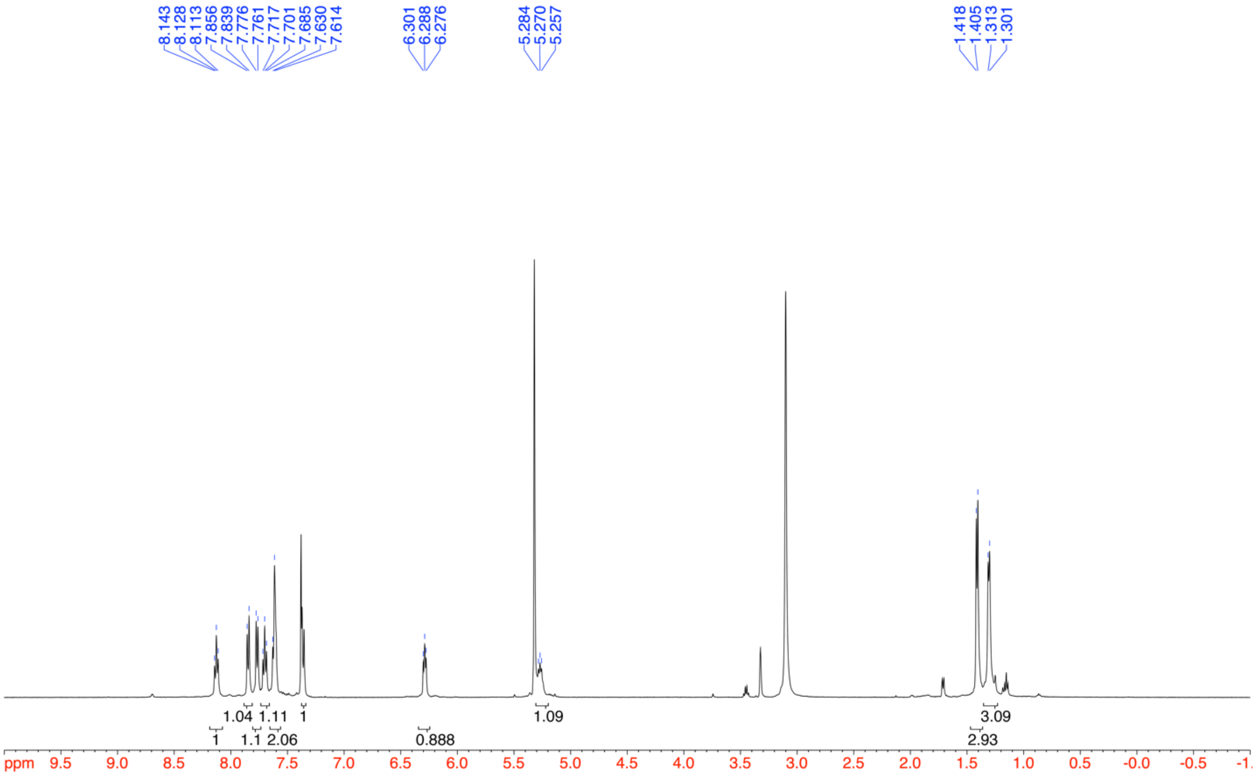

Figure S2. ^1H NMR spectrum of **1** (500 MHz, CD_2Cl_2/CD_3OD (9:1, v:v), 300 K).

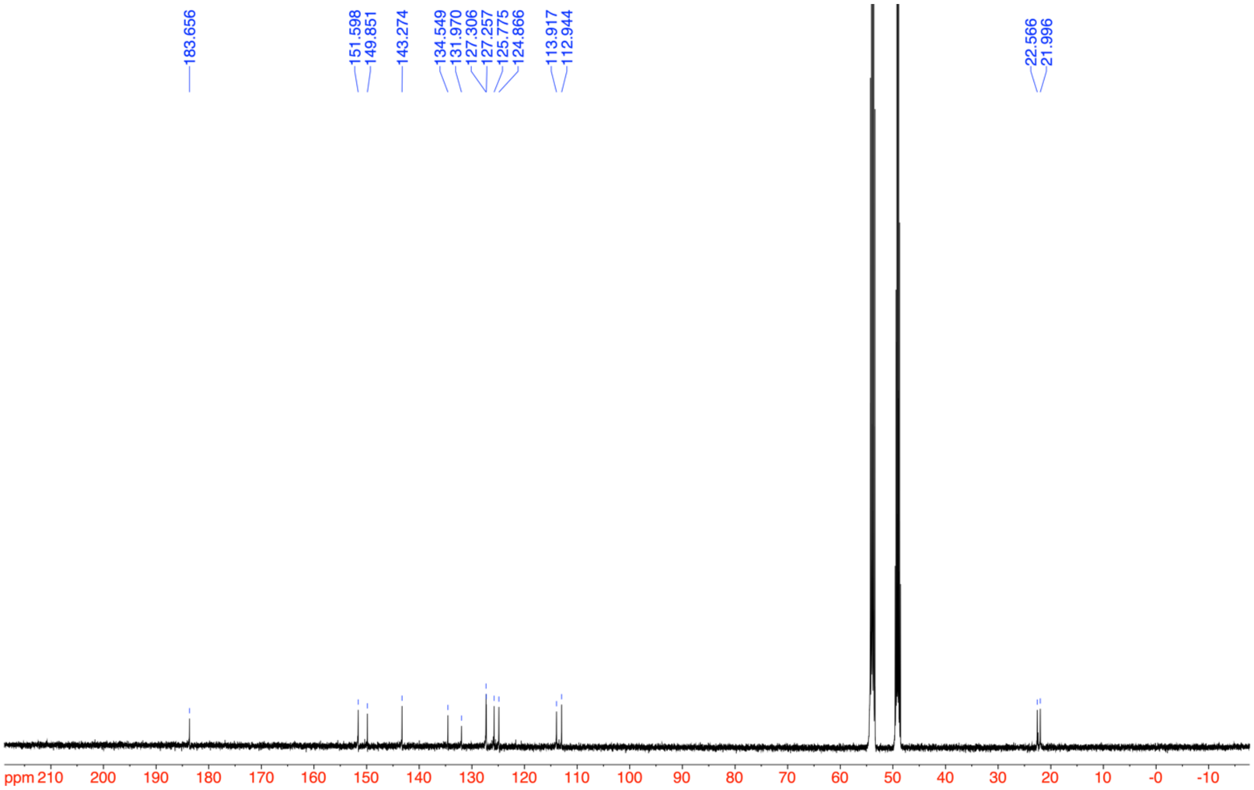

Figure S3. ^{13}C NMR spectrum of **1** (125 MHz, CD_2Cl_2/CD_3OD (9:1, v:v), 300 K).

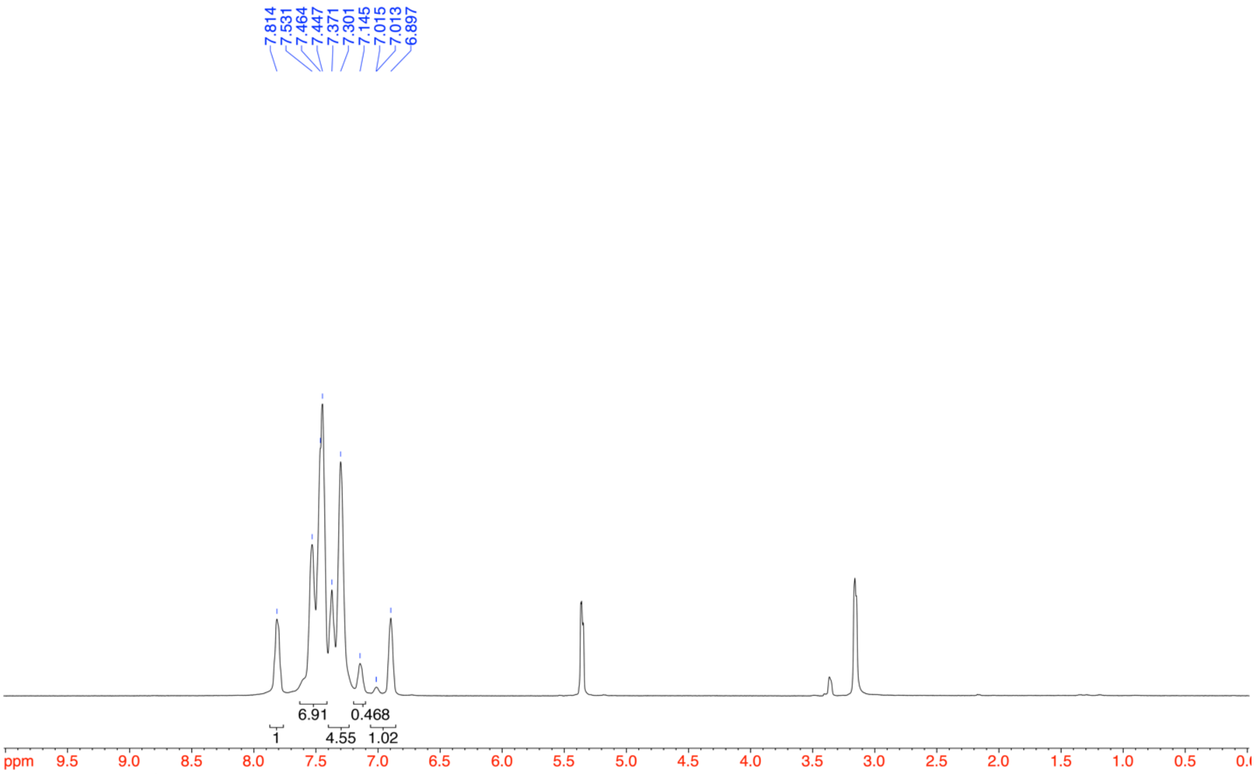

Figure S4. ^{1}H NMR spectrum of **2** (500 MHz, CD_2Cl_2/CD_3OD (9:1, v:v), 300 K).

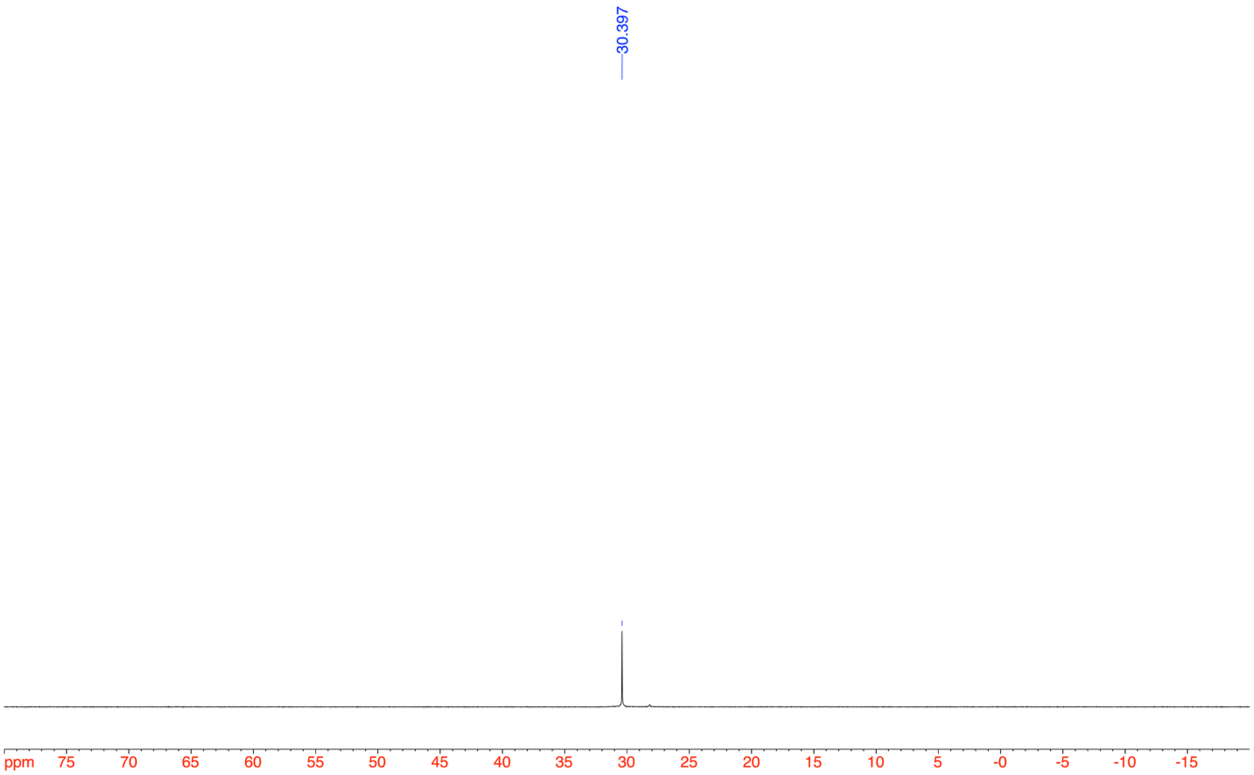

Figure S5. ^{31}P NMR spectrum of **2** (202 MHz, CD_2Cl_2/CD_3OD (9:1, v:v), 300 K).

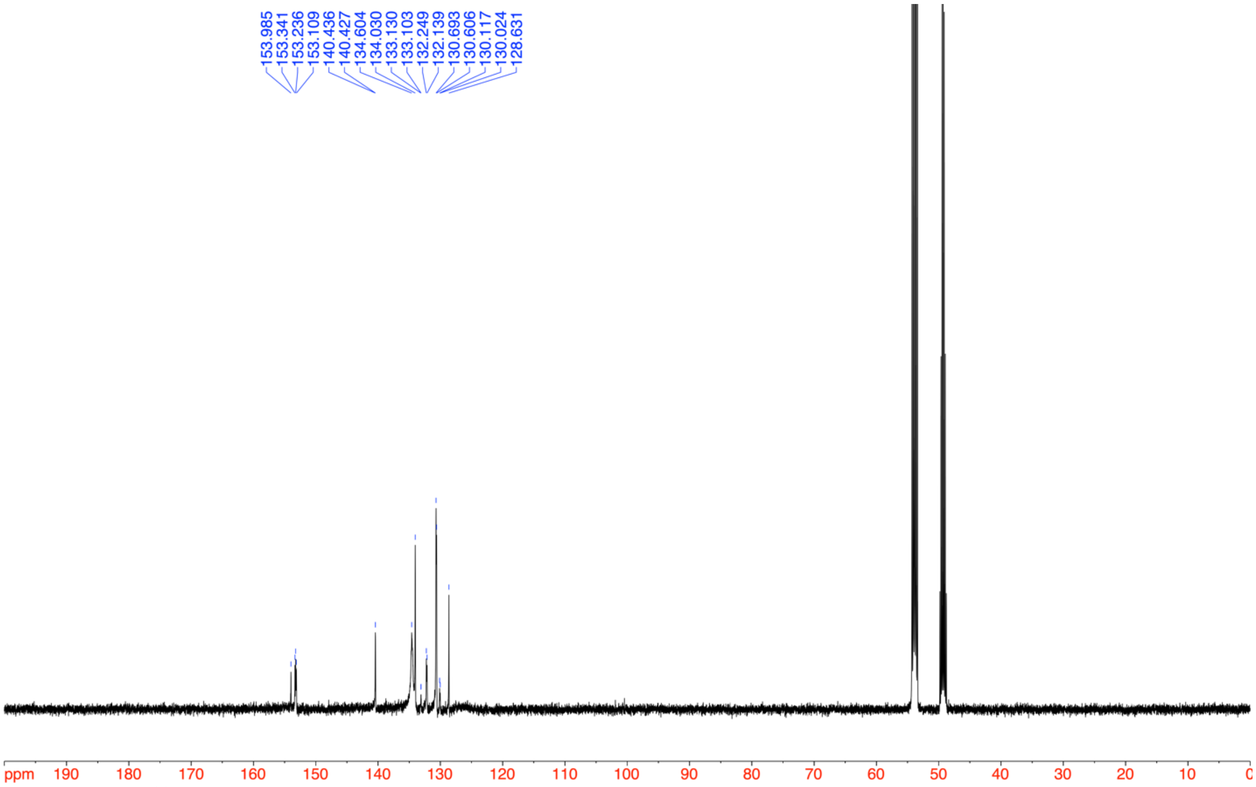

Figure S6. ^{13}C NMR spectrum of **2** (125 MHz, CD_2Cl_2/CD_3OD (9:1, v:v), 300 K).

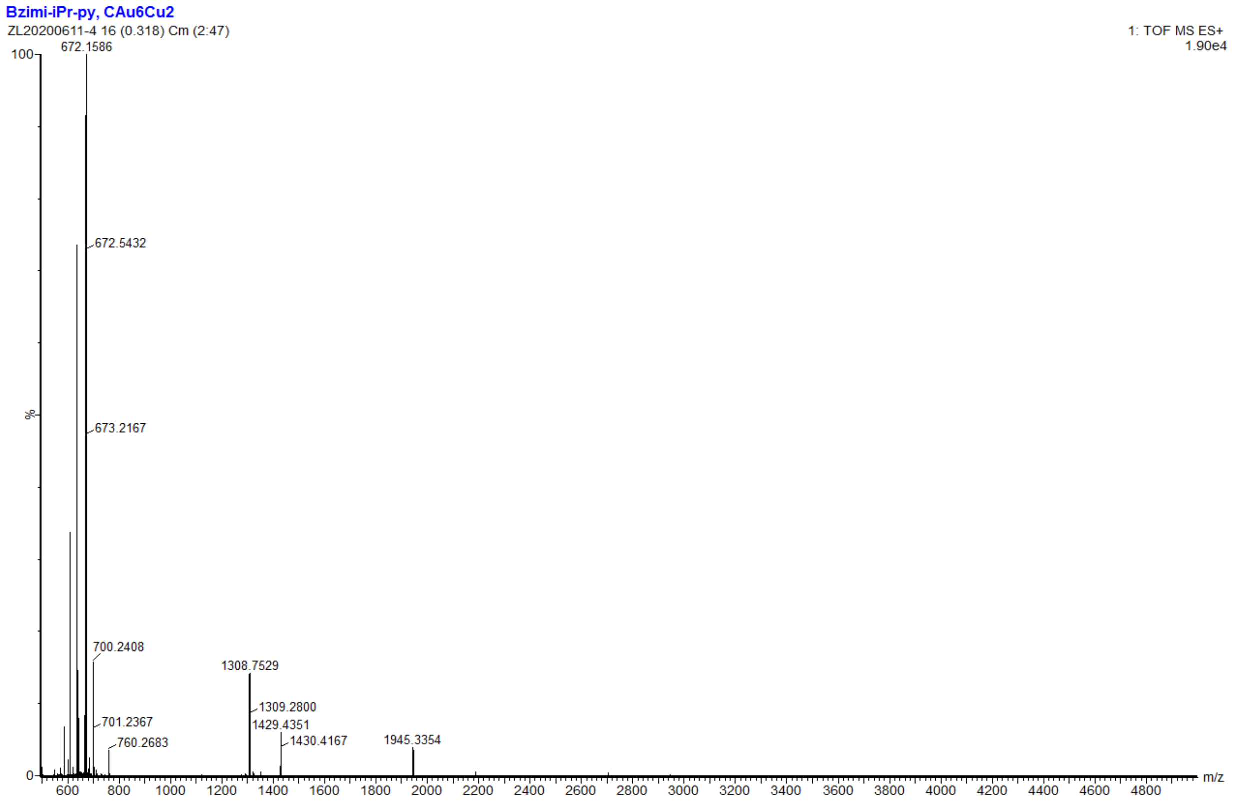

Figure S7. ESI MS spectrum of **1** in CH_2Cl_2.

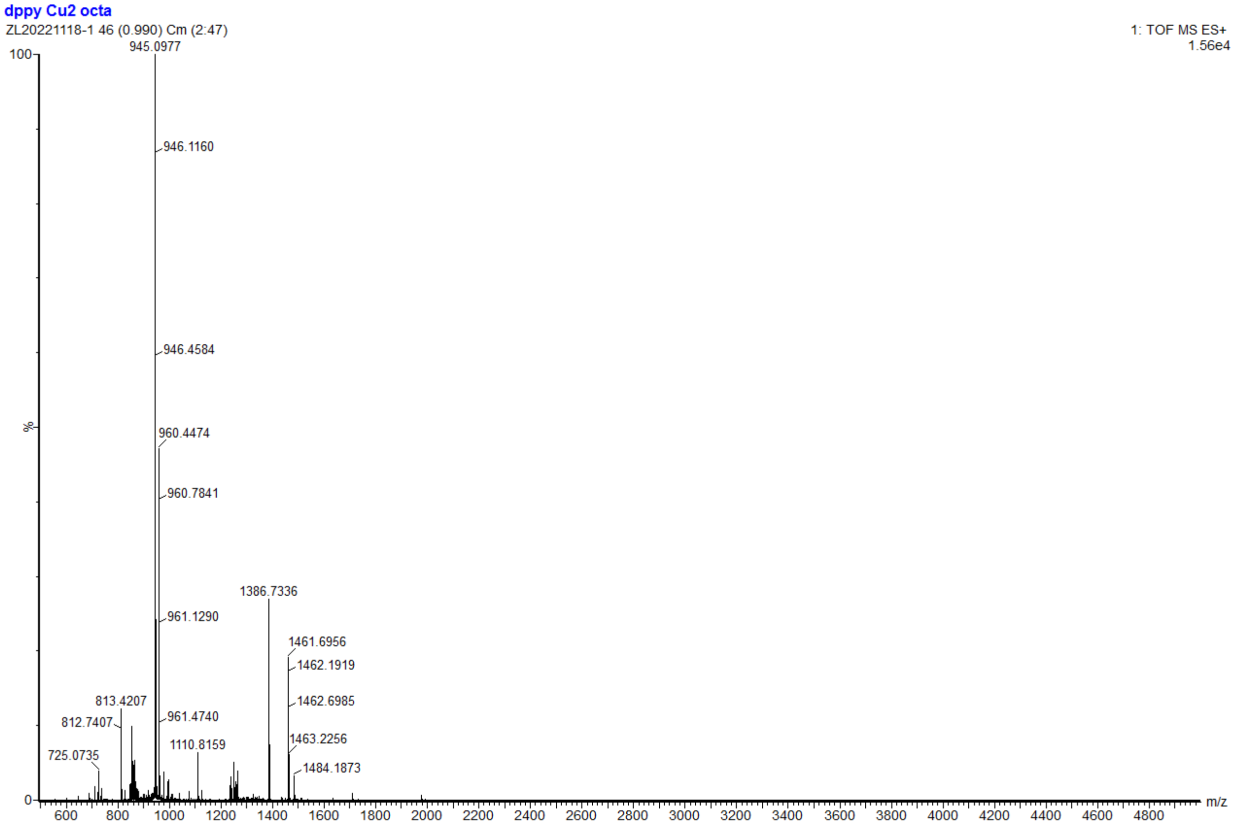

Figure S8. ESI MS spectrum of **2** in CH_2Cl_2.

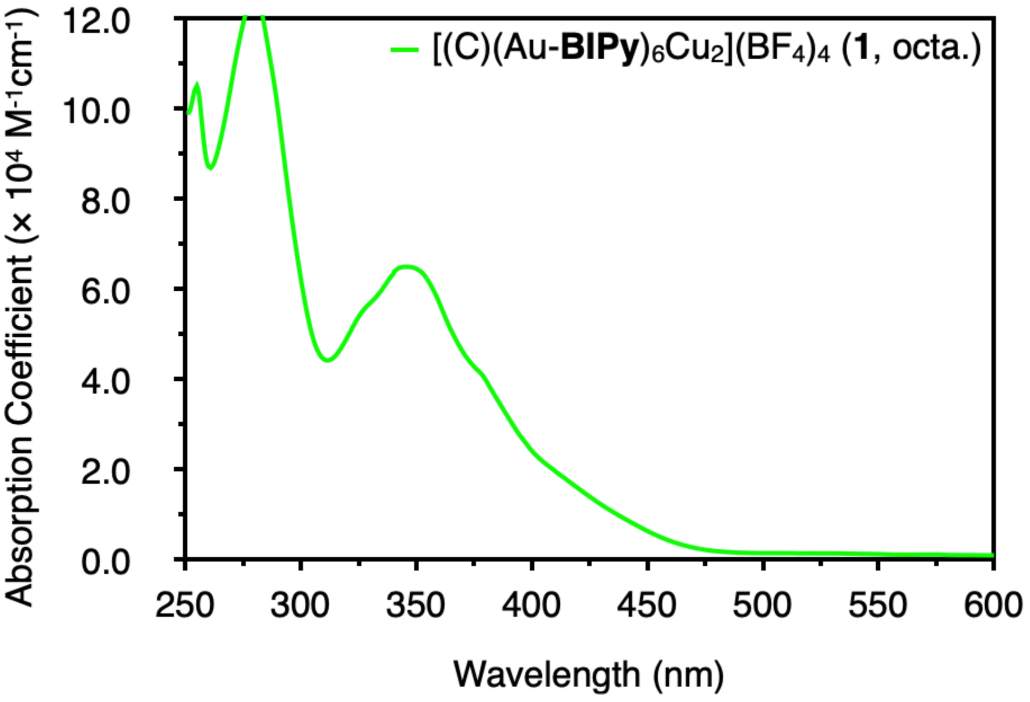

Figure S9. UV-vis absorption spectrum of **1** in CH_2Cl_2. (c = 1.0 × 10^{-6} mol/L, 300 K)

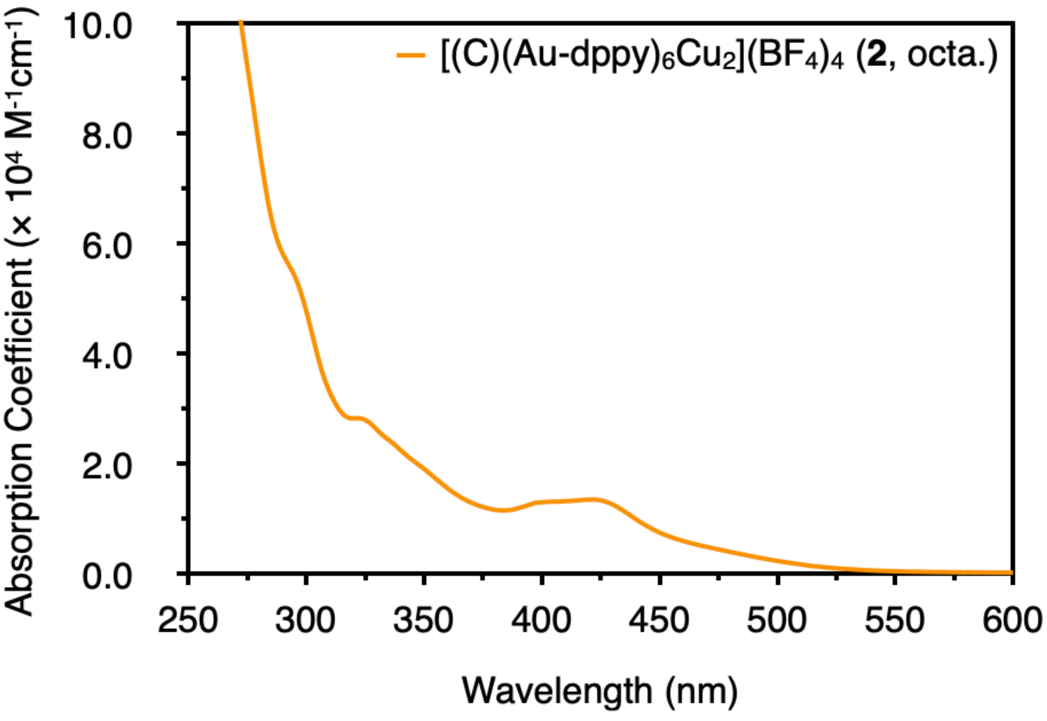

Figure S10. UV-vis absorption spectrum of **2** in CH_2Cl_2. (c = 1.0 × 10^{-6} mol/L, 300 K)

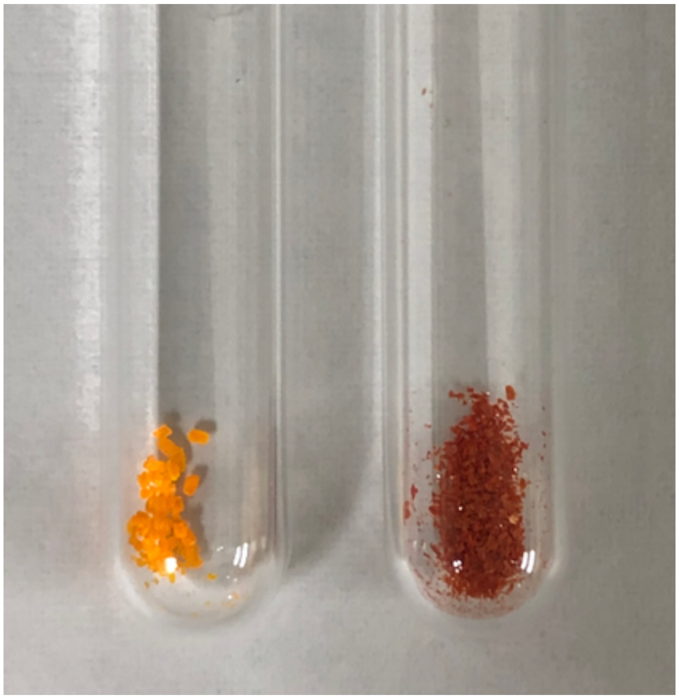

Figure S11. A photo of crystals of **2** (left) and **2*** (right) under sunlight.

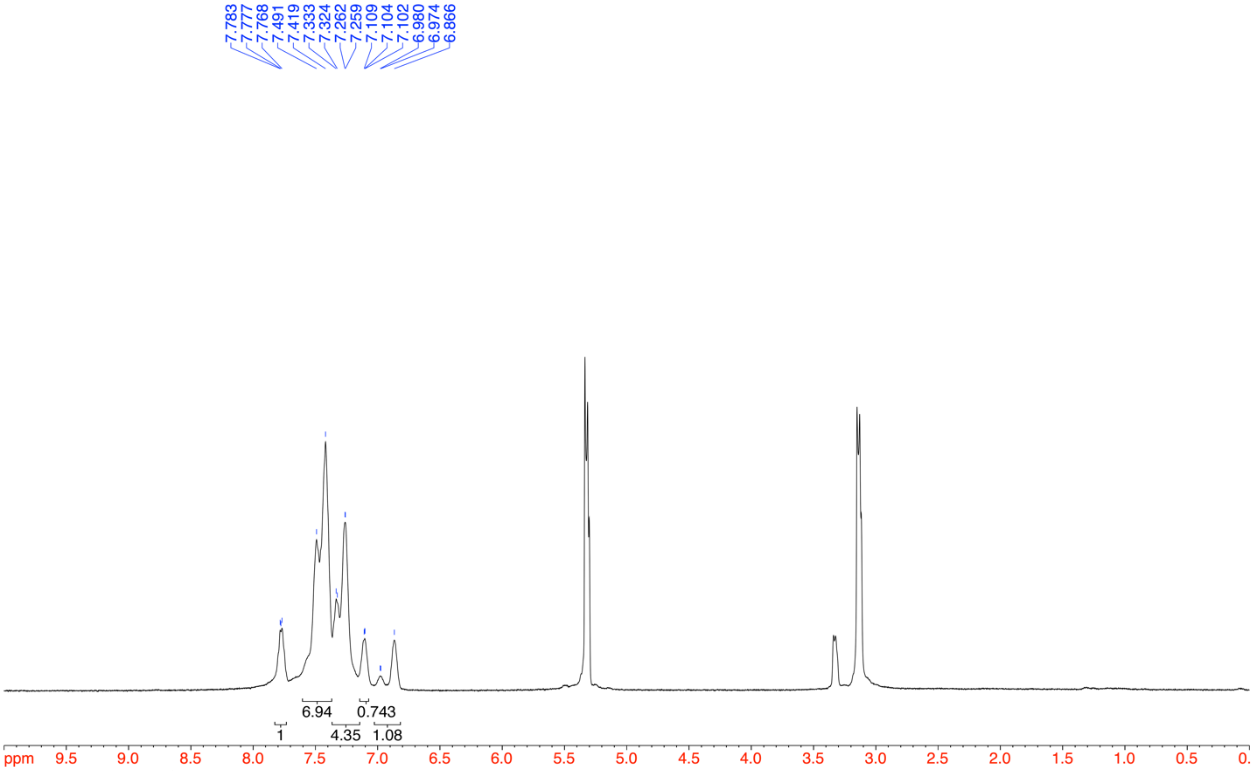

Figure S12. ^{1}H NMR spectrum of **2*** (500 MHz, CD_2Cl_2/CD_3OD (9:1, v:v), 300 K).

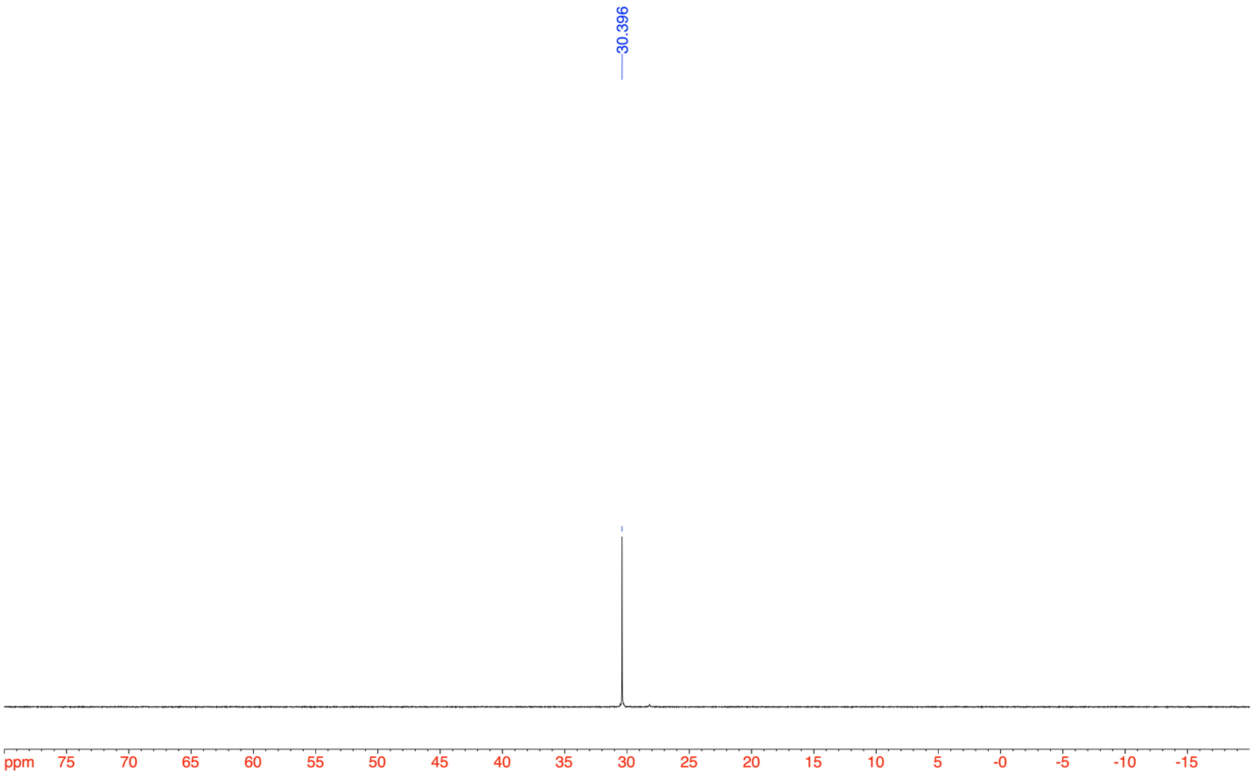

Figure S13. ^{31}P NMR spectrum of **2*** (202 MHz, CD_2Cl_2/CD_3OD (9:1, v:v), 300 K).

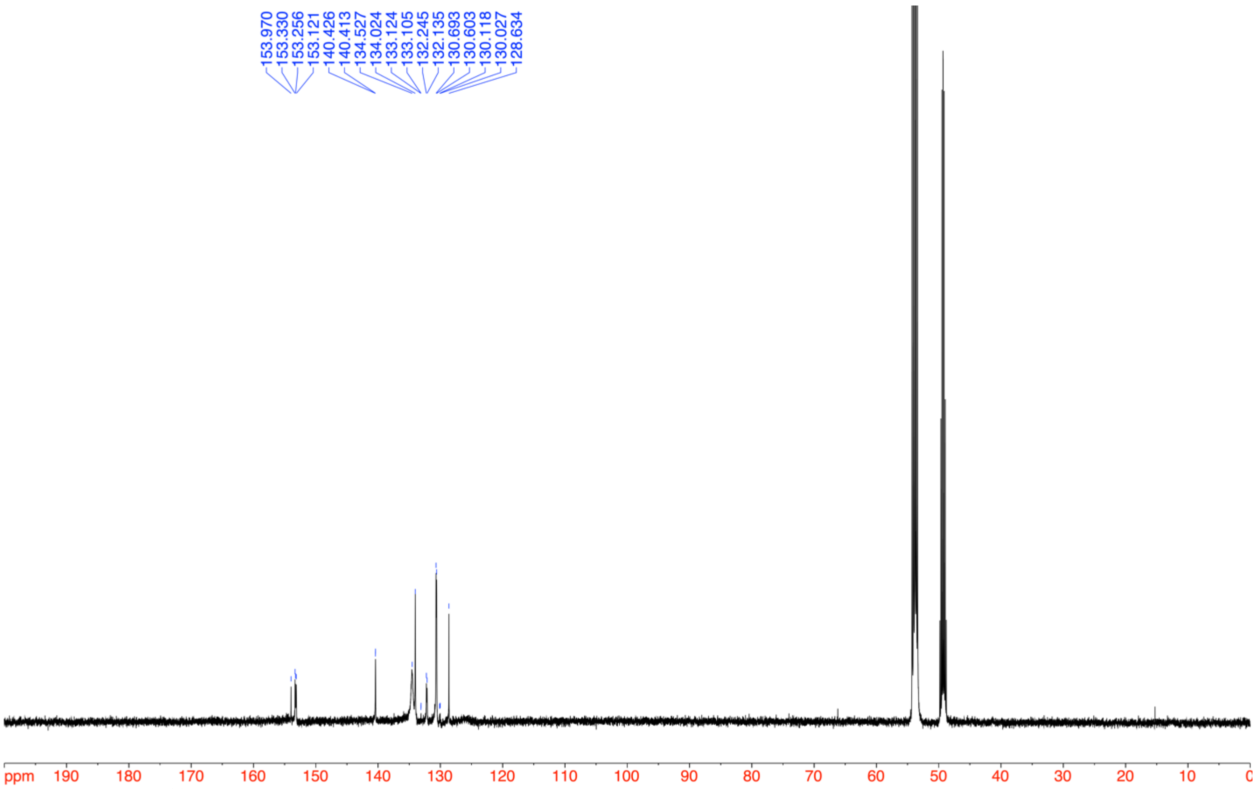

Figure S14. ^{13}C NMR spectrum of **2*** (125 MHz, CD_2Cl_2/CD_3OD (9:1, v:v), 300 K).

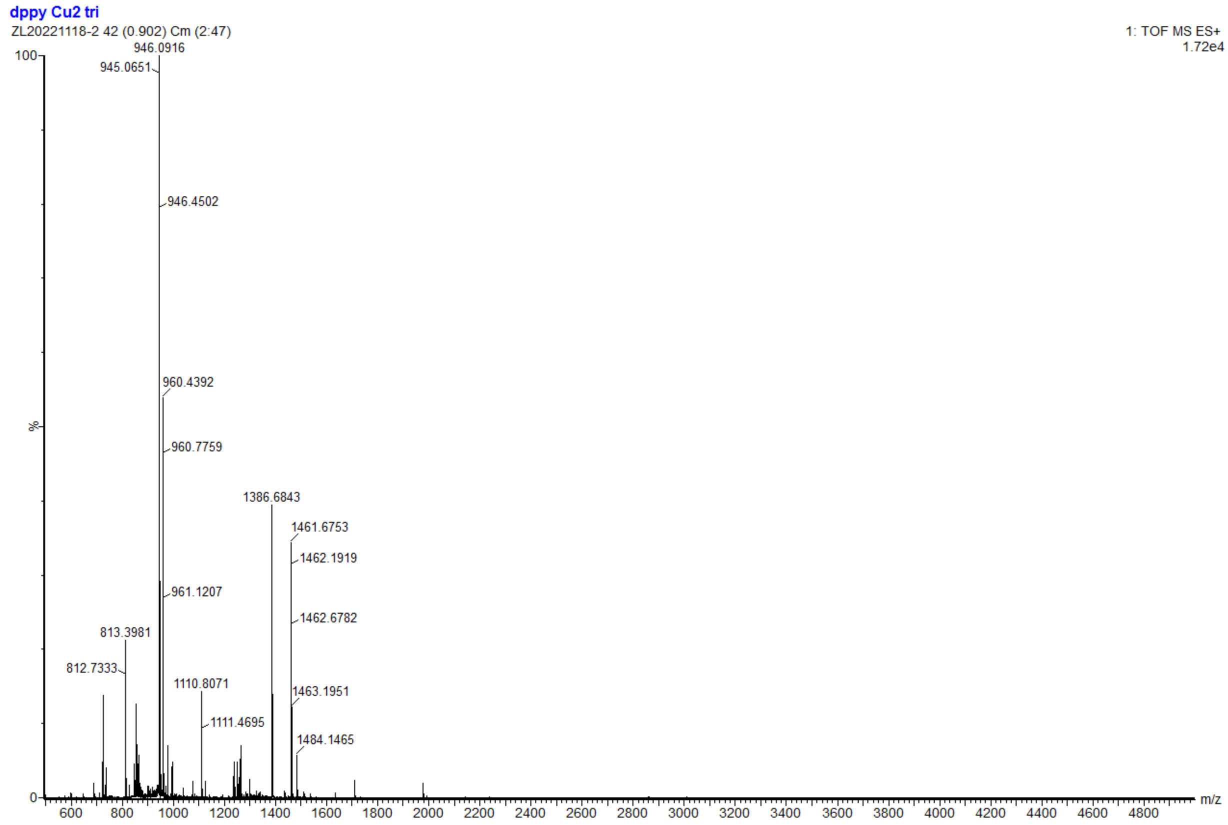

Figure S15. ESI MS spectrum of **2*** in CH_2Cl_2.

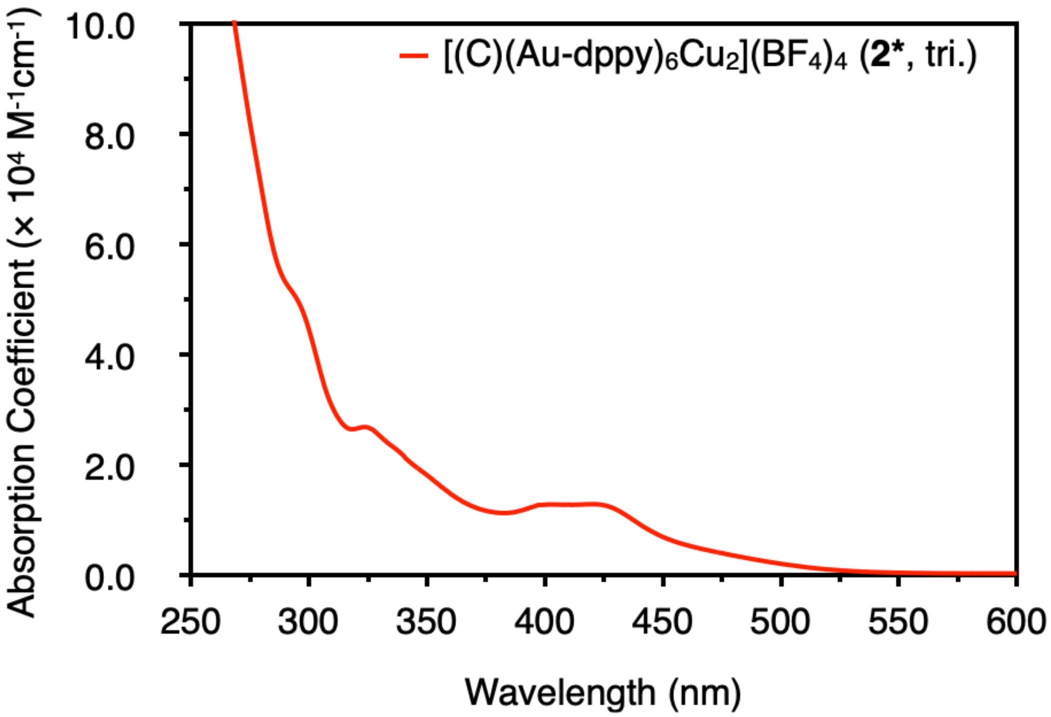

Figure S16. UV-vis absorption spectrum of **2*** in CH_2Cl_2. ($c = 1.0 \times 10^{-6}$ mol/L, 300 K)

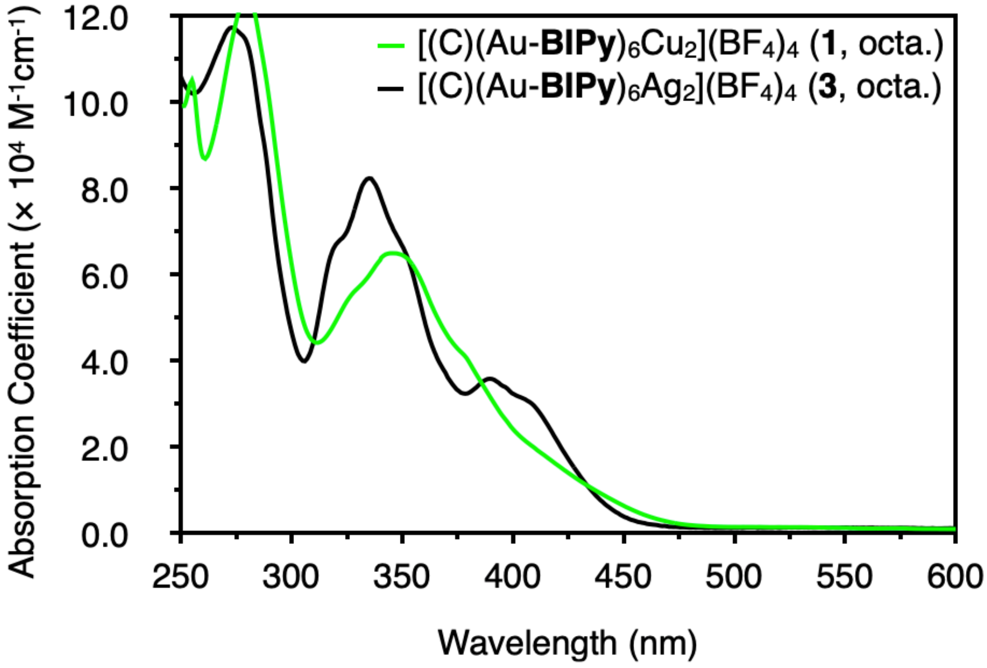

Figure S17. Comparison of UV-vis absorption spectra of **1** and **3** in CH_2Cl_2. ($c = 1.0 \times 10^{-6}$ mol/L, 300 K)

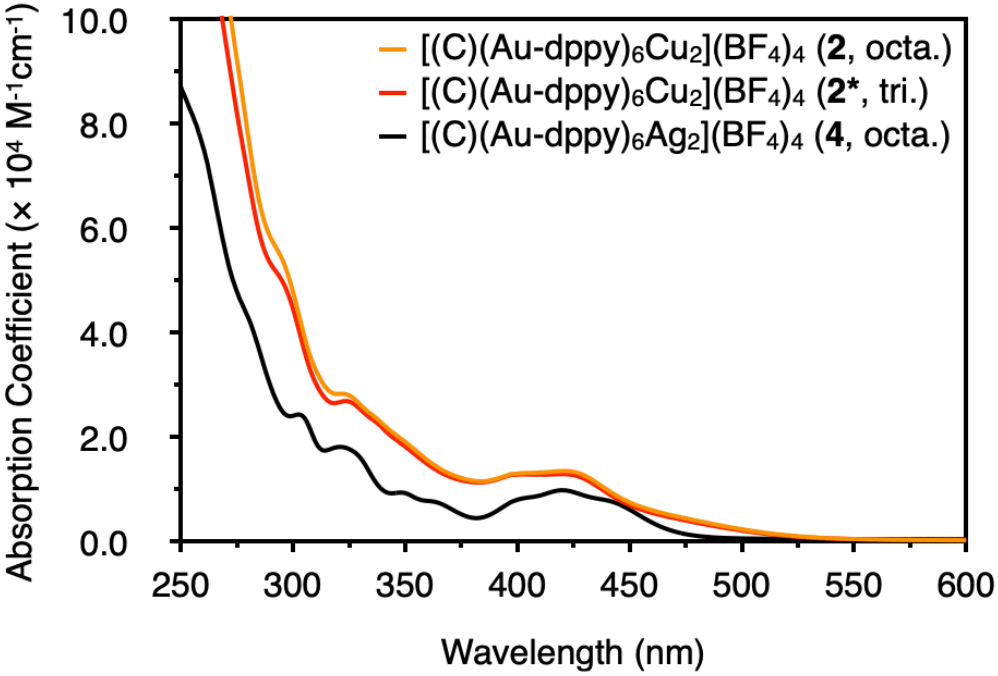

Figure S18. Comparison of UV-vis absorption spectra of **2**, **2*** and **4** in CH_2Cl_2. ($c = 1.0 \times 10^{-6}$ mol/L, 300 K)

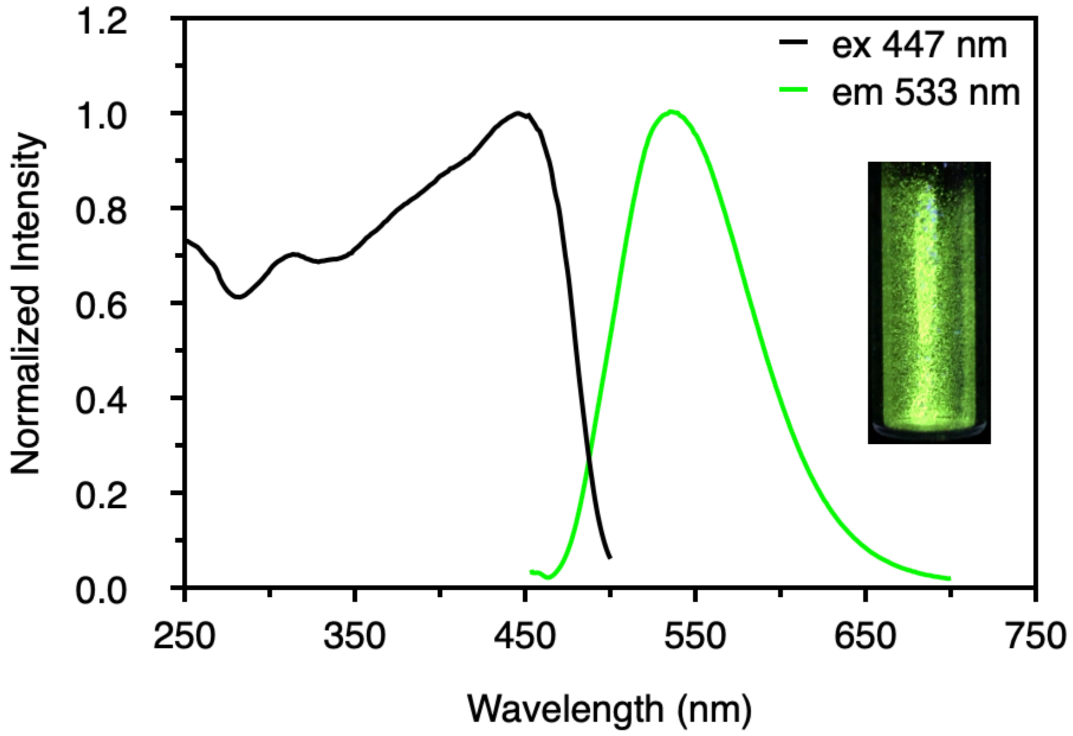

Figure S19. Excitation and photoluminescence spectra of **1** in the solid state at 300 K.

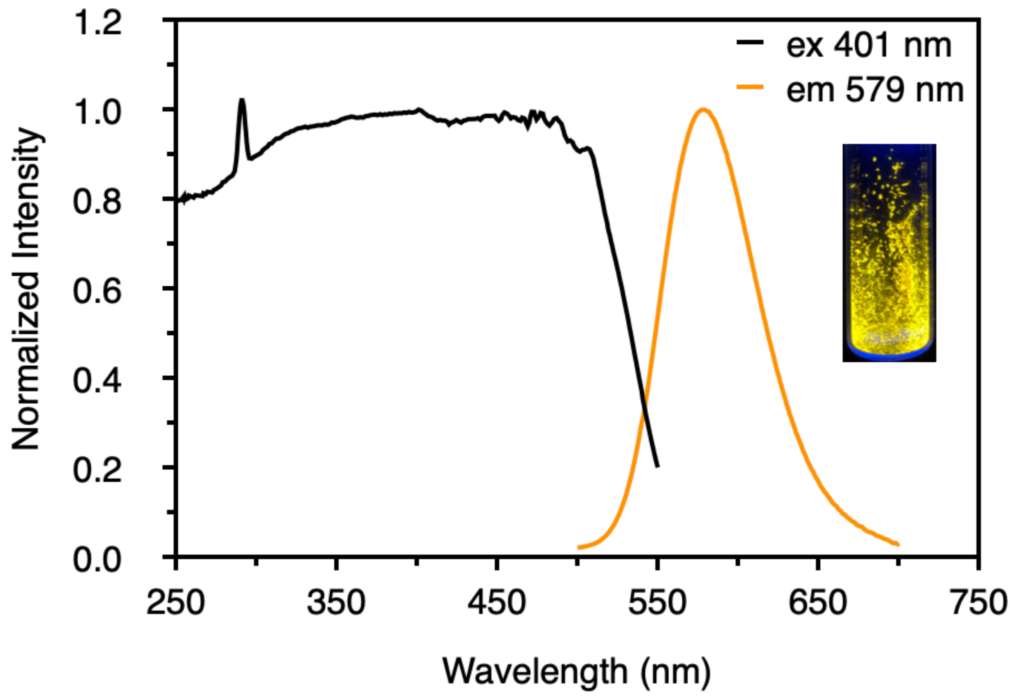

Figure S20. Excitation and photoluminescence spectra of **2** in the solid state at 300 K.

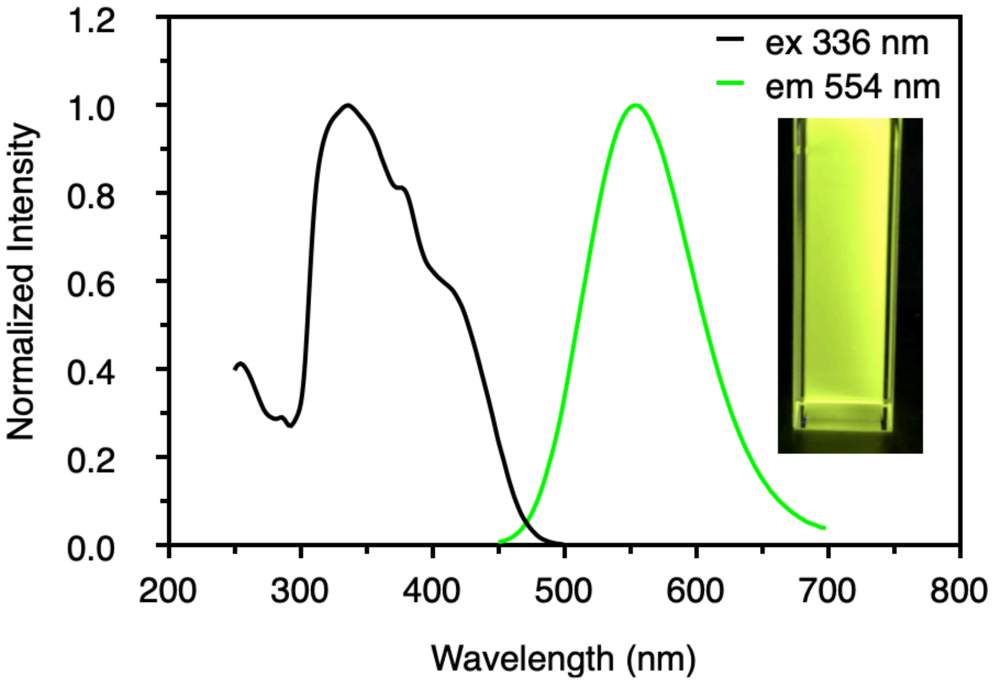

Figure S21. Excitation and photoluminescence spectra of **1** in CH_2Cl_2. ($c = 1.0 \times 10^{-6}$ mol/L, 300 K)

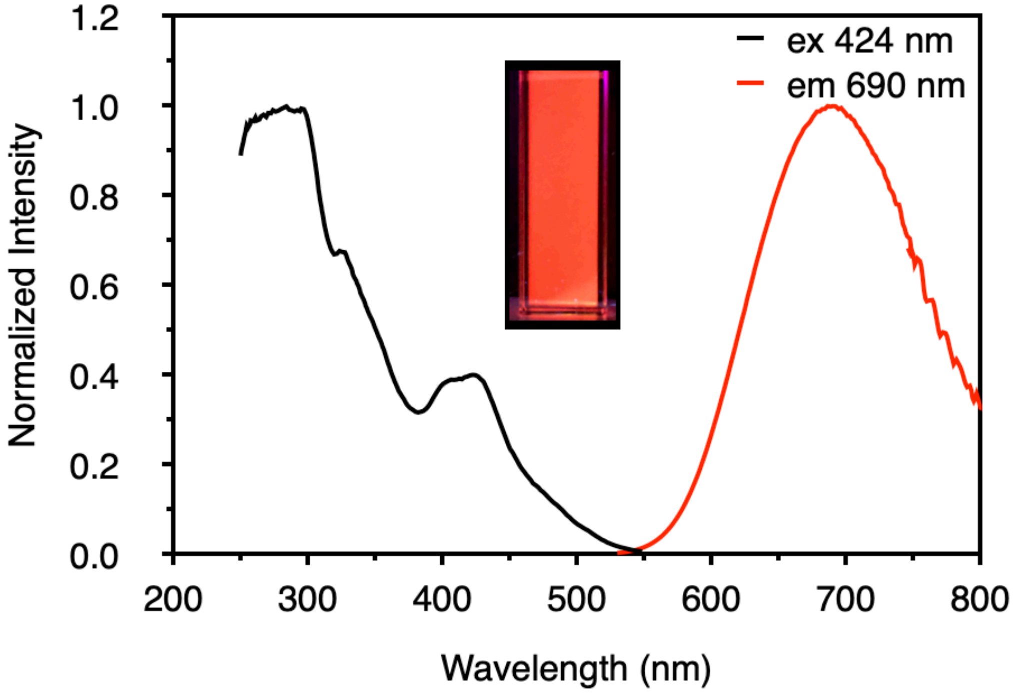

Figure S22. Excitation and photoluminescence spectra of **2** in CH_2Cl_2. ($c = 1.0 \times 10^{-6}$ mol/L, 300 K)

Table S2. Photoluminescence QYs (φ), lifetimes (τ), and radiative (k_r) and non-radiative (k_{nr}) rate constants of **1-4** and **2*** in CH_2Cl_2.

Cluster	φ	τ (μs)	k_r (× 10^5 s^{-1})	k_{nr} (× 10^5 s^{-1})
1	0.05	1.15	0.43	8.26
2	0.15	3.54	0.42	2.40
3	0.86	1.66	5.18	0.84
4	0.31	3.74	0.83	1.84
2*	0.15	3.44	0.44	2.47

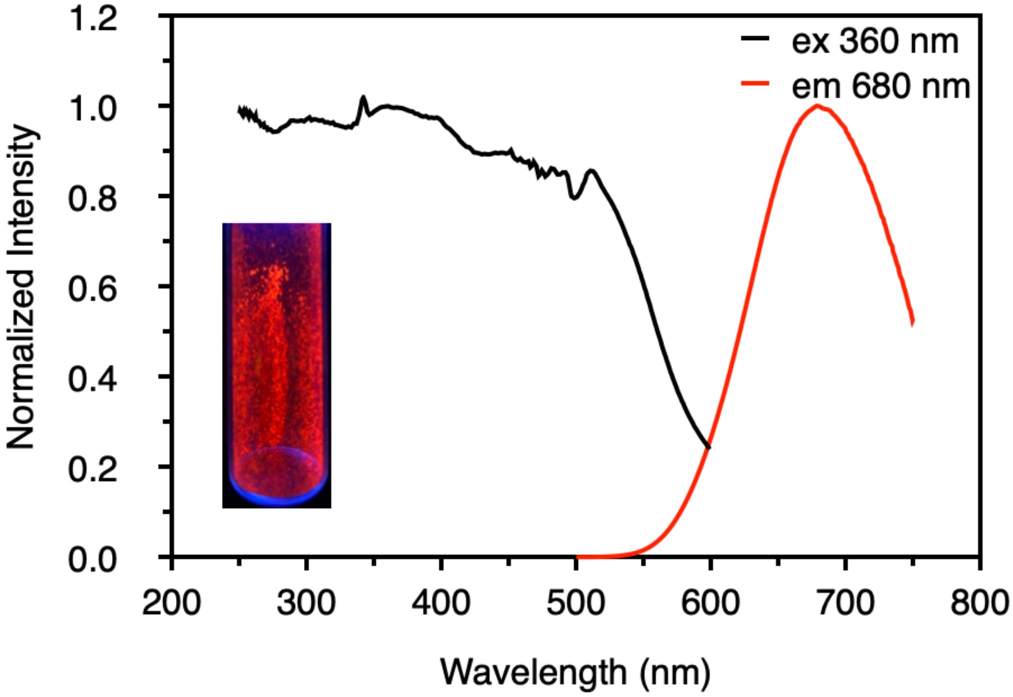

Figure S23. Excitation and photoluminescence spectra of **2*** in the solid state at 300 K.

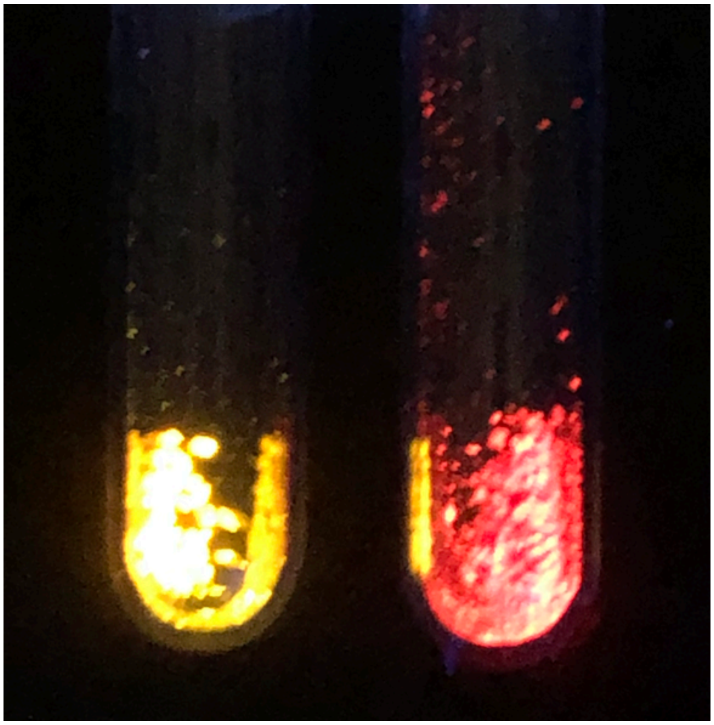

Figure S24. A photo of crystals of **2** (left) and **2*** (right) under 365 nm excitation.

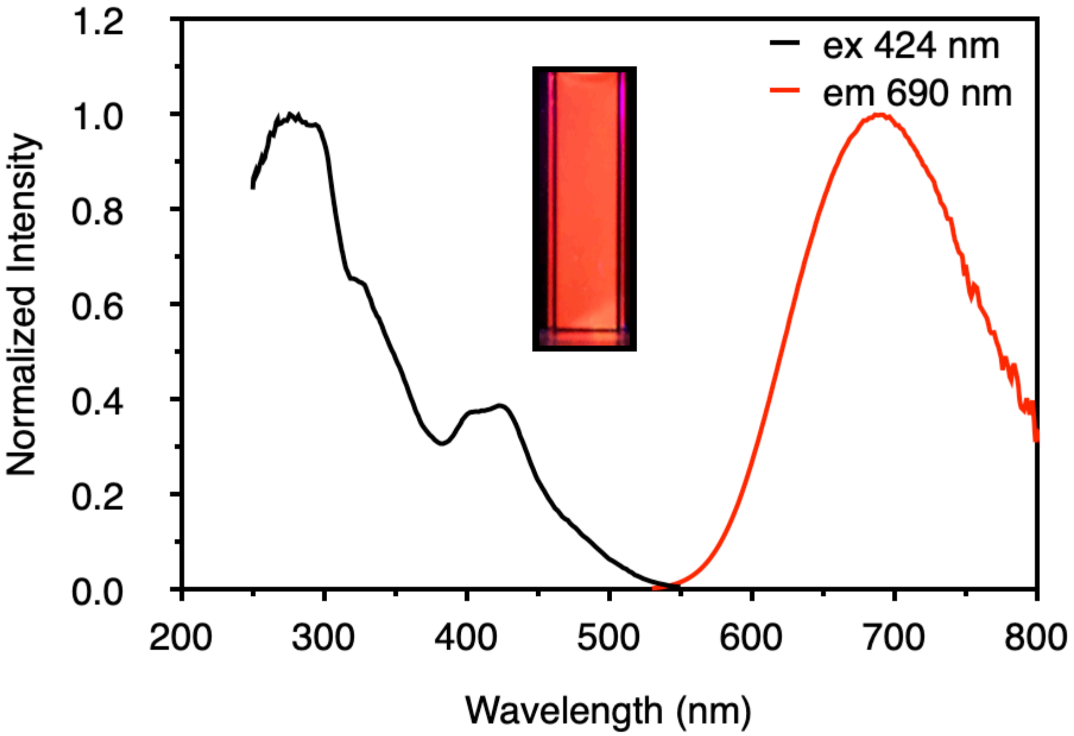

Figure S25. Excitation and photoluminescence spectra of **2*** in CH_2Cl_2. ($c = 1.0 \times 10^{-6}$ mol/L, 300 K)

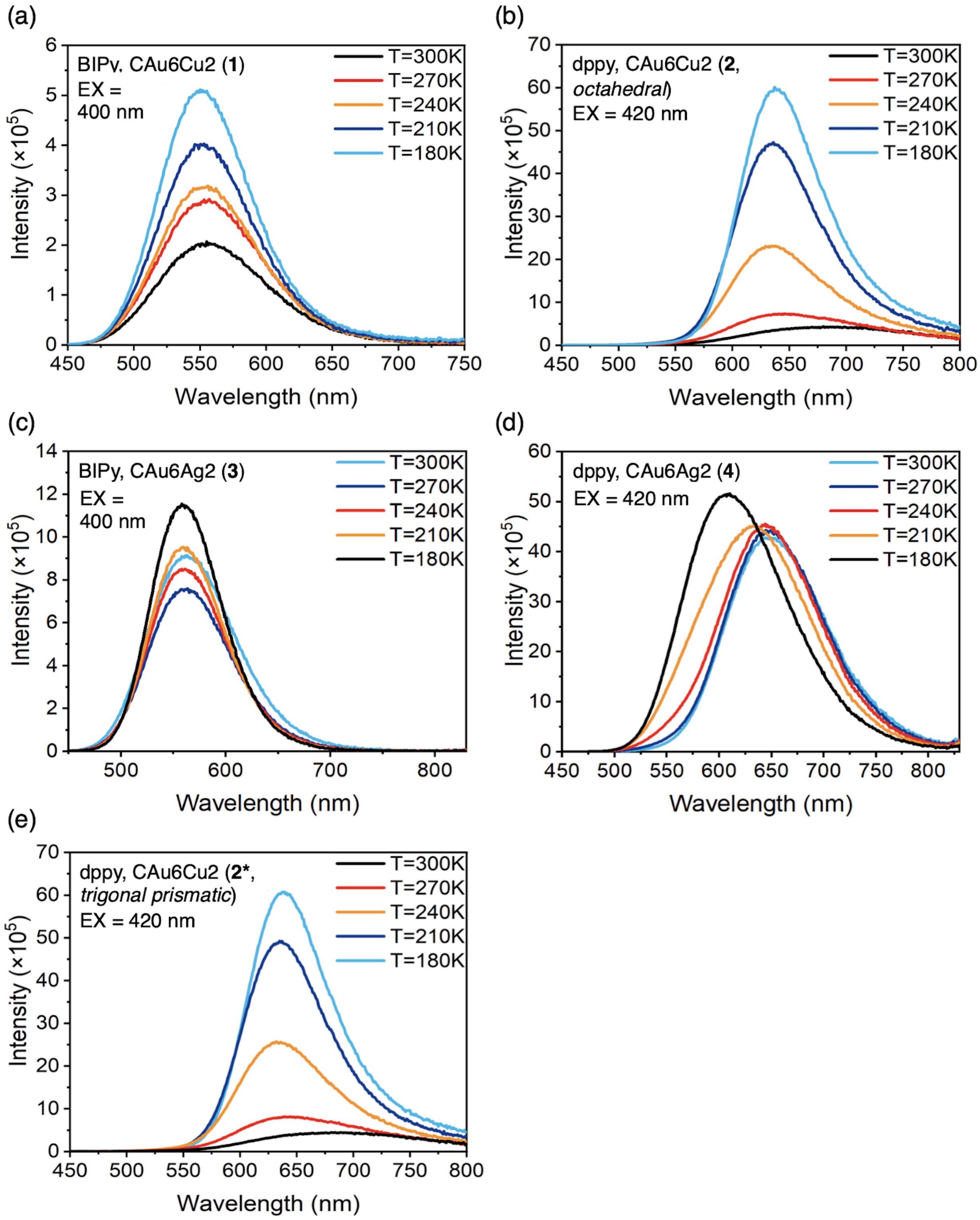

Figure S26. Photoluminescence spectra of (a) **1**, (b) **2**, (c) **3**, (d) **4**, and (e) **2*** in CH_2Cl_2 at 300, 270, 240, 210, and 180 K, respectively. (c = 1.0 × 10^{-6} mol/L)

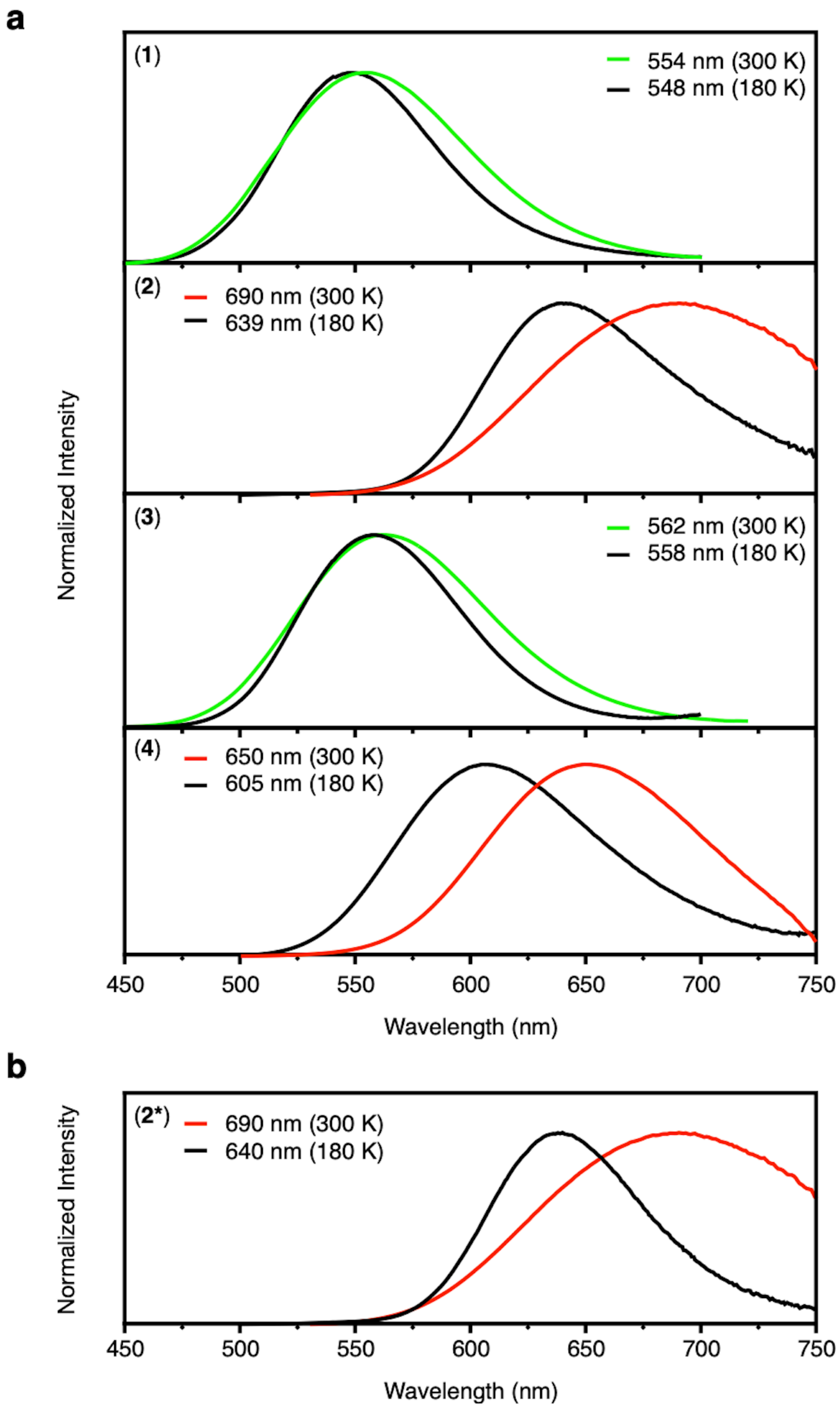

Figure S27. Comparison of the photoluminescence spectra of (a) **1-4** and (b) **2*** in CH_2Cl_2 at 180 and 300 K, respectively. ($c = 1.0 \times 10^{-6}$ mol/L)

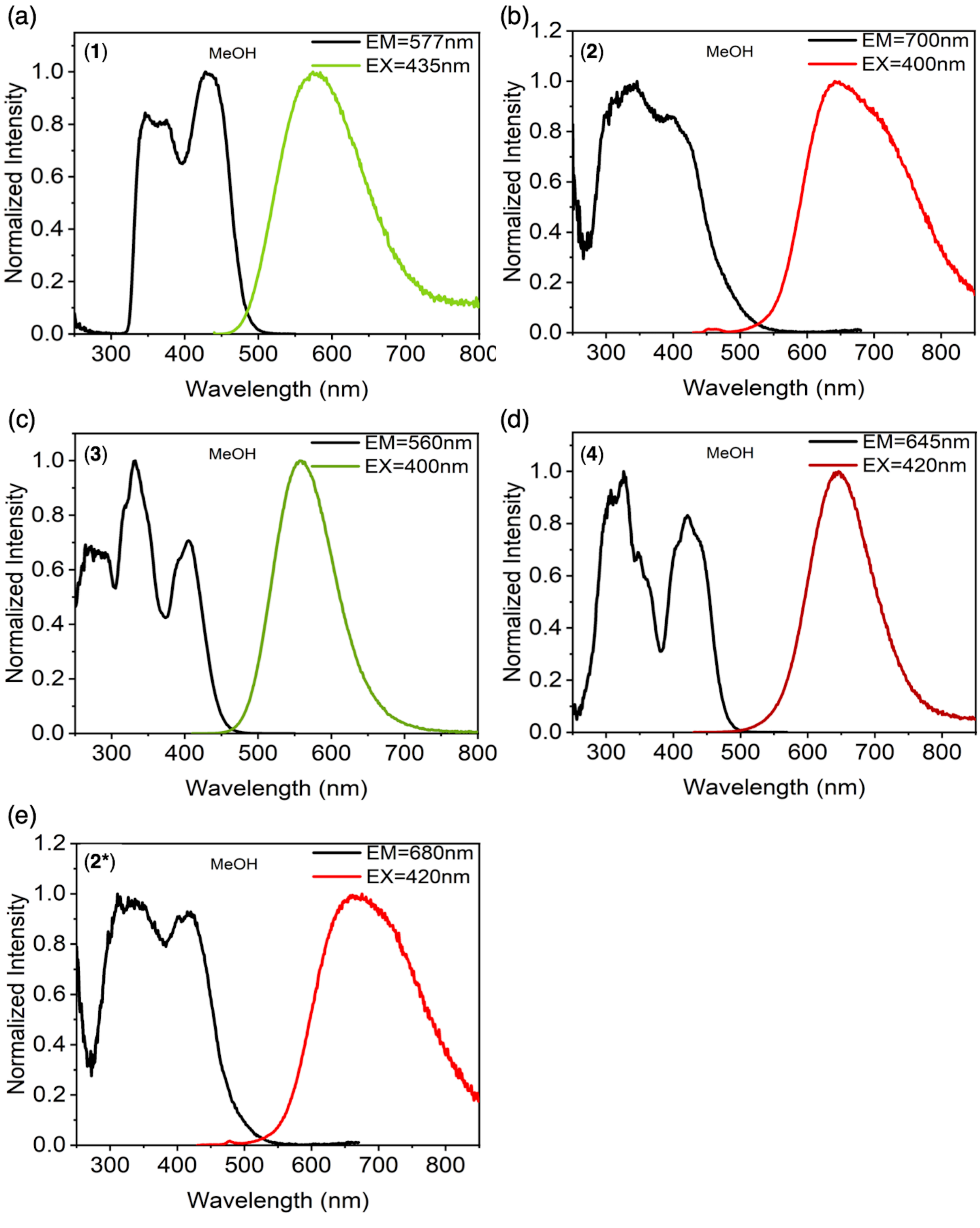

Figure S28. Excitation and photoluminescence spectra of (a) **1**, (b) **2**, (c) **3**, (d) **4**, and (e) **2*** in CH_3OH at 300 K. ($c = 1.0 \times 10^{-6}$ mol/L)

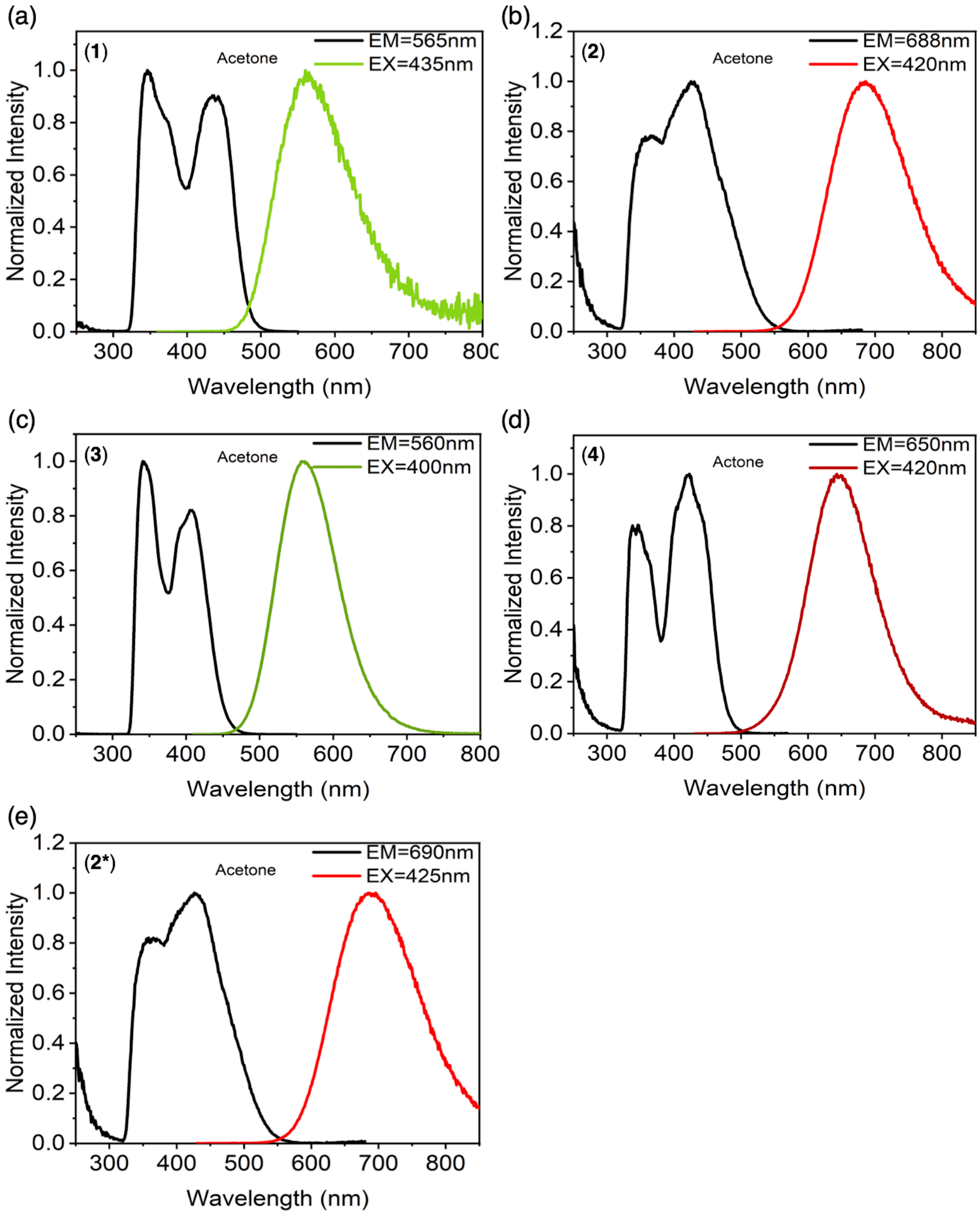

Figure S29. Excitation and photoluminescence spectra of (a) **1**, (b) **2**, (c) **3**, (d) **4**, and (e) **2*** in acetone at 300 K. ($c = 1.0 \times 10^{-6}$ mol/L)

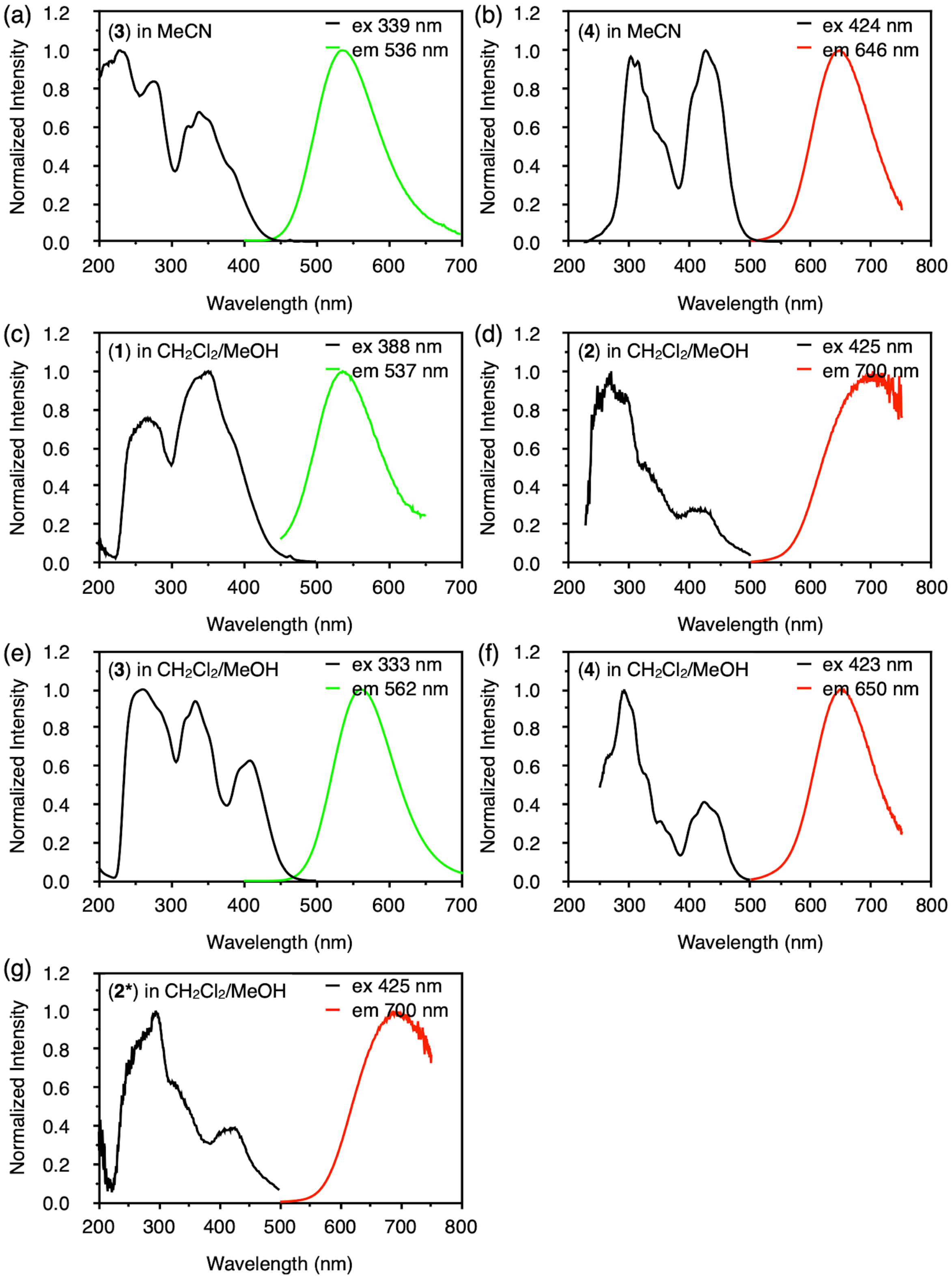

Figure S30. Excitation and photoluminescence spectra of (a) **3** and (b) **4** in CH_3CN, and (c) **1**, (d) **2**, (e) **3**, (f) **4**, and (g) **2*** in CH_2Cl_2/CH_3OH (9:1, v:v) at 300 K. ($c = 1.0 \times 10^{-6}$ mol/L)

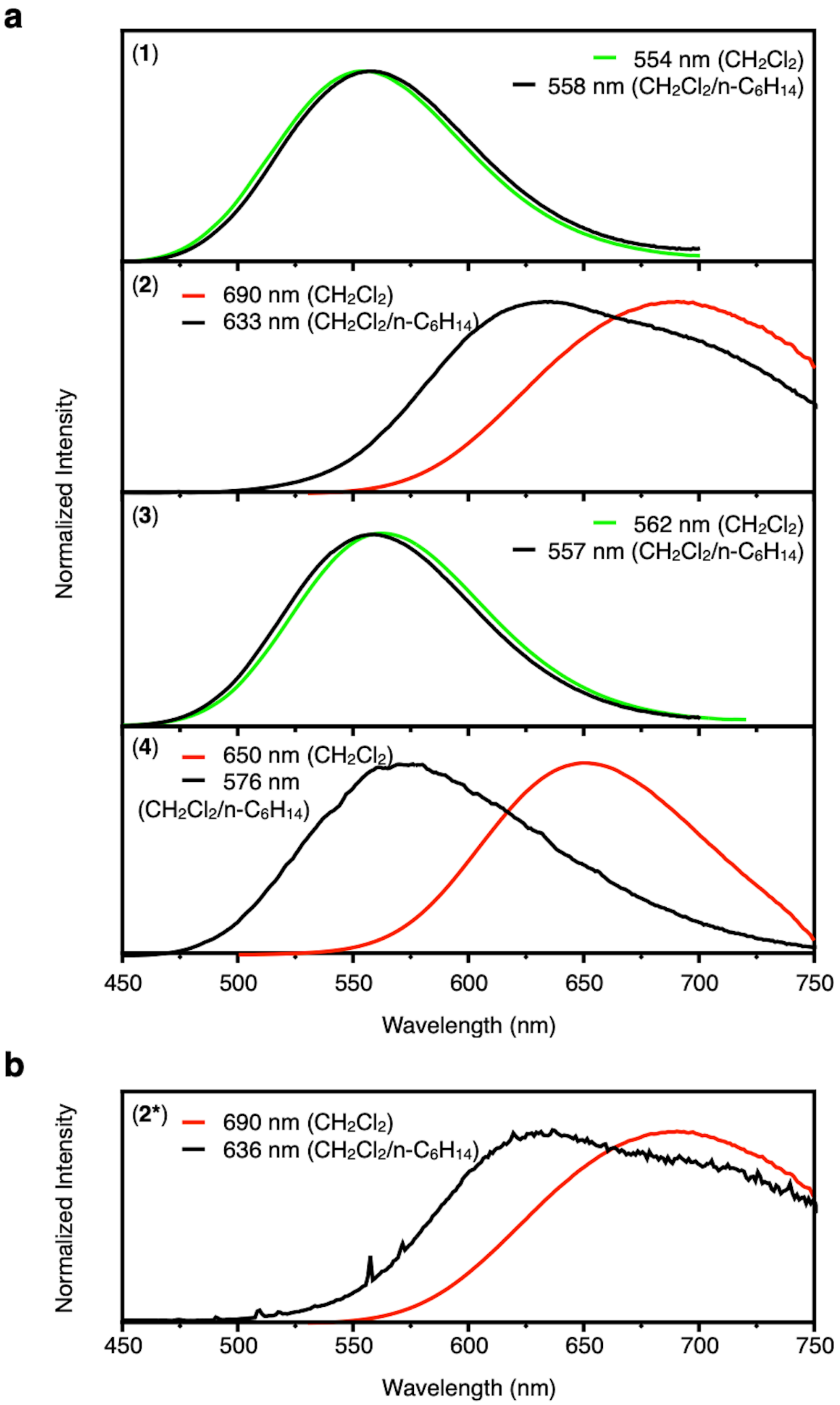

Figure S31. Comparison of the photoluminescence spectra of (a) **1-4** and (b) **2*** in CH_2Cl_2 and in CH_2Cl_2/*n*-hexane (1:9, v:v) at 300 K. ($c = 1.0 \times 10^{-6}$ mol/L)

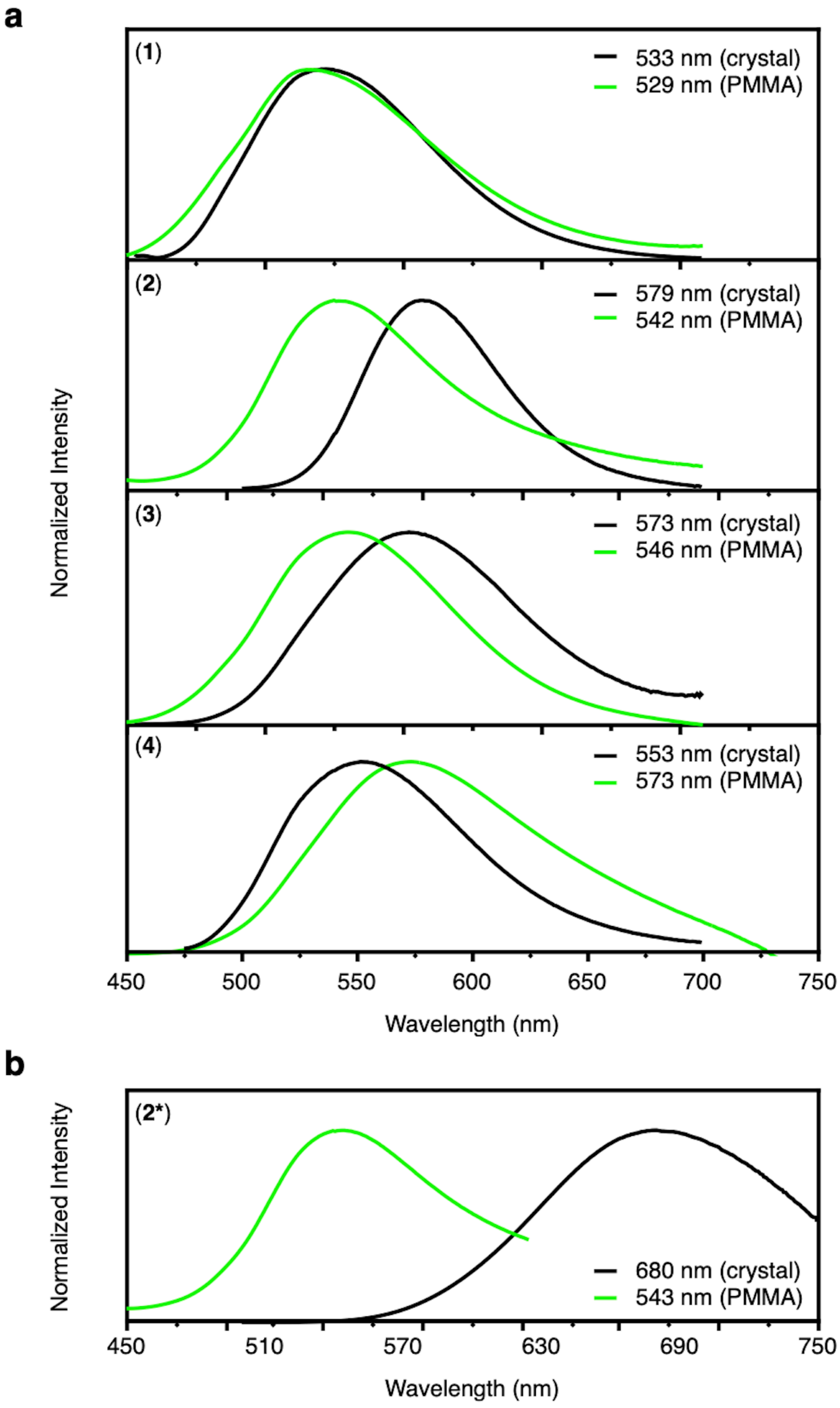

Figure S32. Comparison of the photoluminescence spectra of (a) **1**-**4** and (b) **2*** in the crystalline solid and cluster-containing PMMA films at 300 K.

solid@300K

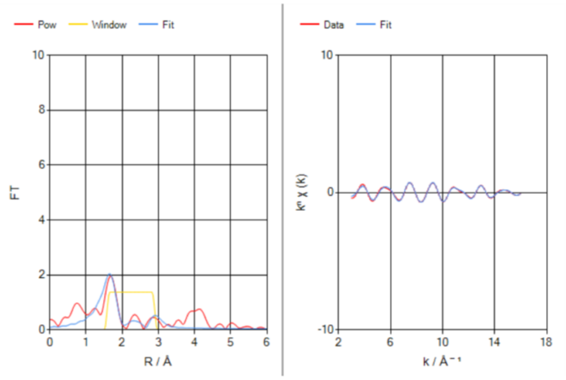

Neighbor atom	C.N.	r (Å)	D.W.	R factor (%)
C	2.0 ± 0.4	2.03 ± 0.08	0.006 ± 0.005	14.7
Cu	1.0 ± 0.4	2.73 ± 0.10	0.013 ± 0.010	
Au	3.8 ± 0.7	2.93 ± 0.10	0.017 ± 0.013	

solution@300K

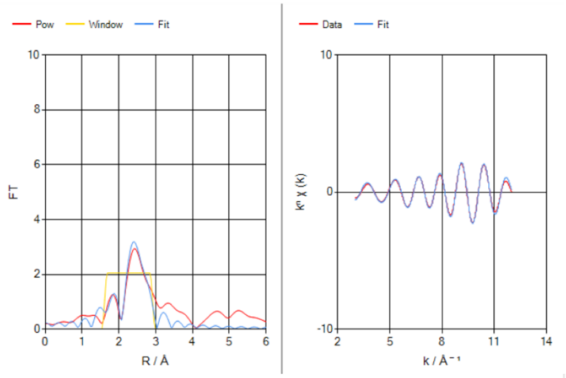

Neighbor atom	C.N.	r (Å)	D.W.	R factor (%)
C	1.7 ± 0.4	2.17 ± 0.08	0.010 ± 0.009	9.3
Cu	1.0 ± 0.2	2.77 ± 0.04	0.004 ± 0.003	
Au	4.3 ± 0.9	2.94 ± 0.13	0.026 ± 0.019	

solid@173K

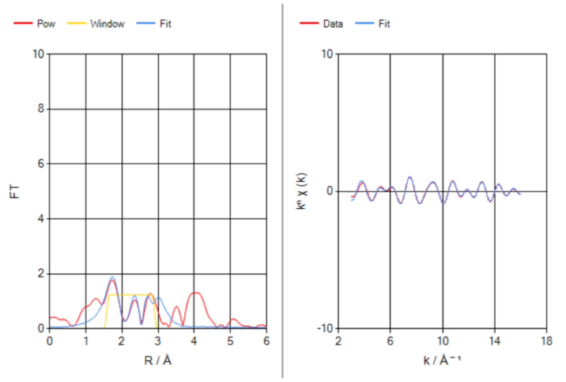

Neighbor atom	C.N.	r (Å)	D.W.	R factor (%)
C	1.9 ± 0.4	2.09 ± 0.07	0.006 ± 0.005	14.8
Cu	0.5 ± 0.2	2.76 ± 0.08	0.006 ± 0.005	
Au	3.9 ± 0.6	2.95 ± 0.07	0.012 ± 0.007	

solution@173K

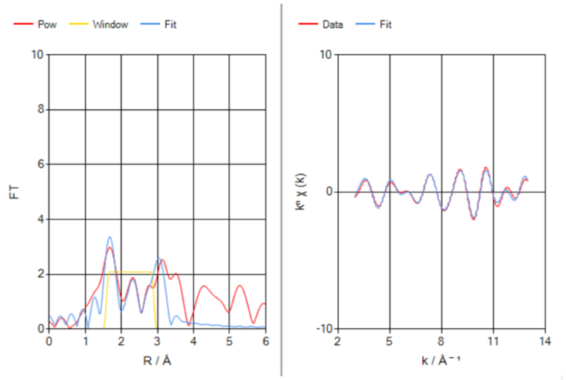

Neighbor atom	C.N.	r (Å)	D.W.	R factor (%)
C	1.8 ± 0.3	2.11 ± 0.05	0.003 ± 0.003	17.1
Cu	1.3 ± 0.3	2.78 ± 0.06	0.008 ± 0.006	
Au	3.6 ± 0.6	2.99 ± 0.06	0.008 ± 0.006	

solid@10K

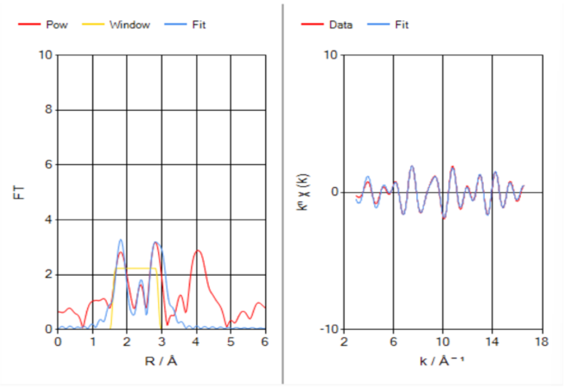

Neighbor atom	C.N.	r (Å)	D.W.	R factor (%)
C	2.1 ± 0.3	2.12 ± 0.05	0.004 ± 0.003	14.8
Cu	1.0 ± 0.3	2.75 ± 0.06	0.009 ± 0.006	
Au	4.4 ± 0.4	2.96 ± 0.05	0.09 ± 0.005	

Figure S33. Au L_3-edge FT-EXAFS spectra and curve-fitting analysis of Au L_3-edge EXAFS spectra of **1** in the solid state and in solution (CH_2Cl_2/CH_3OH, (9:1, v:v)) at different temperatures.

solid@300K

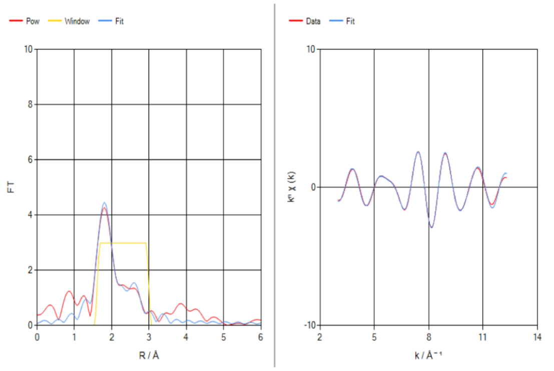

Neighbor atom	C.N.	*r* (Å)	D.W.	R factor (%)
P	1.0 ± 0.2	2.24 ± 0.04	0.003 ± 0.002	6.9
C	1.0 ± 0.3	2.14 ± 0.08	0.005 ± 0.004	
Cu	0.9 ± 0.3	2.82 ± 0.07	0.010 ± 0.007	
Au	4.4 ± 0.3	2.94 ± 0.05	0.023 ± 0.016	

solution@300K

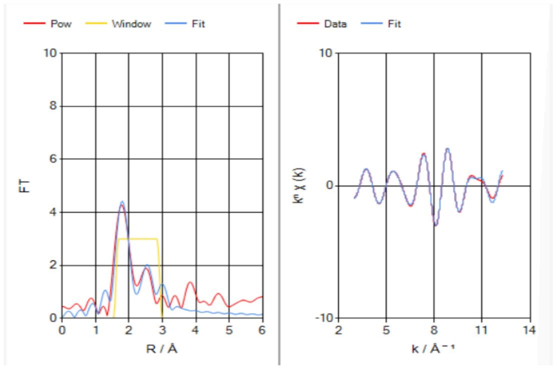

Neighbor atom	C.N.	*r* (Å)	D.W.	R factor (%)
P	1.1 ± 0.1	2.23 ± 0.04	0.002 ± 0.001	8.4
C	1.4 ± 0.8	2.05 ± 0.10	0.023 ± 0.020	
Cu	0.9 ± 0.2	2.82 ± 0.04	0.005 ± 0.004	
Au	2.9 ± 0.4	2.82 ± 0.06	0.012 ± 0.008	

solid@173K

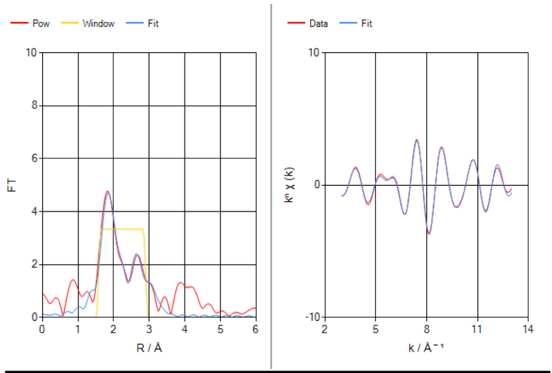

Neighbor atom	C.N.	r (Å)	D.W.	R factor (%)
P	1.2 ± 0.2	2.27 ± 0.05	0.004 ± 0.003	7.3
C	1.3 ± 0.4	2.14 ± 0.08	0.004 ± 0.004	
Cu	1.3 ± 0.5	2.80 ± 0.11	0.017 ± 0.013	
Au	4.2 ± 0.5	2.94 ± 0.07	0.012 ± 0.008	

solution@173K

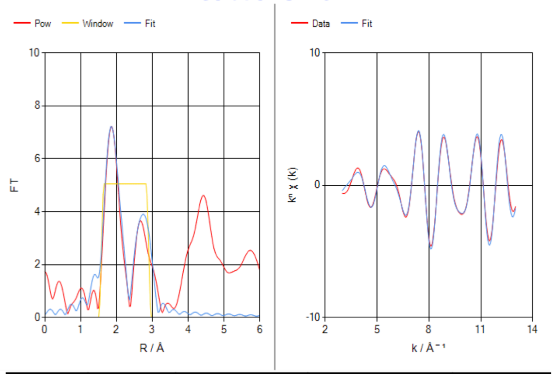

Neighbor atom	C.N.	r (Å)	D.W.	R factor (%)
P	1.3 ± 0.2	2.24 ± 0.03	0.003 ± 0.003	7.9
C	1.4 ± 0.3	2.10 ± 0.08	0.005 ± 0.005	
Cu	1.0 ± 0.4	2.80 ± 0.10	0.014 ± 0.012	
Au	3.4 ± 0.3	2.89 ± 0.04	0.008 ± 0.005	

solid@10K

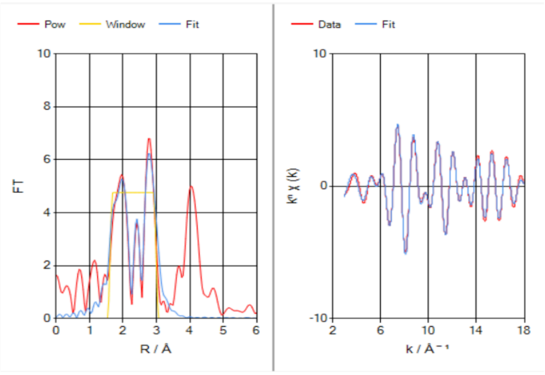

Neighbor atom	C.N.	*r* (Å)	D.W.	R factor (%)
P	1.1 ± 0.1	2.27 ± 0.03	0.002 ± 0.001	12.0
C	1.4 ± 0.2	2.06 ± 0.04	0.003 ± 0.002	
Cu	1.4 ± 0.3	2.83 ± 0.06	0.011 ± 0.007	
Au	4.0 ± 0.2	2.95 ± 0.03	0.007 ± 0.003	

Figure S34. Au L_3-edge FT-EXAFS spectra and curve-fitting analysis of Au L_3-edge EXAFS spectra of **2** in the solid state and in solution (CH_2Cl_2/CH_3OH, (9:1, v:v)) at different temperatures.

solid@300K

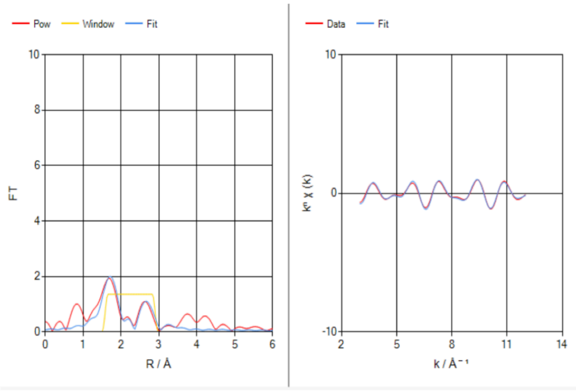

Neighbor atom	C.N.	r (Å)	D.W.	R factor (%)
C	2.0±0.4	2.09±0.07	0.006±0.005	15.0
Ag	1.3±0.4	2.83±0.08	0.013±0.009	
Au	3.9±0.8	2.96±0.13	0.023±0.019	

solution@300K

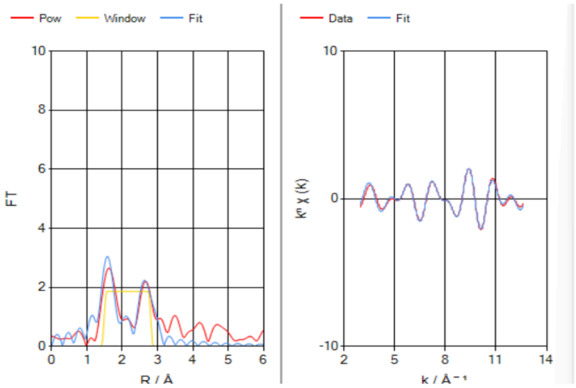

Neighbor atom	C.N.	r (Å)	D.W.	R factor (%)
C	2	2.04±0.06	0.003±0.002	14.1
Ag	1	2.83±0.06	0.006±0.005	
Au	4.3±0.9	2.93±0.06	0.017±0.013	

solid@173K

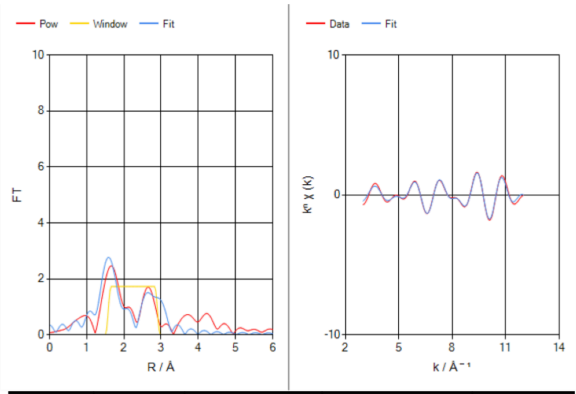

Neighbor atom	C.N.	r (Å)	D.W.	R factor (%)
C	2.1±0.4	2.04±0.06	0.004±0.004	13.2
Ag	0.8±0.3	2.87±0.07	0.006±0.005	
Au	4.0±0.9	2.93±0.10	0.018±0.014	

solution@173K

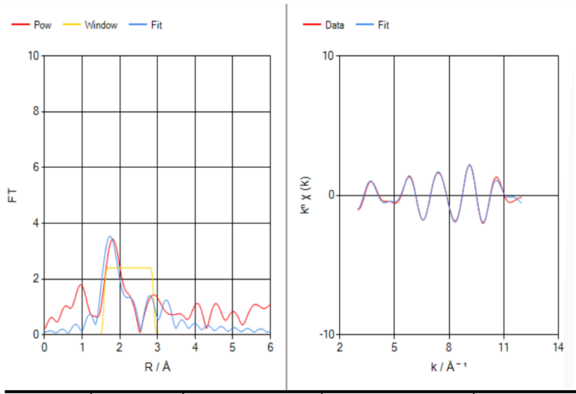

Neighbor atom	C.N.	r (Å)	D.W.	R factor (%)
C	2.3±0.3	2.11±0.05	0.003±0.003	14.3
Ag	0.6±0.2	2.92±0.06	0.003±0.002	
Au	3.5±0.6	2.99±0.08	0.011±0.007	

solid@10K

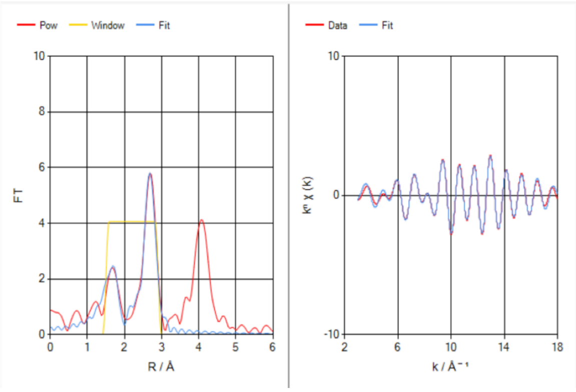

Neighbor atom	C.N.	r (Å)	D.W.	R factor (%)
C	2.0±0.3	2.05±0.05	0.005±0.004	10.8
Ag	1.1±0.1	2.87±0.03	0.004±0.002	
Au	3.9±0.4	2.93±0.05	0.012±0.006	

Figure S35. Au L_3-edge FT-EXAFS spectra and curve-fitting analysis of Au L_3-edge EXAFS spectra of **3** in the solid state and in solution (CH_2Cl_2/CH_3OH, (9:1, v:v)) at different temperatures.

solid@300K

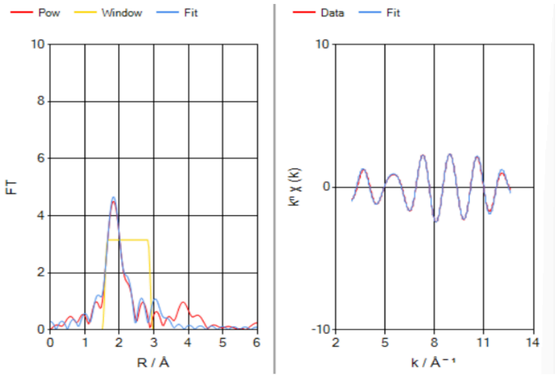

Neighbor atom	C.N.	r (Å)	D.W.	R factor (%)
P	1	2.26 ± 0.04	0.002 ± 0.002	13.3
C	1	2.06 ± 0.08	0.003 ± 0.003	
Ag	1	2.84 ± 0.11	0.014 ± 0.011	
Au	4.2 ± 0.8	2.93 ± 0.06	0.015 ± 0.011	

solution@300K

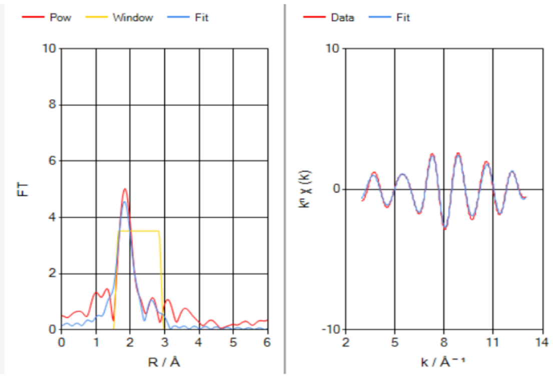

Neighbor atom	C.N.	r (Å)	D.W.	R factor (%)
P	1	2.28 ± 0.04	0.002 ± 0.002	11.8
C	1	2.09 ± 0.06	0.001 ± 0.001	
Ag	1	2.87 ± 0.10	0.012 ± 0.010	
Au	3.1 ± 0.5	2.86 ± 0.07	0.012 ± 0.009	

solid@173K

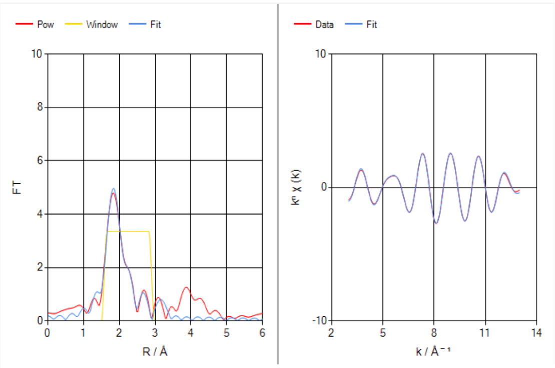

Neighbor atom	C.N.	r (Å)	D.W.	R factor (%)
P	1.0 ± 0.2	2.26 ± 0.04	0.002 ± 0.002	3.8
C	1.0 ± 0.3	2.14 ± 0.09	0.004 ± 0.004	
Ag	1.0 ± 0.3	2.91 ± 0.07	0.008 ± 0.006	
Au	4.0 ± 0.5	2.94 ± 0.07	0.011 ± 0.008	

solution@173K

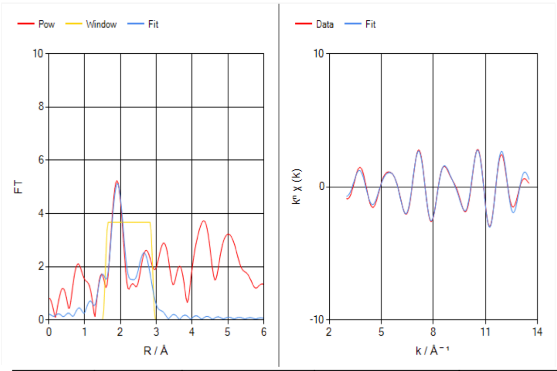

Neighbor atom	C.N.	r (Å)	D.W.	R factor (%)
P	1.4 ± 0.2	2.29 ± 0.04	0.002 ± 0.001	11.5
C	0.9 ± 0.3	2.04 ± 0.09	0.003 ± 0.003	
Ag	1.4 ± 0.3	2.85 ± 0.06	0.008 ± 0.006	
Au	3.4 ± 0.7	2.91 ± 0.10	0.017 ± 0.009	

solid@10K

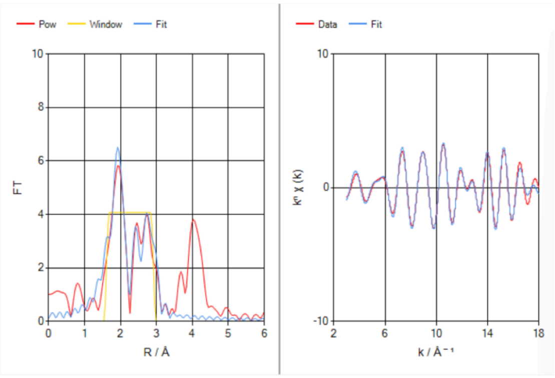

Neighbor atom	C.N.	r (Å)	D.W.	R factor (%)
P	1.2 ± 0.1	2.26 ± 0.03	0.002 ± 0.001	14.5
C	1.2 ± 0.2	2.05 ± 0.06	0.006 ± 0.005	
Ag	1.0 ± 0.1	2.91 ± 0.03	0.004 ± 0.003	
Au	4.1 ± 0.2	2.92 ± 0.03	0.008 ± 0.004	

Figure S36. Au L_3-edge FT-EXAFS spectra and curve-fitting analysis of Au L_3-edge EXAFS spectra of **4** in the solid state and in solution (CH_2Cl_2/CH_3OH, (9:1, v:v)) at different temperatures.

solid@300K

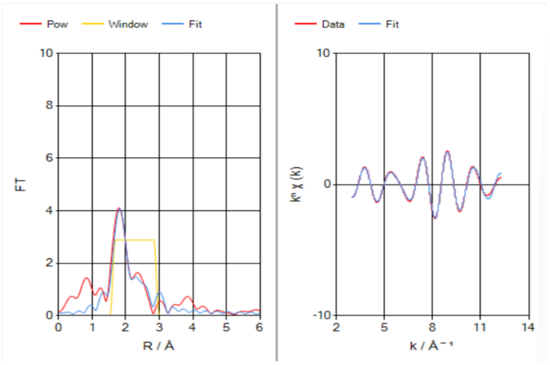

Neighbor atom	C.N.	*r* (Å)	D.W.	R factor (%)
P	1.0 ± 0.2	2.26 ± 0.04	0.002 ± 0.001	9.5
C	1.3 ± 0.4	2.16 ± 0.10	0.012 ± 0.010	
Cu	1.3 ± 0.3	2.84 ± 0.06	0.012 ± 0.008	
Au	3.0 ± 0.4	2.82 ± 0.06	0.013 ± 0.008	

solution@300K

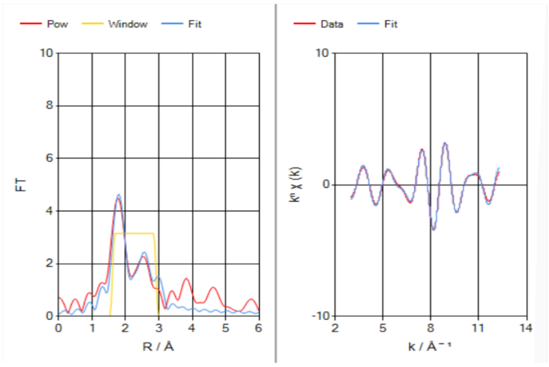

Neighbor atom	C.N.	*r* (Å)	D.W.	R factor (%)
P	1.2 ± 0.1	2.24 ± 0.03	0.003 ± 0.002	8.1
C	1.4 ± 0.7	2.14 ± 0.12	0.023 ± 0.021	
Cu	1.3 ± 0.2	2.84 ± 0.05	0.010 ± 0.007	
Au	2.9 ± 0.3	2.85 ± 0.05	0.010 ± 0.008	

solid@173K

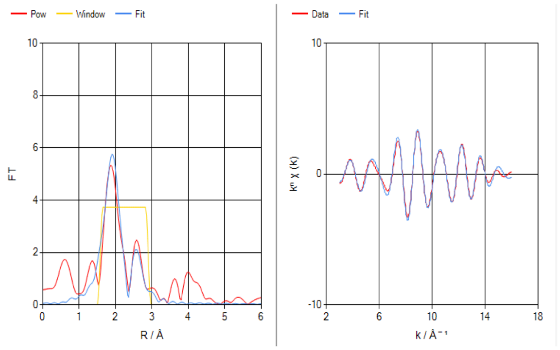

Neighbor atom	C.N.	r (Å)	D.W.	R factor (%)
P	1.3 ± 0.2	2.30 ± 0.05	0.004 ± 0.004	14.5
C	1.4 ± 0.3	2.09 ± 0.07	0.004 ± 0.004	
Cu	1.2 ± 0.4	2.90 ± 0.11	0.016 ± 0.014	
Au	3.1 ± 0.5	2.90 ± 0.07	0.011 ± 0.008	

solution@173K

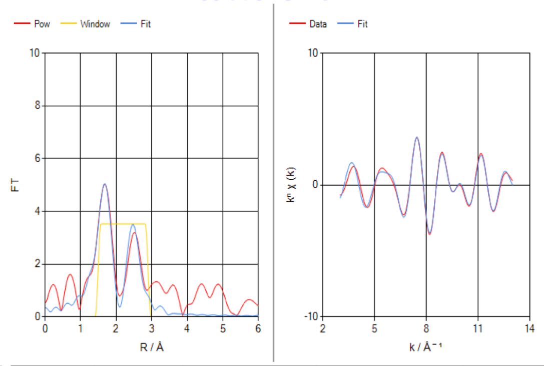

Neighbor atom	C.N.	r (Å)	D.W.	R factor (%)
P	1.2 ± 0.2	2.20 ± 0.04	0.004 ± 0.003	14.0
C	1.4 ± 0.3	2.01 ± 0.06	0.002 ± 0.002	
Cu	0.8 ± 0.2	2.80 ± 0.05	0.004 ± 0.013	
Au	3.5 ± 0.6	2.89 ± 0.05	0.017 ± 0.013	

solid@10K

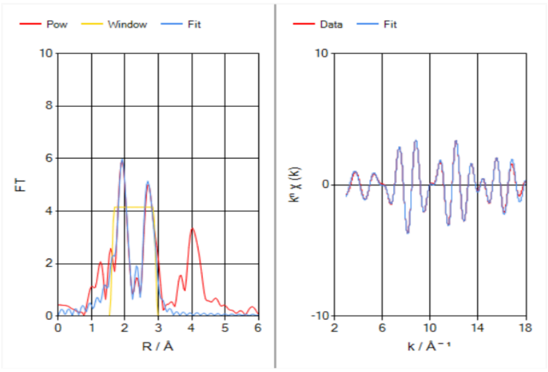

Neighbor atom	C.N.	*r* (Å)	D.W.	R factor (%)
P	1.1 ± 0.1	2.25 ± 0.03	0.002 ± 0.001	8.4
C	1.2 ± 0.8	2.06 ± 0.11	0.012 ± 0.010	
Cu	1.3 ± 0.6	2.89 ± 0.08	0.017 ± 0.012	
Au	2.8 ± 0.2	2.88 ± 0.03	0.006 ± 0.002	

Figure S37. Au L_3-edge FT-EXAFS spectra and curve-fitting analysis of Au L_3-edge EXAFS spectra of **2*** in the solid state and in solution (CH_2Cl_2/CH_3OH, (9:1, v:v)) at different temperatures.

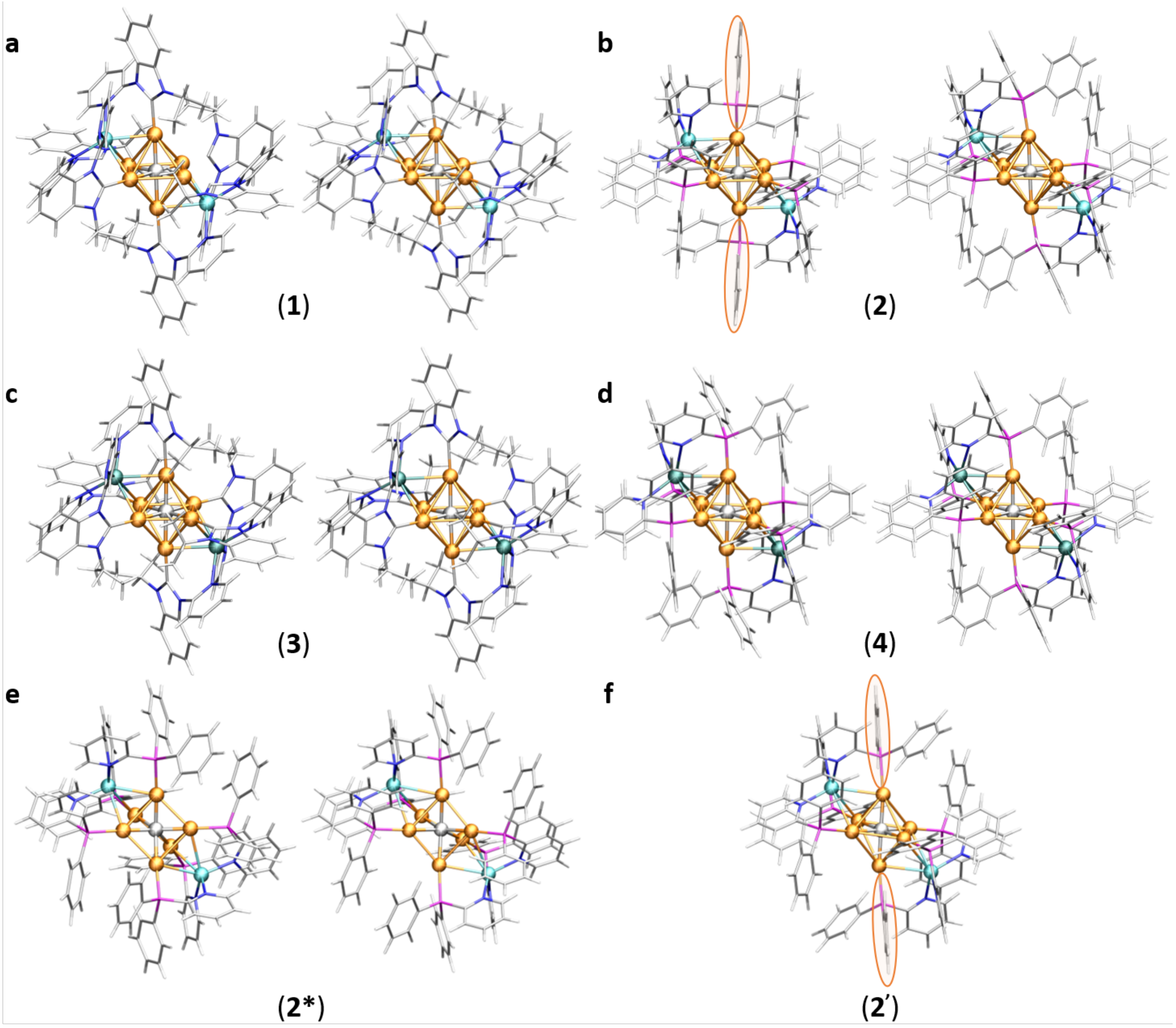

Figure S38. Comparison of the crystal structures (left) of compounds (a) **1**, (b) **2**, (c) **3**, (d) **4**, and (e) **2*** with those optimized at the M06-2X/6-31G*~LANL2DZ level of theory (right). Panel (f) shows the partially optimized structure of **2** (denoted as **2'**), where the dihedral angle between the circled ligand and the Au atom bound to it is constrained to match the value in the crystal structure. Color code: grey C; orange Au; cyan Cu; dark green Ag; magenta P; blue N; white H. The computationally optimized structures were almost identical to the crystal structures, except for **2**. The orientation of the two phenyl groups in **2** showed a large variation after optimization compared with other clusters. To evaluate the effect of ligand orientation on stability, **2'** was obtained with a relative energy of 3.4 kcal mol^{-1}, 1.4 kcal mol^{-1} higher than that of **2*** (Table S4). Therefore, the flexibility of the phosphine ligands may play a role in destabilizing the octahedral structure and eliminate the difference in stability between the octahedral and triangular prism structures.

Table S3. Bond lengths (*d*, in Å) and Wiberg bond orders (WBO) of C–Au, Au···Au, and Au···Cu/Ag in the octahedral and triangular prism structures, calculated at the ωB97XD/6-31G*~LANL2DZ level of theory. Depicted are the octahedral and triangular prism structures of the C-centered metal core. Color code: grey C; orange Au; cyan Cu/Ag.

Bond	**1**		**2**		**3**		**4**	
	d	WBO	*d*	WBO	*d*	WBO	*d*	WBO
C–Au (6[a])	2.16	0.37	2.13 ~2.17	0.38 ~0.36	2.16 ~2.18	0.38 ~0.37	2.14	0.37
Au···Au (12)	3.03 ~3.07	0.16 ~0.14	2.94 ~3.12, 3.32[b]	0.16 ~0.12, 0.10[b]	3.00 ~3.16	0.17 ~0.13	3.01 ~3.03	0.15 ~0.13
Cu/Ag···Au (6)	2.91	0.13	2.90 ~3.10	0.12 ~0.10	2.99	0.11	3.06	0.10
Bond	**1***		**2***		**3***		**4***	
	d	WBO	*d*	WBO	*d*	WBO	*d*	WBO
C–Au (6)	2.20	0.38	2.19	0.38	2.22	0.38	2.20	0.38
Au···Au (9)	2.84 ~2.96	0.16	2.83 ~2.93	0.15	2.82 ~2.99	0.17	2.80 ~2.95	0.16 ~0.15
Cu/Ag···Au (6)	2.96	0.12	3.02	0.11	3.04	0.11	3.10	0.10

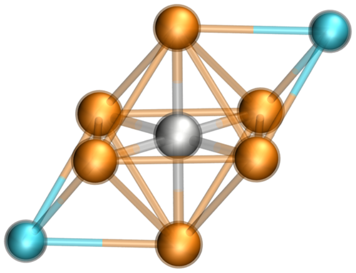

Octahedral

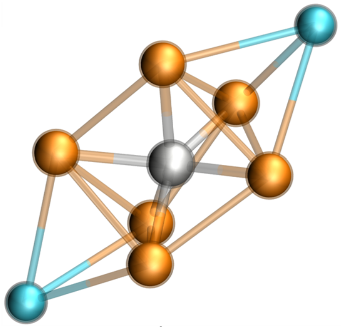

Triangular Prism

[a]The number of bonds or interactions contained in the metal cluster.
[b]The Au···Au distance is longer than other distances due to the distortion of the metal cluster.

Table S4. Relative energies (in kcal mol^{-1}) of the octahedral (**1-4**) and triangular prism (**1*-4***) structures, calculated using the M06-2X functional in the gas phase and various solvent phases (dichloromethane: CH_2Cl_2; *n*-hexane; diethyl ether: Et_2O; CH_2Cl_2/*n*-hexane, v:v = 1:1; CH_2Cl_2/Et_2O, v:v = 1:1), where v:v = 1:1 represents the volume ratio of each component in the mixed solvent. Experimental data showed that NHC-protected clusters (**1** and **3**) retained an octahedral structure in solution, whereas the phosphine-protected clusters (**2** and **4**) underwent a structural transformation to a triangular prism shape (**2*** and **4***). Cluster **2**, crystallized with Et_2O, but transformed to **2*** in CH_2Cl_2. Cluster **2***, crystallized in the presence of *n*-hexane, remained stable in CH_2Cl_2. Meanwhile, the crystal structure of **4** was obtained using either Et_2O or *n*-hexane, but transformed to **4*** in CH_2Cl_2. The dielectric constant (ε) of each solvent is an important parameter in the PCM model and can have a significant effect on the structural transformation of the phosphine-protected clusters. CH_2Cl_2 is a polar solvent with high ε (8.93), and both **2*** and **4*** gain high stability in CH_2Cl_2. In *n*-hexane, a non-polar solvent with a low ε (1.88), **2*** is more stable than **2**, while **4*** has a higher energy than in the gas phase. In Et_2O, a moderately polar solvent with the ε of 4.24, the enhanced stability is observable in **2*** and **4***. In the mixed solvents with the ε values of 5.41 and 6.59, the stability of **2*** and **4*** is further enhanced compared to **2** and **4**, respectively. **2’** exhibits a smaller energy (1.6 kcal mol^{-1}) in *n*-hexane than in other solvents.

	Gas	CH_2Cl_2 ε = 8.93	*n*-hexane ε = 1.88	Et_2O ε = 4.24	CH_2Cl_2/*n*-hexane, v = 1:1 ε = 5.41	CH_2Cl_2/Et_2O, v = 1:1 ε = 6.59
**1**	0.0	0.0	0.0	0.0	0.0	0.0
**1***	6.2	6.9	4.8	7.1	7.1	7.1
**2**	0.0	0.0	2.9	0.0	0.0	0.0
**2’**	3.4	3.3	1.6	3.4	3.3	3.3
**2***	2.0	1.1	0.0	1.5	0.4	0.3
**3**	0.0	0.0	0.0	0.0	0.0	0.0
**3***	8.1	9.0	7.1	9.0	9.1	9.1
**4**	0.0	0.0	0.0	0.0	0.0	0.0
**4***	4.0	3.0	6.3	2.6	2.4	2.3

Table S5. Functional dependence of relative energies of the clusters (**1-4** and **1*-4***) in the gas phase and various solvent phases (dichloromethane: CH_2Cl_2; *n*-hexane; diethyl ether: Et_2O). Cluster $\mathbf{2^{int}}$ is an intermediate structure between **2** and **2***, with the $CuAu_3$ motif rotated by around 30° (Fig. 2f). Triangular prism structures of **1*** and **3*** were obtained using B3LYP, ωB97XD, and M06-2X. Upon B3LYP geometry optimization, **4*** underwent a structural change from a triangular prism to an octahedral configuration (i.e., **4**). The triangular prism structure of **4*** was maintained during optimization with ωB97XD or M06-2X. The ωB97XD and M06-2X data in the gas phase revealed that the phosphine ligands reduce the energy difference between the octahedral and triangular prism structures compared to the NHC ligands in the studied CAu_6Cu_2 and CAu_6Ag_2 clusters.

	Gas			CH_2Cl_2		
	B3LYP	ωB97XD	M06-2X	B3LYP	ωB97XD	M06-2X
**1**	0.0	0.0	0.0	0.0	0.0	0.0
**1***	2.8	5.7	6.2	3.9	6.5	6.9
**2**	0.0	0.0	0.0	0.0	0.0	0.0
$\mathbf{2^{int}}$	9.2	/	/	/	/	/
**2***	7.6	1.7	2.0	7.9	2.0	1.1
**3**	0.0	0.0	0.0	0.0	0.0	0.0
**3***	8.4	8.1	8.1	9.8	9.5	9.0
**4**	0.0	0.0	0.0	/	0.0	0.0
**4***	[a]7.5	3.6	4.0	/	3.4	3.0
	n-hexane			Et_2O		
	B3LYP	ωB97XD	M06-2X	B3LYP	ωB97XD	M06-2X
**1**	0.0	0.0	0.0	0.0	0.0	0.0
**1***	2.8	7.2	4.8	4.1	6.8	7.1
**2**	0.0	0.0	2.9	0.0	0.0	0.0
**2***	10.4	3.7	0.0	7.1	1.6	1.5
**3**	0.0	0.0	0.0	0.0	0.0	0.0
**3***	7.6	11.8	7.1	9.5	9.6	9.0
**4**	/	0.0	0.0	/	0.0	0.0
**4***	/	5.4	6.3	/	2.7	2.6

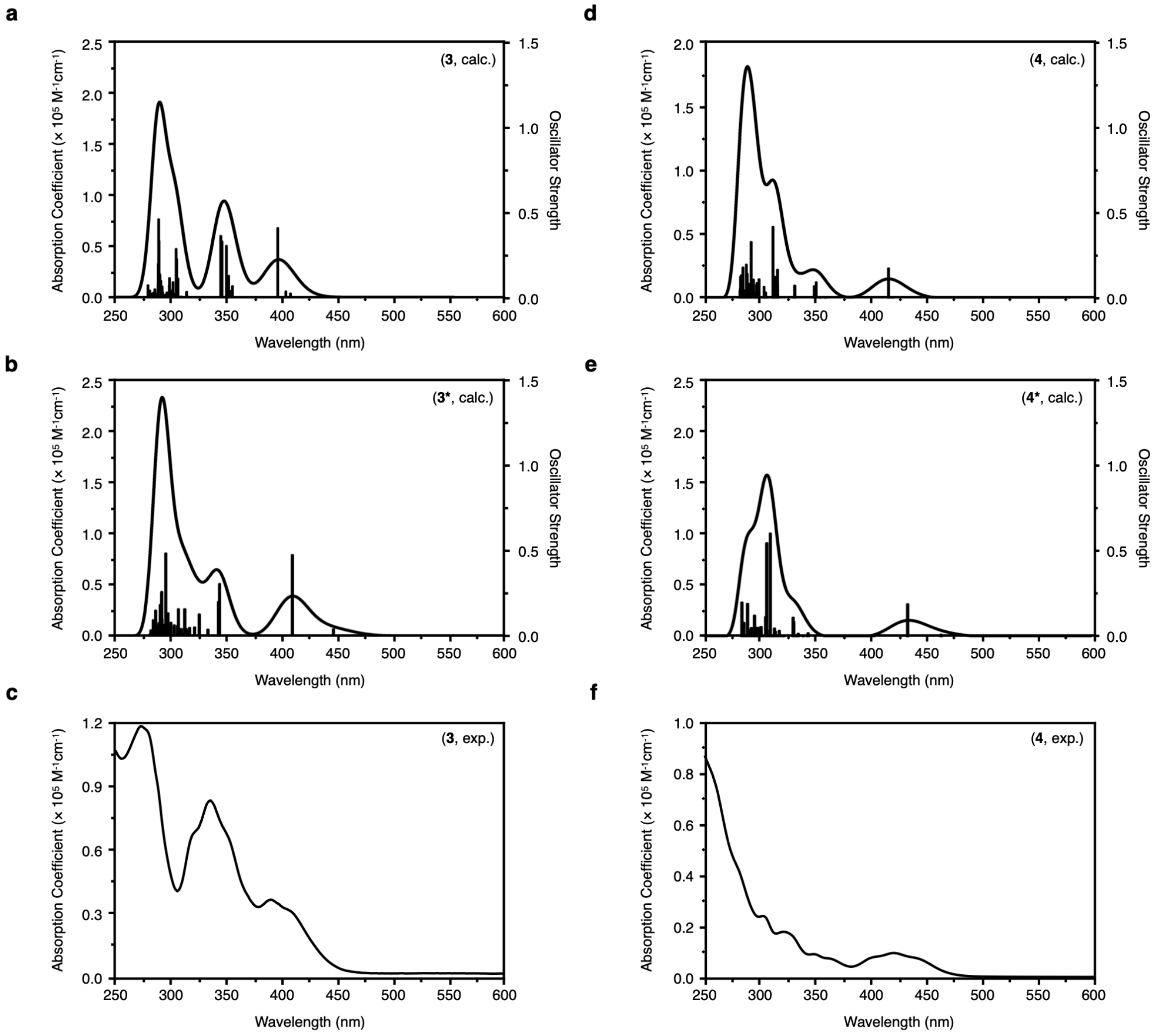

Fig. S39. Comparison of the calculated (by MN15) and experimental UV-vis absorption spectra. Calculated UV-vis absorption spectrum of (a) **3** and (b) **3***; (c) experimental UV-vis absorption spectrum of **3**; calculated UV-vis absorption spectrum of (d) **4** and (e) **4***; (f) experimental UV-vis absorption spectra of **4**. The optimized structures of **3**, **3*** and **4** were obtained by B3LYP, and the optimized structure of **4*** was obtained by ωB97XD.

Table S6. Excited states of **1** with oscillator strength (*f*) greater than 0.02, calculated using MN15/6-31G*(C, N, H) ~ LANL2DZ (Au, Cu). The values in the parenthesis are coefficients for singly excited configurations.

State number	λ (nm)	Δ*E* (eV)	*f*	Transition character
1	361	3.434	0.0330	H→L (0.94)
2	361	3.435	0.0327	H–1→L (0.94)
3	339	3.654	0.3019	H–2→L (0.92)
4	322	3.849	0.1261	H→L+1 (0.69), H–1→L+2 (0.61)
5	319	3.884	0.1583	H–1→L+1 (0.62), H→L+2 (0.58)
6	319	3.885	0.1643	H–1→L+2 (0.65), H→L+1 (–0.56)
7	316	3.923	0.4748	H→L+2 (0.64), H–1→L+1 (–0.60)
8	309	4.011	0.2014	H–2→L+1 (0.82)
9	309	4.013	0.1940	H–2→L+2 (0.82)
12	296	4.190	0.0306	H–17→L (0.68), H–14→L (0.51)
13	296	4.192	0.0300	H–18→L (0.69), H–15→L (0.50)
23	271	4.577	0.0337	H→L+6 (0.67)
24	271	4.578	0.0341	H–1→L+6 (0.68)
31	267	4.646	0.0558	H–2→L+6 (0.56)
35	262	4.727	0.0651	H→L+7 (0.70)
36	262	4.728	0.0756	H→L+8 (0.58), H–1→L+7 (0.50)
37	262	4.728	0.0380	H–1→L+8 (0.68)
42	259	4.789	0.0419	H–2→L+7 (0.58)
43	259	4.790	0.0421	H–2→L+8 (0.58)
46	258	4.815	0.0463	H–21→L (0.57)
48	257	4.825	0.0944	H–23→L (0.43), H–24→L (0.42)
50	257	4.825	0.0984	H–24→L (0.44), H–23→L (–0.43)
51	257	4.826	0.1209	H–2→L+6 (0.48)
54	254	4.875	0.1900	H–2→L+7 (0.34), H–3→L+3 (0.33)
56	254	4.876	0.2101	H–2→L+8 (0.36), H–4→L+3 (0.31)
57	254	4.879	0.0370	H–24→L (0.29), H–2→L+9 (–0.23)
59	254	4.880	0.0296	H–23→L (0.28), H–4→L+3 (–0.26)
62	253	4.896	0.5053	H–21→L (0.33)
66	251	4.941	0.0991	H–16→L (0.37), H–21→L (–0.31)
67	251	4.948	0.6024	H–8→L+1 (0.38)
68	251	4.949	0.6127	H–8→L+2 (0.36)
72	250	4.962	0.0217	H–8→L (0.74)
73	250	4.969	0.0263	H–10→L (0.66)
74	249	4.970	0.0203	H–11→L (0.66)
87	243	5.103	0.1363	H–16→L (0.44), H–1→L+12 (–0.37), H→L+13 (–0.36)
90	242	5.116	0.0512	H–14→L (0.33), H–15→L (0.32)
91	242	5.116	0.0516	H–15→L (0.33), H–14→L (0.31), H→L+12 (–0.31)
92	241	5.136	0.1029	H→L+12 (0.58), H–1→L+13 (–0.48)

94	241	5.140	0.0275	H→L+13 (0.47), H–1→L+12 (–0.47)
95	241	5.141	0.0272	H–1→L+13 (0.51)

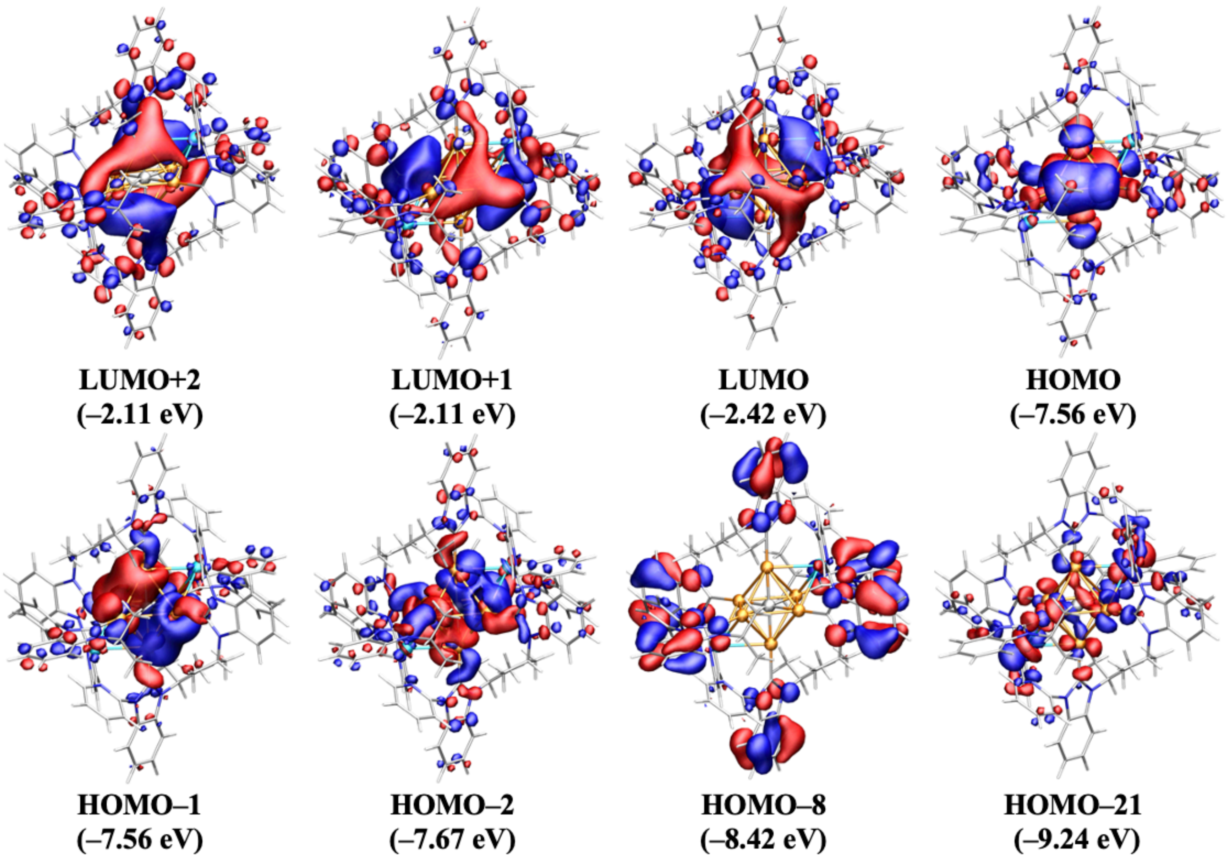

Figure S40. Selected molecular orbitals of **1** (isovalue = 0.02). The HOMO (highest occupied molecular orbital)-LUMO (lowest unoccupied molecular orbital) gap is 5.13 eV. The values in the parenthesis are the energy levels of the molecular orbitals. Color code: Au orange; Cu cyan; C gray; N blue.

Table S7. Orbital composition analysis with Mulliken partition for selected molecular orbitals of **1**.

Orbital	C-centre	Au^{I}_{6}	Cu^{I}_{2}	$(BIPy)_6$
LUMO+2	0.04%	40.60%	6.72%	52.64%
LUMO+1	0.05%	40.81%	6.71%	52.43%
LUMO	0.13%	51.44%	13.94%	34.49%
HOMO	24.65%	53.60%	1.65%	20.11%
HOMO–1	24.64%	53.55%	1.64%	20.16%
HOMO–2	23.67%	44.75%	2.19%	29.29%
HOMO–8	0.00%	0.34%	0.18%	99.47%
HOMO–21	1.51%	17.81%	58.99%	21.69%

Table S8. Excited states of **1*** with oscillator strength (*f*) greater than 0.02, calculated using MN15/6-31G*(C, N, H) ~ LANL2DZ (Au, Cu). The values in the parenthesis are coefficients for singly excited configurations.

State number	λ (nm)	Δ*E* (eV)	*f*	Transition character
1	402	3.081	0.0449	H→L (0.97)
2	402	3.084	0.0451	H–1→L (0.97)
3	354	3.499	0.4709	H–2→L (0.95)
7	314	3.947	0.3959	H–1→L+1 (0.70), H→L+2 (0.58)
8	314	3.951	0.1659	H→L+2 (0.66), H–1→L+1 (–0.54)
9	314	3.951	0.1605	H–1→L+2 (0.67), H→L+1 (0.54)
10	307	4.041	0.0678	H–17→L (0.71), H–15→L (0.41)
11	307	4.041	0.0673	H–18→L (0.71), H–14→L (0.41)
12	293	4.233	0.0272	H–2→L+1 (0.83)
13	293	4.237	0.0268	H–2→L+2 (0.82)
23	274	4.524	0.1494	H–21→L (0.53), H–4→L+1 (–0.36)
24	274	4.526	0.0685	H→L+3 (0.48), H–5→L (0.39)
25	274	4.527	0.0664	H–1→L+3 (0.48), H–6→L (0.36)
28	273	4.537	0.0839	H–4→L+1 (0.41), H–3→L+2 (–0.41)
31	271	4.574	0.1769	H–24→L (0.35), H–4→L+3 (0.28)
32	271	4.575	0.1791	H–23→L (0.35), H–3→L+3 (–0.28)
34	269	4.615	0.0204	H–21→L (0.46), H–16→L (–0.33)
39	267	4.640	0.0408	H–1→L+7 (0.58), H→L+8 (–0.44)
42	265	4.674	0.0720	H–1→L+8 (0.46), H→L+7 (–0.39)
43	265	4.674	0.0686	H–1→L+7 (0.45), H→L+8 (0.39), H–1→L+8 (0.38)
45	264	4.701	0.0443	H–3→L+6 (0.35), H–17→L+3 (–0.25)
46	264	4.703	0.0415	H–4→L+6 (0.35), H–18→L+3 (0.25)
50	261	4.750	0.0380	H–7→L (0.79)
56	260	4.776	0.4187	H–2→L+6 (0.48), H–16→L (0.41), H–7→L (0.36)
60	259	4.792	0.1087	H–9→L (0.38), H–10→L (0.35)
61	259	4.793	0.1007	H–10→L (0.41), H–9→L (–0.34)
69	254	4.875	0.0298	H→L+11 (0.63)
70	254	4.877	0.0343	H–1→L+11 (0.62)
73	251	4.931	0.3044	H–2→L+6 (0.31), H–11→L+4 (0.25)
74	251	4.931	0.0978	H–2→L+3 (0.36), H–11→L+2 (0.25)
75	251	4.932	0.3930	H–9→L+2 (0.27), H–11→L+5 (–0.24)
76	251	4.933	0.2296	H–2→L+6 (0.30), H–10→L+1 (–0.28)
80	249	4.975	0.0541	H–27→L (0.41), H–33→L (–0.31)
81	249	4.976	0.0633	H–28→L (0.41), H–34→L (–0.31)
82	248	4.992	0.0745	H–2→L+4 (0.34), H–2→L+9 (–0.32)
83	248	4.992	0.0626	H–2→L+5 (0.34), H–2→L+10 (–0.32)
84	247	5.015	0.1217	H→L+13 (0.47), H–1→L+12 (–0.44)

90	246	5.050	0.1220	H→L+23 (0.43)
91	245	5.051	0.1206	H–1→L+23 (0.43)
92	245	5.057	0.3680	H–36→L (0.35), H–1→L+12 (–0.28)
93	245	5.065	0.0465	H→L+23 (0.36), H→L+14 (–0.32)
94	245	5.066	0.0488	H–1→L+23 (0.36), H–1→L+14 (–0.32)
95	244	5.091	0.0235	H–27→L (0.23), H–14→L (–0.22), H–33→L (–0.22)
96	244	5.091	0.0252	H–28→L (0.24), H–34→L (–0.22), H–15→L (–0.22)
99	241	5.140	0.0722	H–1→L+16 (0.29), H→L+17 (0.28)
100	241	5.140	0.0698	H→L+16 (0.32), H–1→L+17 (–0.30)

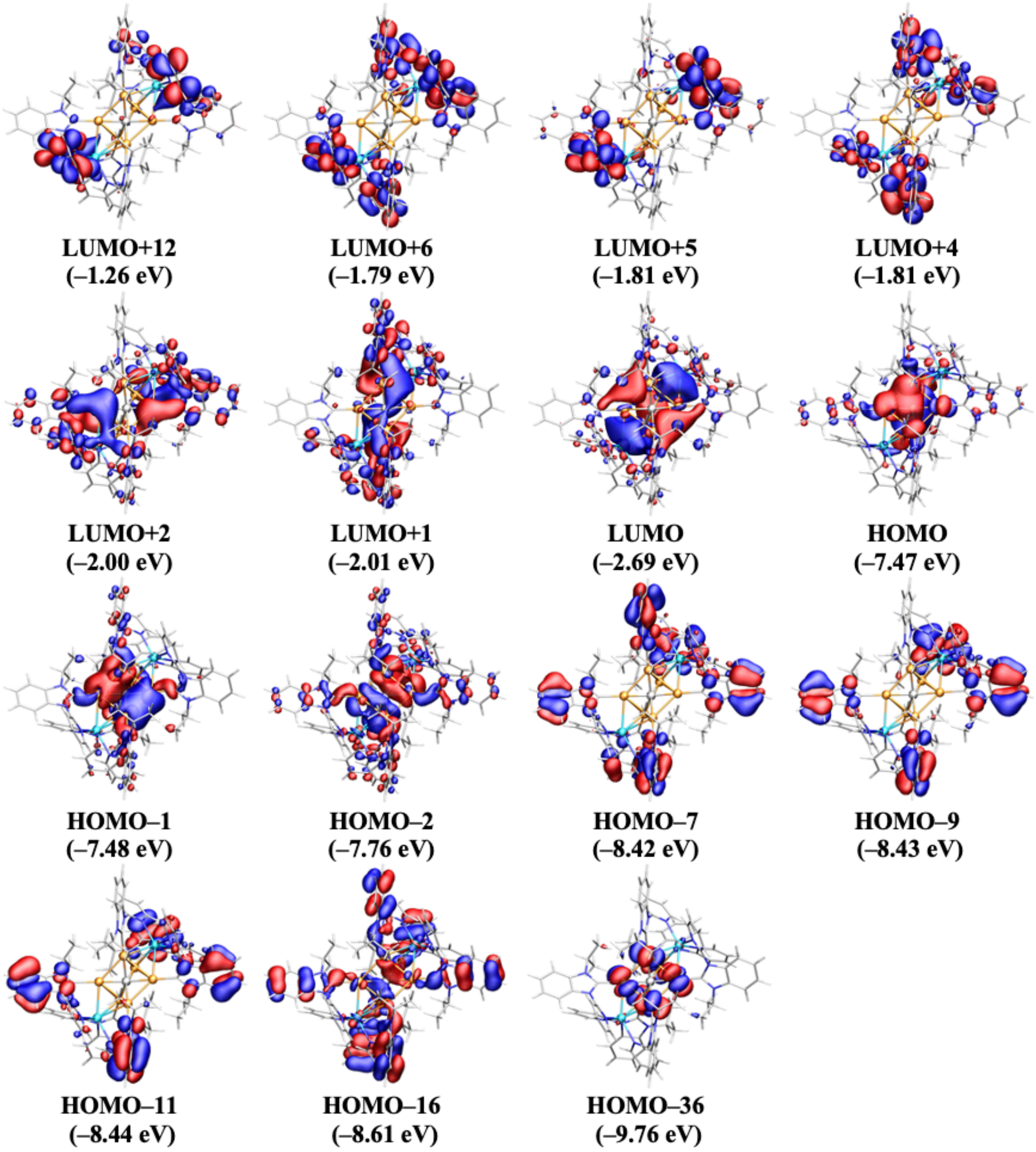

Figure S41. Selected molecular orbitals of **1*** (isovalue = 0.02). The HOMO-LUMO gap is 4.78 eV. The values in the parenthesis are the energy levels of the molecular orbitals. Color code: Au orange; Cu cyan; C gray; N blue.

Table S9. Orbital composition analysis with Mulliken partition for selected molecular orbitals of **1***.

Orbital	C	Au^{I}_{6}	Cu^{I}_{2}	$(BIPy)_6$
LUMO+12	0.24%	9.81%	1.01%	88.94%
LUMO+6	0.02%	5.11%	–0.65%	95.52%
LUMO+5	0.10%	2.68%	3.05%	94.17%
LUMO+4	0.10%	2.72%	3.06%	94.12%
LUMO+2	0.11%	29.97%	6.29%	63.64%
LUMO+1	0.11%	30.30%	6.27%	63.31%
LUMO	0.15%	53.91%	15.39%	30.55%
HOMO	25.84%	53.70%	0.41%	20.04%
HOMO–1	25.82%	53.72%	0.42%	20.04%
HOMO–2	20.62%	42.47%	3.56%	33.35%
HOMO–7	0.01%	0.35%	0.23%	99.41%
HOMO–9	0.08%	0.36%	0.55%	99.01%
HOMO–11	0.00%	0.52%	0.28%	99.20%
HOMO–16	6.54%	12.25%	2.06%	79.15%
HOMO–36	0.43%	87.01%	0.60%	11.96%

Table S10. Excited states of **2** with oscillator strength (*f*) greater than 0.02, calculated using MN15/6-31G*(C, P, N, H) ~ LANL2DZ (Au, Cu). The values in the parenthesis are coefficients for singly excited configurations.

State number	λ (nm)	Δ*E* (eV)	*f*	Transition character
3	366	3.387	0.1728	H→L (0.97)
11	315	3.932	0.1616	H–1→L+1 (0.67), H–2→L+2 (0.64)
12	311	3.983	0.1048	H–1→L+2 (0.55), H–2→L+1 (0.52)
13	311	3.985	0.1055	H–2→L+2 (0.57), H–1→L+1 (–0.50)
17	279	4.450	0.0621	H–35→L (0.51)
18	279	4.451	0.0611	H–34→L (0.56)
19	277	4.478	0.1833	H–36→L (0.55), H–15→L (–0.48)
25	274	4.519	0.0459	H→L+4 (0.42)
31	268	4.619	0.1945	H→L+4 (0.64)
33	267	4.636	0.0692	H–1→L+4 (0.51)
34	267	4.636	0.0724	H–2→L+4 (0.50)
35	266	4.663	0.0718	H–5→L (0.44), H–35→L (0.41)
36	266	4.664	0.0731	H–6→L (0.44), H–34→L (–0.44)
37	264	4.694	0.0324	H–5→L+1 (0.49), H–6→L+2 (–0.46)
41	262	4.739	0.0320	H→L+5 (0.43)
42	262	4.740	0.0330	H→L+6 (0.42)
45	259	4.789	0.0237	H–5→L+2 (0.42), H→L+5 (–0.41), H–6→L+1 (–0.41)
46	259	4.790	0.0256	H–6→L+2 (0.42), H–5→L+1 (0.41), H→L+6 (0.40)
52	256	4.850	0.0794	H–1→L+6 (0.46), H–2→L+5 (0.45)
53	254	4.879	0.0977	H–12→L (0.55)
56	254	4.888	0.1896	H–12→L (0.67)
59	253	4.909	0.0965	H–15→L (0.66)
65	249	4.970	0.2000	H–3→L+3 (0.25)
66	249	4.971	0.1978	H–4→L+3 (0.25)
70	248	4.991	0.0380	H→L+16 (0.38)
74	247	5.027	0.0258	H–21→L (0.72), H–20→L (0.48)
81	244	5.074	0.1003	H–28→L (0.36)
82	244	5.075	0.1026	H–29→L (0.36)
86	243	5.100	0.1647	H–28→L (0.74)
87	243	5.102	0.1624	H–29→L (0.73)
90	242	5.119	0.1171	H–2→L+10 (0.32)
91	242	5.120	0.1185	H–1→L+10 (0.32)
93	242	5.131	0.0215	H→L+16 (0.43)
98	240	5.157	0.1365	H–30→L (0.44), H→L+10 (0.31)
99	240	5.160	0.1000	H–2→L+16 (0.31), H–1→L+10 (0.30)
100	240	5.160	0.1056	H–1→L+16 (0.30), H–2→L+10 (–0.30)

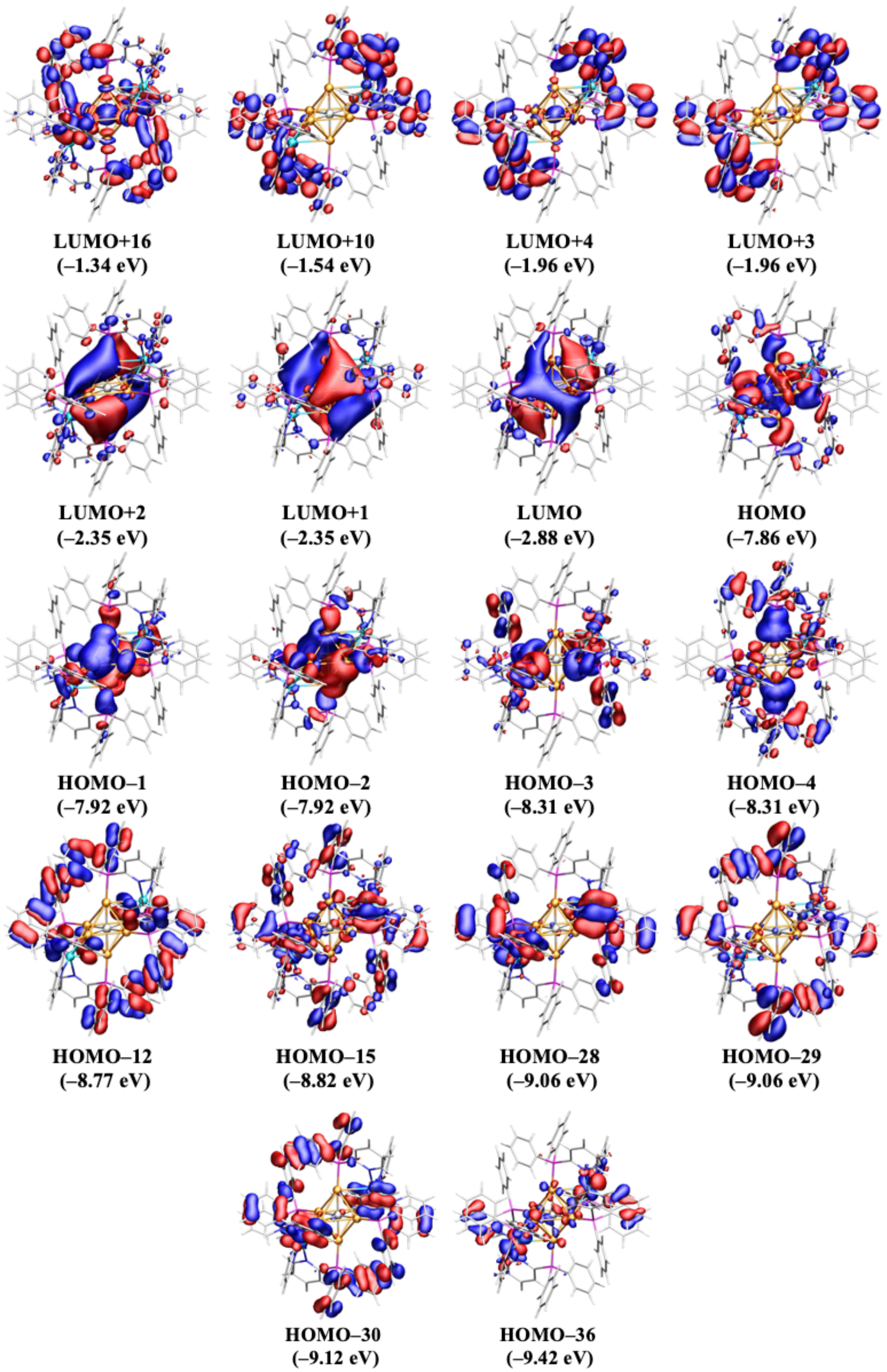

Figure S42. Frontier molecular orbitals of **2** (isovalue = 0.02). The HOMO-LUMO gap is 4.98 eV. The values in the parenthesis are the energy levels of the molecular orbitals. Color code: Au orange; Cu cyan; C gray; N blue.

Table S11. Orbital composition analysis with Mulliken partition for selected molecular orbitals of **2**.

Orbital	C-centre	Au^{I}_{6}	Cu^{I}_{2}	$(dppy)_6$
LUMO+16	4.79%	19.52%	2.36%	73.32%
LUMO+10	0.33%	3.90%	0.09%	95.67%
LUMO+4	1.44%	10.35%	–0.06%	88.27%
LUMO+3	1.01%	0.69%	3.27%	95.02%
LUMO+2	0.06%	67.99%	–1.03%	32.98%
LUMO+1	0.06%	68.19%	–1.04%	32.79%
LUMO	0.08%	60.38%	11.95%	27.59%
HOMO	27.14%	32.28%	2.62%	37.96%
HOMO–1	28.75%	44.25%	2.43%	24.57%
HOMO–2	28.74%	44.36%	2.43%	24.48%
HOMO–3	0.03%	30.73%	15.04%	54.20%
HOMO–4	0.03%	30.73%	15.09%	54.15%
HOMO–12	0.02%	0.73%	0.27%	98.98%
HOMO–15	0.99%	16.58%	5.95%	76.47%
HOMO–28	0.58%	3.34%	5.30%	90.78%
HOMO–29	0.57%	3.33%	5.34%	90.76%
HOMO–30	0.60%	4.54%	1.93%	92.93%
HOMO–36	1.18%	8.58%	62.45%	27.79%

Table S12. Excited states of **2*** with oscillator strength (*f*) greater than 0.02, calculated using MN15/6-31G*(C, P, N, H) ~ LANL2DZ (Au, Cu). The values in the parenthesis are coefficients for singly excited configurations.

State number	λ (nm)	Δ*E* (eV)	*f*	Transition character
3	388	3.198	0.2158	H–2→L (0.97)
14	294	4.213	0.0959	H–33→L (0.67), H–18→L (0.58)
15	293	4.234	0.0885	H–5→L (0.48), H–35→L (–0.44)
16	293	4.235	0.0886	H–6→L (0.48), H–36→L (0.44)
22	286	4.328	0.0604	H–1→L+3 (0.53)
23	286	4.328	0.0620	H→L+3 (0.53)
27	283	4.388	0.1592	H–5→L (0.38)
28	282	4.389	0.1604	H–6→L (0.38)
29	279	4.452	0.3718	H–1→L+6 (0.40), H→L+5 (0.40)
37	273	4.544	0.0784	H–2→L+3 (0.33)
38	272	4.551	0.1705	H–2→L+3 (0.64)
49	266	4.653	0.4442	H–18→L (0.48)
50	266	4.654	0.1148	H–1→L+4 (0.33)
51	266	4.654	0.0806	H→L+4 (0.33)
52	265	4.675	0.0563	H–4→L+2 (0.38), H–3→L+1 (–0.36)
59	262	4.726	0.0879	H→L+4 (0.32)
60	262	4.726	0.0896	H–1→L+4 (0.31)
63	261	4.755	0.3748	H–1→L+8 (0.31), H→L+7 (0.31)
68	257	4.821	0.0672	H–25→L (0.55)
69	257	4.822	0.0669	H–26→L (0.56)
70	256	4.838	0.0487	H–3→L+6 (0.37), H–4→L+5 (–0.36), H–27→L (0.35)
72	255	4.859	0.1284	H→L+8 (0.29), H–1→L+7 (0.28)
73	255	4.859	0.1289	H→L+7 (0.29), H–1→L+8 (–0.29)
78	253	4.896	0.0539	H–30→L (0.88)
82	249	4.972	0.0804	H→L+3 (0.28), H→L+11 (–0.26), H–2→L+7 (–0.25)
83	249	4.972	0.0767	H–1→L+3 (0.27), H–2→L+8 (–0.26), H–1→L+11 (–0.26)
84	248	4.992	0.0663	H→L+3 (0.30), H–4→L+3 (–0.30), H→L+11 (–0.30)
85	248	4.992	0.0755	H–1→L+3 (0.30), H–1→L+11 (–0.30), H–3→L+3 (0.29)
90	246	5.034	0.1998	H–2→L+10 (0.33)
91	246	5.034	0.1997	H–2→L+9 (0.33)
94	245	5.063	0.1636	H–49→L (0.59)
95	245	5.064	0.1660	H–50→L (0.59)
97	244	5.090	0.0344	H–46→L (0.33)
98	244	5.090	0.0321	H–47→L (0.31), H–39→L (0.29)
99	243	5.095	0.1746	H–39→L (0.31), H–2→L+10 (0.30)

100	243	5.096	0.1612	H–40→L (0.33), H–2→L+9 (–0.30)

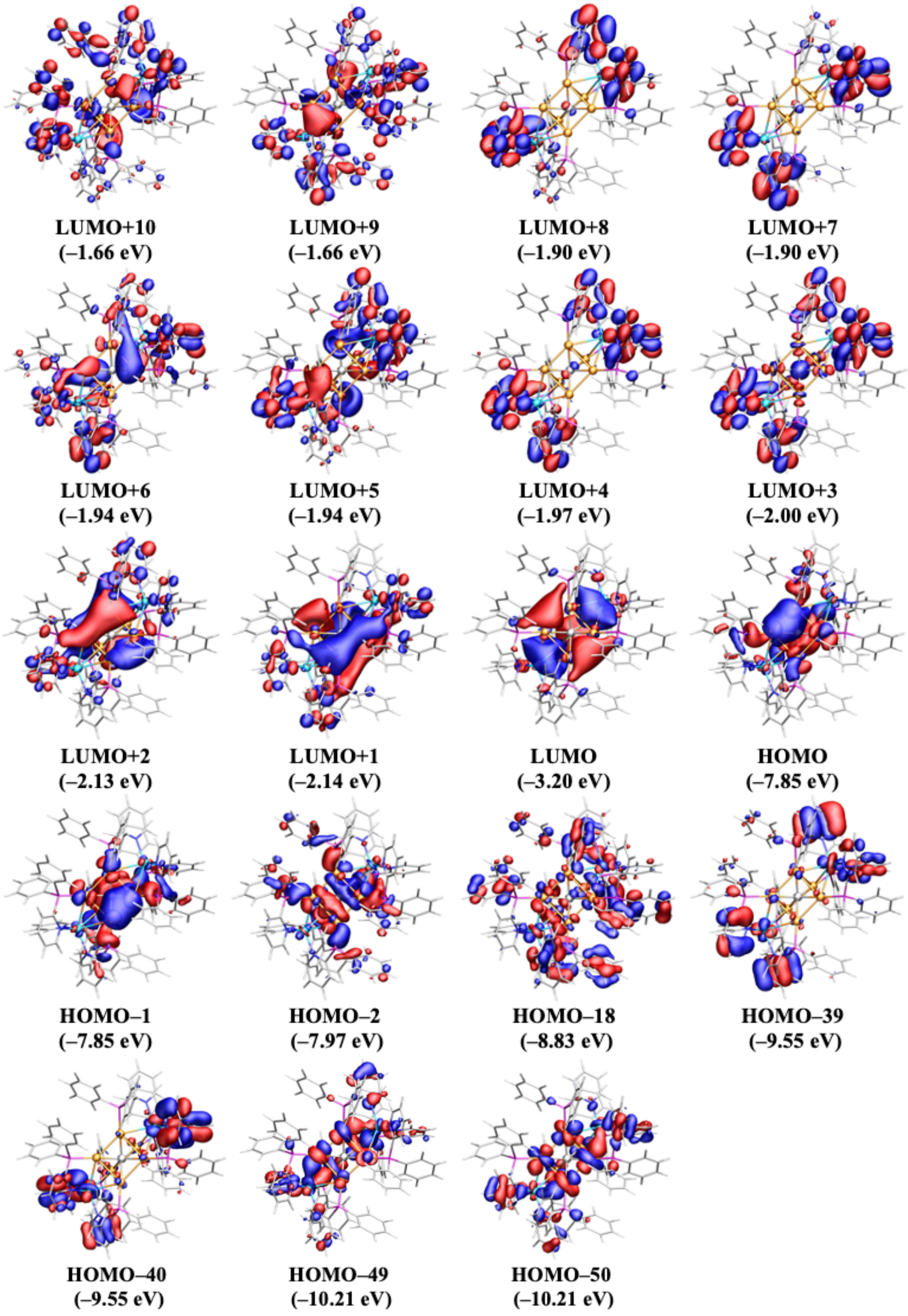

Figure S43. Selected molecular orbitals of **2*** (isovalue = 0.02). The HOMO-LUMO gap is 4.64 eV. The values in the parenthesis are the energy levels of the molecular orbitals. Color code: Au orange; Cu cyan; C gray; N blue.

Table S13. Orbital composition analysis with Mulliken partition for selected molecular orbitals of **2***.

Orbital	C-centre	Au^I_6	Cu^I_2	$(dppy)_6$
LUMO+10	0.08%	27.80%	1.74%	70.38%
LUMO+9	0.08%	27.81%	1.73%	70.38%
LUMO+8	0.74%	1.34%	1.91%	96.02%
LUMO+7	0.73%	1.35%	1.89%	96.03%
LUMO+6	0.11%	29.40%	3.61%	66.88%
LUMO+5	0.11%	29.26%	3.60%	67.02%
LUMO+4	1.03%	0.18%	4.07%	94.72%
LUMO+3	2.29%	13.66%	0.12%	83.93%
LUMO+2	0.12%	52.11%	0.30%	47.48%
LUMO+1	0.11%	52.17%	0.31%	47.41%
LUMO	0.13%	62.24%	12.46%	25.17%
HOMO	28.35%	45.16%	1.74%	24.75%
HOMO–1	28.33%	45.17%	1.76%	24.74%
HOMO–2	25.67%	31.44%	2.72%	40.17%
HOMO–18	1.03%	14.36%	8.78%	75.83%
HOMO–39	0.04%	4.36%	19.39%	76.22%
HOMO–40	0.04%	4.39%	19.16%	76.41%
HOMO–49	0.01%	65.12%	6.54%	28.33%
HOMO–50	0.01%	65.11%	6.54%	28.34%

Table S14. Excited states of **3** with oscillator strength (*f*) greater than 0.02, calculated using MN15/6-31G*(C, N, H) ~ LANL2DZ (Au, Ag). The values in the parenthesis are coefficients for singly excited configurations.

State number	λ (nm)	Δ*E* (eV)	*f*	Transition character
1	380	3.266	0.0239	H–1→L (0.97)
2	375	3.308	0.0355	H–2→L (0.97)
3	367	3.382	0.4107	H→L (0.96)
4	320	3.872	0.0673	H→L+1 (0.83)
5	318	3.903	0.0385	H→L+2 (0.64), H–2→L+1 (0.57)
6	316	3.921	0.1275	H–1→L+2 (0.66), H–2→L+1 (–0.56)
7	314	3.950	0.3056	H–1→L+1 (0.75)
8	309	4.008	0.3313	H–1→L+2 (0.65)
9	308	4.024	0.3635	H–2→L+2 (0.85)
14	273	4.539	0.0331	H–17→L (0.87)
15	273	4.542	0.0313	H–18→L (0.78)
21	264	4.692	0.1113	H→L+4 (0.74)
22	263	4.711	0.0742	H–13→L+1 (0.38)
23	263	4.715	0.2276	H–14→L (0.59)

25	262	4.726	0.2867	H–15→L (0.60)
31	260	4.776	0.0203	H–1→L+6 (0.36), H–2→L+4 (0.35)
33	259	4.784	0.0898	H–16→L (0.55)
34	259	4.789	0.0504	H–16→L (0.48)
35	258	4.797	0.0388	H→L+8 (0.64)
36	258	4.800	0.0421	H→L+7 (0.54)
42	257	4.831	0.0208	H–10→L (0.43), H–9→L (–0.41)
45	256	4.851	0.1146	H–1→L+8 (0.42)
46	255	4.856	0.0750	H–2→L+11 (0.47)
48	254	4.887	0.0218	H–1→L+7 (0.73)
51	253	4.903	0.0295	H–2→L+7 (0.63)
57	249	4.988	0.0318	H→L+4 (0.30), H–11→L+1 (0.29)
58	248	4.998	0.0250	H→L+7 (0.34)
59	248	5.000	0.0641	H–10→L+1 (0.26), H→L+8 (0.25)
63	247	5.028	0.0981	H–2→L+7 (0.30)
64	246	5.035	0.0994	H–17→L+1 (0.20), H–1→L+8 (0.19)
65	246	5.039	0.0479	H–2→L+8 (0.19)
67	246	5.047	0.1353	H–17→L+2 (0.22), H–13→L+6 (0.22), H–1→L+8 (0.22)
68	245	5.064	0.3338	H–17→L+2 (0.34), H–18→L+1 (0.33)
69	245	5.069	0.3140	H–16→L+1 (0.21), H–15→L+2 (–0.20)
70	244	5.073	0.4623	H–14→L (0.26), H–14→L+2 (0.25)
71	244	5.079	0.1998	H–28→L (0.22)
72	244	5.082	0.0858	H–15→L+1 (0.21), H–14→L+2 (–0.21)
74	243	5.110	0.0213	H–5→L+1 (0.36)
79	241	5.155	0.0482	H–29→L (0.34)
80	240	5.161	0.0272	H–30→L (0.31)
81	239	5.183	0.0274	H→L+12 (0.55), H→L+13 (–0.45)
82	239	5.189	0.0269	H→L+15 (0.59)
86	237	5.240	0.0201	H–1→L+15 (0.42)
87	236	5.245	0.0237	H–1→L+12 (0.46)
91	236	5.260	0.0220	H→L+13 (0.25), H–8→L+1 (–0.25)
92	236	5.264	0.0258	H→L+14 (0.32)
94	235	5.269	0.0404	H–29→L (0.28)
100	233	5.312	0.0724	H–25→L (0.45), H–23→L (–0.38)

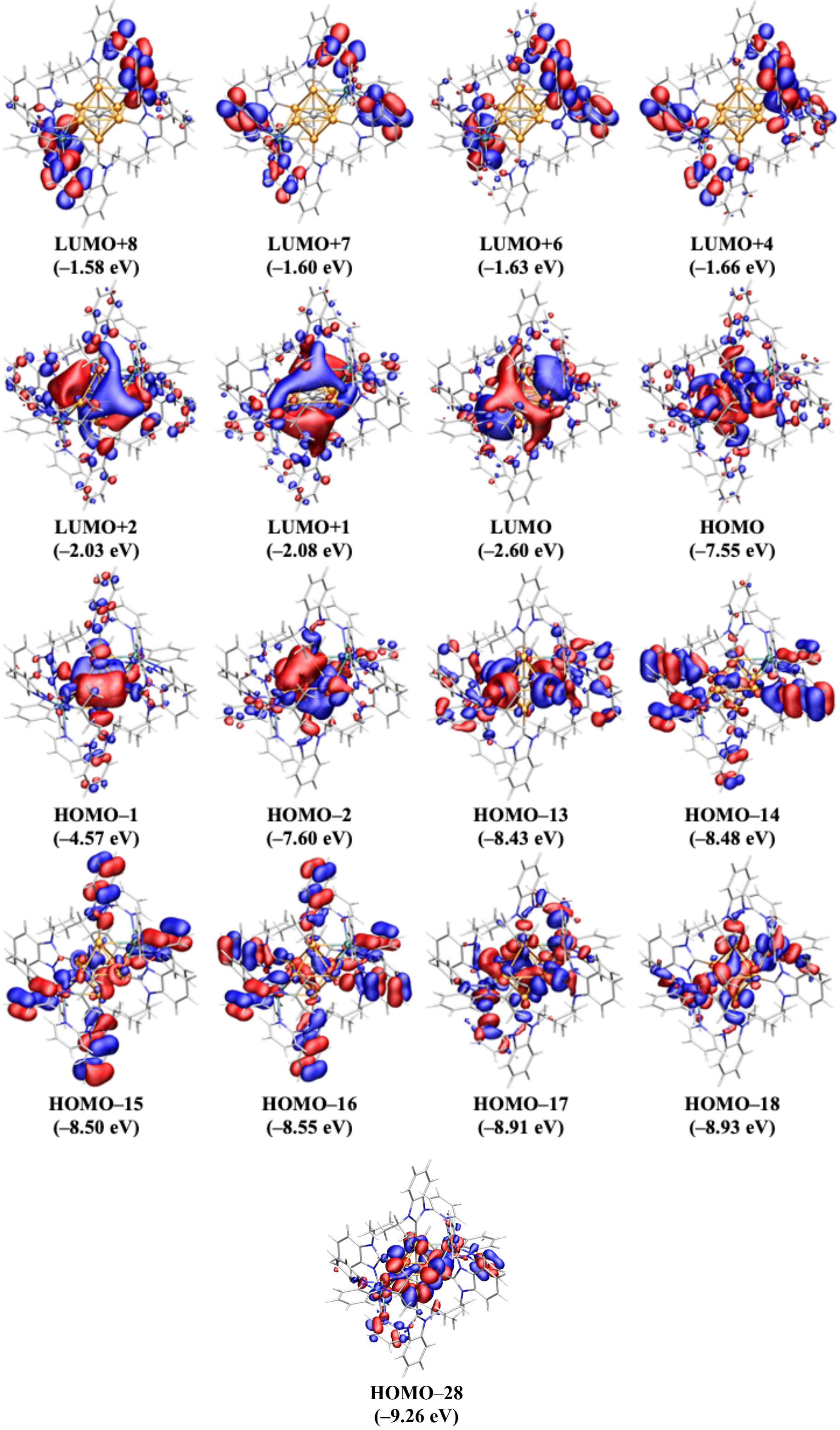

Figure S44. Selected molecular orbitals of **3** (isovalue = 0.02). The HOMO-LUMO gap is 4.96 eV. The values in the parenthesis are the energy levels of the molecular orbitals. Color code: Au orange; Cu cyan; C gray; N blue.

Table S15. Orbital composition analysis with Mulliken partition for selected molecular orbitals of **3**.

Orbital	C-centre	Au^{I}_{6}	Ag^{I}_{2}	$(BIPy)_6$
LUMO+8	0.01%	6.21%	0.20%	93.58%
LUMO+7	0.00%	5.90%	0.33%	93.77%
LUMO+6	0.02%	2.91%	1.37%	95.70%
LUMO+4	0.03%	6.80%	–2.03%	95.20%
LUMO+2	0.05%	47.46%	6.01%	46.48%
LUMO+1	0.06%	49.79%	6.02%	44.12%
LUMO	0.15%	49.79%	22.12%	27.93%
HOMO	23.82%	44.81%	2.04%	29.33%
HOMO–1	24.24%	54.20%	1.22%	20.34%
HOMO–2	24.26%	53.73%	1.13%	20.88%
HOMO–13	0.08%	47.28%	8.70%	43.94%
HOMO–14	3.05%	11.31%	0.67%	84.97%
HOMO–15	3.02%	11.16%	0.68%	85.14%
HOMO–16	5.24%	9.55%	–0.07%	85.27%
HOMO–17	3.74%	30.92%	28.35%	36.99%
HOMO–18	3.29%	30.51%	29.49%	36.71%
HOMO–28	0.71%	69.86%	4.51%	24.93%

Table S16. Excited states of **3*** with oscillator strength (*f*) greater than 0.02, calculated using MN15/6-31G*(C, N, H) ~ LANL2DZ (Au, Ag). The values in the parenthesis are coefficients for singly excited configurations.

State number	λ (nm)	ΔE (eV)	*f*	Transition character
1	424	2.923	0.0412	H→L (0.97)
2	424	2.924	0.0409	H–1→L (0.97)
3	382	3.246	0.4788	H–2→L (0.97)
7	307	4.037	0.3086	H–1→L+1 (0.60), H→L+2 (0.51)
8	306	4.051	0.2028	H→L+2 (0.56), H–1→L+1 (–0.51)
9	306	4.051	0.2024	H–1→L+2 (0.58), H→L+1 (0.48)
10	295	4.201	0.0389	H–2→L+1 (0.72)
11	295	4.204	0.0393	H–2→L+2 (0.72), H–6→L (0.48)
12	286	4.331	0.1271	H–5→L (0.66)
13	286	4.333	0.1291	H–6→L (0.65)
14	281	4.405	0.0498	H–17→L (0.55)
15	281	4.406	0.0523	H–18→L (0.56)
17	276	4.488	0.0496	H–1→L+4 (0.39), H→L+5 (–0.38)
18	276	4.489	0.0478	H→L+4 (0.40), H–1→L+5 (0.37)
19	274	4.530	0.0288	H–1→L+5 (0.41), H→L+4 (–0.39)
21	273	4.547	0.0444	H–16→L (0.62), H–7→L (0.50)

22	272	4.565	0.0461	H–9→L (0.63), H–14→L (0.50)
23	272	4.566	0.0395	H–10→L (0.65), H–15→L (–0.48)
24	271	4.570	0.1594	H–7→L (0.72), H–16→L (0.48)
25	271	4.572	0.0405	H–11→L (0.86)
30	268	4.631	0.0435	H–1→L+3 (0.51)
31	268	4.631	0.0437	H→L+3 (0.53)
35	265	4.682	0.1602	H–4→L+2 (0.46), H–3→L+1 (0.43)
38	264	4.698	0.0615	H→L+6 (0.64), H–1→L+6 (–0.40)
39	264	4.698	0.0599	H–1→L+6 (0.65), H→L+6 (0.40)
42	260	4.760	0.0645	H–2→L+6 (0.53)
45	257	4.823	0.0796	H–1→L+7 (0.43), H→L+7 (0.42)
46	257	4.824	0.0798	H→L+7 (0.42), H→L+8 (–0.40)
53	255	4.867	0.0293	H–1→L+9 (0.41), H→L+10 (0.40)
54	255	4.867	0.0288	H→L+9 (0.41), H–1→L+10 (0.39)
55	254	4.881	0.1352	H–1→L+10 (0.36), H→L+9 (–0.33)
57	252	4.915	0.0921	H–2→L+9 (0.39)
58	252	4.916	0.0921	H–2→L+10 (0.39)
61	252	4.925	0.4889	H–21→L (0.35), H–2→L+6 (–0.35)
62	251	4.949	0.0682	H–2→L+7 (0.33), H–1→L+23 (0.31)
63	250	4.950	0.0680	H–2→L+8 (0.34), H→L+23 (–0.30)
67	248	4.999	0.0779	H–28→L (0.36)
68	248	4.999	0.0989	H–29→L (0.32)
69	248	5.008	0.1868	H–2→L+6 (0.51)
70	248	5.008	0.2619	H–28→L (0.43)
71	248	5.009	0.2527	H–29→L (0.43)
72	246	5.038	0.1848	H–2→L+7 (0.28), H–2→L+10 (0.28)
73	246	5.039	0.1786	H–2→L+8 (0.29), H–4→L+6 (–0.28)
75	245	5.061	0.0462	H–2→L+4 (0.32), H–2→L+8 (0.32)
76	245	5.062	0.0419	H–2→L+7 (0.32), H–2→L+5 (0.32)
77	245	5.063	0.0793	H–21→L (0.25), H–9→L+2 (0.24)
78	243	5.093	0.0381	H–2→L+10 (0.19), H→L+12 (–0.19)
79	243	5.093	0.0377	H–2→L+9 (0.19), H–1→L+12 (0.18)
80	243	5.102	0.0596	H→L+12 (0.32), H–1→L+13 (–0.31), H–31→L (0.31), H–21→L (0.31)
81	243	5.103	0.0243	H–1→L+12 (0.32), H→L+13 (0.32)
82	242	5.114	0.0629	H→L+12 (0.35), H–1→L+13 (0.31)
83	242	5.114	0.0608	H–1→L+12 (0.34), H→L+13 (–0.34)
84	242	5.123	0.0477	H→L+12 (0.41), H–1→L+13 (0.35)
85	242	5.124	0.0496	H–1→L+12 (0.37), H→L+13 (–0.37)
87	241	5.136	0.1513	H→L+13 (0.39), H–31→L (0.38), H–1→L+12 (0.38)
90	239	5.188	0.0947	H–35→L (0.35)
91	239	5.189	0.0919	H–36→L (0.36)
96	237	5.239	0.0352	H–2→L+12 (0.47)
97	237	5.241	0.0344	H–2→L+13 (0.46)

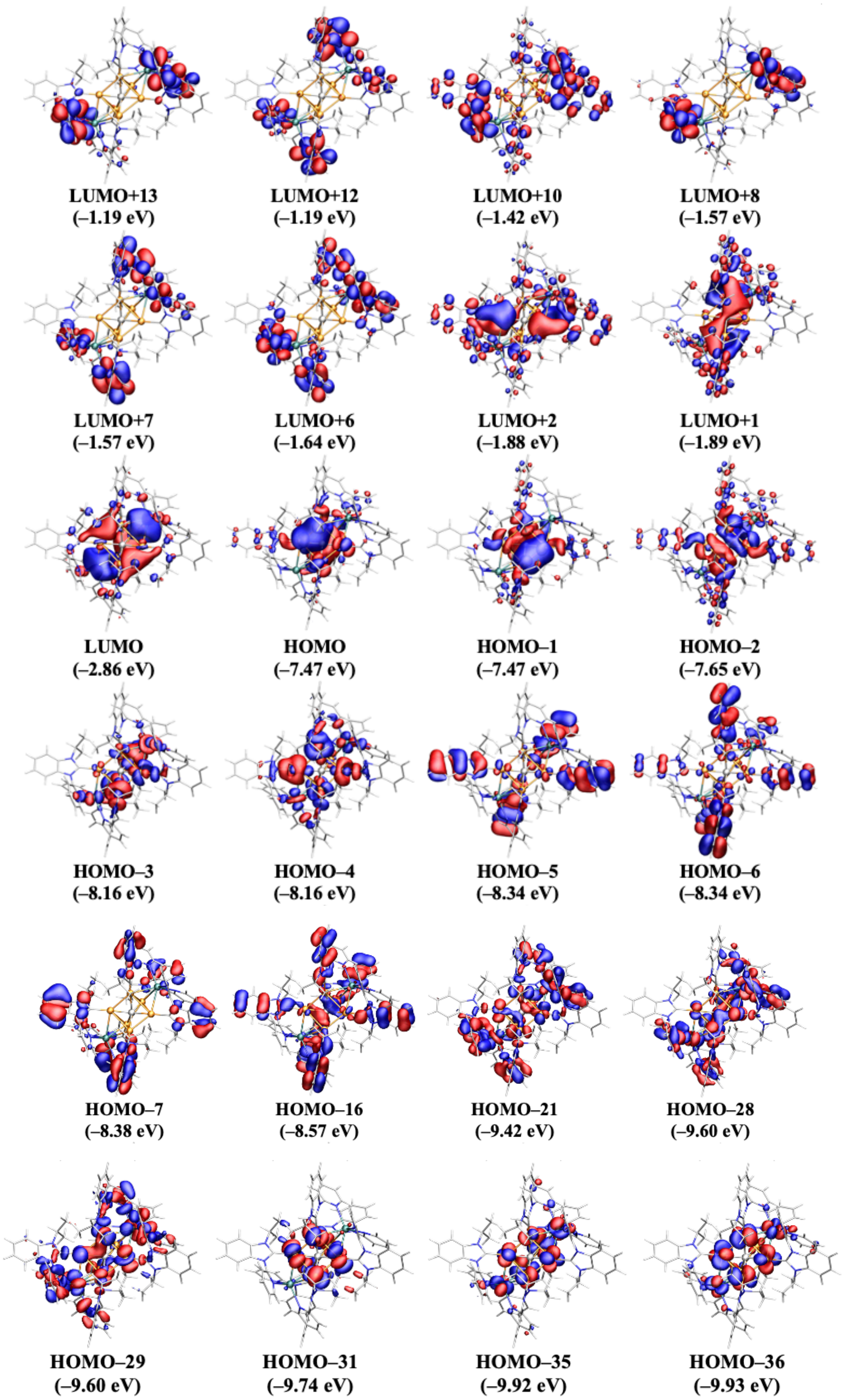

Figure S45. Selected molecular orbitals of **3*** (isovalue = 0.02). The HOMO-LUMO gap is 4.61 eV. The values in the parenthesis are the energy levels of the molecular orbitals. Color code: Au orange; Cu cyan; C gray; N blue.

Table S17. Orbital composition analysis with Mulliken partition for selected molecular orbitals of **3***.

Orbital	C-centre	Au^{I}_{6}	Ag^{I}_{2}	$(BIPy)_6$
LUMO+13	0.18%	8.16%	1.14%	90.52%
LUMO+12	0.18%	8.15%	1.14%	90.53%
LUMO+10	0.44%	15.82%	–1.40%	85.14%
LUMO+8	0.03%	10.89%	–0.74%	89.81%
LUMO+7	0.03%	10.81%	–0.71%	89.87%
LUMO+6	0.03%	3.99%	–0.52%	96.50%
LUMO+2	0.15%	35.73%	5.60%	58.52%
LUMO+1	0.14%	35.87%	5.59%	58.40%
LUMO	0.15%	50.89%	22.21%	26.75%
HOMO	25.91%	54.21%	0.13%	19.75%
HOMO–1	25.92%	54.20%	0.13%	19.75%
HOMO–2	21.10%	42.41%	3.38%	33.11%
HOMO–3	0.15%	61.28%	12.49%	26.07%
HOMO–4	0.15%	61.24%	12.47%	26.14%
HOMO–5	0.13%	9.87%	0.74%	89.25%
HOMO–6	0.14%	9.90%	0.76%	89.20%
HOMO–7	0.02%	0.32%	0.08%	99.58%
HOMO–16	6.78%	11.18%	0.15%	81.89%
HOMO–21	1.43%	24.99%	8.63%	64.94%
HOMO–28	1.19%	27.10%	16.03%	55.67%
HOMO–29	1.12%	30.17%	15.65%	53.06%
HOMO–31	0.00%	87.50%	0.60%	11.90%
HOMO–35	0.05%	71.33%	17.37%	11.26%
HOMO–36	0.05%	72.46%	16.84%	10.65%

Table S18. Excited states of **4** with oscillator strength (*f*) greater than 0.02, calculated using MN15/6-31G*(C, P, N, H) ~ LANL2DZ (Au, Ag). The values in the parenthesis are coefficients for singly excited configurations.

State number	λ (nm)	ΔE (eV)	*f*	Transition character
3	389	3.185	0.1776	H→L (0.98)
9	315	3.940	0.0973	H–1→L+1 (0.69), H–2→L+2 (–0.62)
10	313	3.964	0.0738	H–2→L+2 (0.60), H–1→L+1 (0.55)
11	313	3.966	0.0736	H–1→L+2 (0.58), H–2→L+1 (–0.57)
12	293	4.237	0.0762	H–6→L (0.55), H–5→L (0.48)
13	293	4.239	0.0767	H–5→L (0.55), H–6→L (–0.48)
17	275	4.510	0.0870	H–6→L (0.40), H–21→L (0.39), H–5→L (–0.38)

18	275	4.512	0.0874	H–5→L (0.40), H–22→L (0.39), H–6→L (0.38)
19	275	4.514	0.1699	H→L+3 (0.75)
22	272	4.559	0.1276	H–1→L+3 (0.75)
23	272	4.560	0.1284	H–2→L+3 (0.75)
25	270	4.591	0.4208	H–13→L (0.77)
33	262	4.724	0.0364	H→L+5 (0.42), H→L+6 (–0.41)
34	262	4.725	0.0356	H→L+6 (0.42), H→L+5 (0.41)
35	261	4.753	0.0697	H–1→L+5 (0.33), H–2→L+6 (–0.32)
43	256	4.850	0.1142	H–20→L (0.34), H–6→L+1 (0.32), H–5→L+2 (–0.32)
46	255	4.862	0.0335	H–18→L (0.61)
47	255	4.863	0.0344	H–19→L (0.64)
54	254	4.890	0.0922	H–4→L+4 (0.31), H–1→L+6 (–0.29)
55	253	4.891	0.0913	H–2→L+6 (0.31), H–3→L+4 (–0.30)
56	253	4.900	0.0814	H–1→L+6 (0.46), H–2→L+5 (0.45)
58	252	4.921	0.0331	H–21→L (0.49), H–29→L (–0.47)
59	252	4.922	0.0331	H–22→L (0.49), H–30→L (0.47)
62	250	4.965	0.1126	H–5→L+2 (0.35), H–6→L+1 (–0.34)
66	248	5.009	0.3321	H–5→L+1 (0.39), H–6→L+1 (0.37)
67	247	5.010	0.3325	H–5→L+2 (0.38), H–6→L+1 (0.38)
69	247	5.029	0.0509	H–26→L (0.90)
72	245	5.057	0.0878	H–6→L+2 (0.51), H–5→L+1 (0.50)
75	243	5.095	0.1445	H–6→L+2 (0.24), H–5→L+1 (–0.23)
76	243	5.097	0.1469	H–5→L+2 (0.25), H–4→L+4 (0.23)
77	243	5.112	0.1999	H→L+11 (0.40)
81	240	5.157	0.0483	H–39→L (0.76)
83	239	5.188	0.1826	H→L+15 (0.34)
84	239	5.189	0.1703	H→L+14 (0.32)
88	238	5.201	0.0633	H–41→L (0.43)
89	238	5.201	0.0628	H–42→L (0.43)
90	238	5.214	0.0255	H→L+11 (0.36)
91	237	5.224	0.1377	H–8→L+1 (0.27), H–35→L (0.27)
92	237	5.225	0.1316	H–8→L+2 (0.26), H–34→L (0.26)
96	237	5.242	0.1278	H–39→L (–0.27), H→L+11 (0.25)
97	236	5.246	0.0526	H–34→L (0.43)
98	236	5.247	0.0612	H–35→L (0.39)
99	236	5.252	0.0467	H–34→L (0.45)
100	236	5.253	0.0352	H–35→L (0.50)

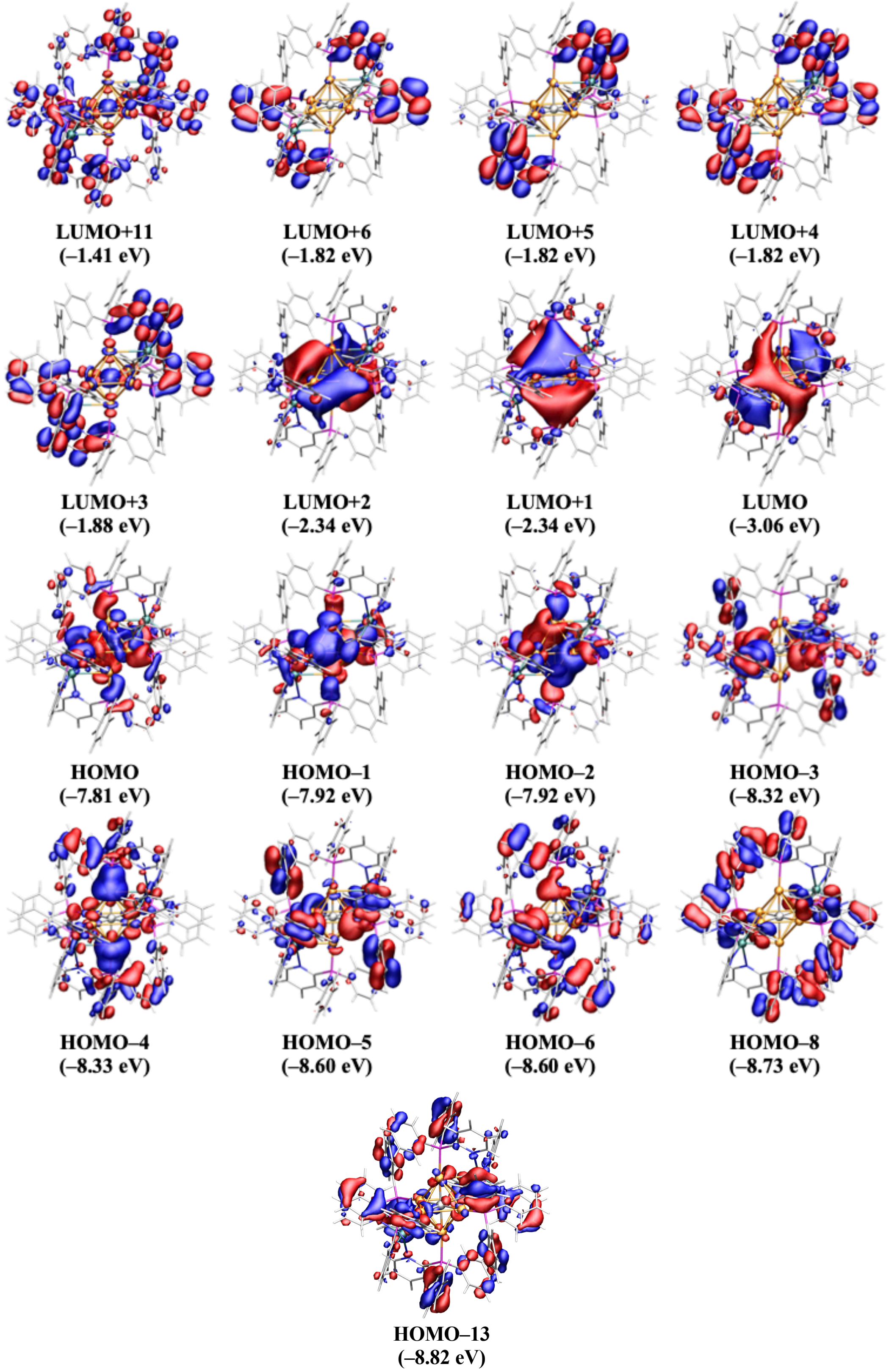

Figure S46. Selected molecular orbitals of **4** (isovalue = 0.02). The HOMO-LUMO gap is 4.75 eV. The values in the parenthesis are the energy levels of the molecular orbitals. Color code: Au orange; Ag grass green; C gray; N blue.

Table S19. Orbital composition analysis with Mulliken partition for selected molecular orbitals of **4**.

Orbital	C-centre	Au^{I}_{6}	Ag^{I}_{2}	$(dppy)_6$
LUMO+11	1.83%	8.67%	0.23%	89.28%
LUMO+6	0.03%	9.33%	3.00%	87.64%
LUMO+5	0.03%	9.26%	3.03%	87.68%
LUMO+4	1.53%	0.66%	4.00%	93.81%
LUMO+3	2.73%	11.74%	1.05%	84.48%
LUMO+2	0.06%	69.84%	–0.39%	30.48%
LUMO+1	0.06%	69.87%	–0.32%	30.39%
LUMO	0.07%	56.93%	18.48%	24.52%
HOMO	26.83%	32.58%	2.23%	38.36%
HOMO–1	28.50%	43.24%	1.08%	27.18%
HOMO–2	28.49%	43.20%	1.07%	27.24%
HOMO–3	0.03%	33.78%	3.96%	62.23%
HOMO–4	0.03%	33.70%	3.98%	62.29%
HOMO–5	–0.24%	23.44%	2.49%	74.31%
HOMO–6	–0.24%	23.26%	2.49%	74.49%
HOMO–8	0.08%	1.53%	0.43%	97.96%
HOMO–13	1.00%	18.02%	0.16%	80.83%

Table S20. Excited states of **4*** with oscillator strength (*f*) greater than 0.02, calculated using MN15/6-31G*(C, P, N, H) ~ LANL2DZ (Au, Ag). The values in the parenthesis are coefficients for singly excited configurations.

State number	λ (nm)	Δ*E* (eV)	*f*	Transition character
3	410	3.025	0.1951	H–2→L (0.97)
6	307	4.038	0.0255	H–1→L+1 (0.59), H→L+2 (–0.53)
12	297	4.176	0.0216	H–30→L (0.41)
13	297	4.177	0.0225	H–29→L (0.41)
16	292	4.244	0.0926	H–5→L (0.92)
17	292	4.253	0.1166	H–8→L (0.62)
18	291	4.253	0.1167	H–9→L (0.64)
25	277	4.472	0.0395	H–13→L (0.75)
30	273	4.536	0.0341	H–26→L (0.37), H–15→L (–0.37)
31	273	4.537	0.0339	H–27→L (0.37), H–14→L (0.37)
34	272	4.553	0.0491	H→L+3 (0.56)
35	272	4.554	0.0530	H–1→L+3 (0.59)
43	268	4.626	0.6088	H–2→L+3 (0.37)
46	265	4.686	0.0394	H–23→L (0.43)
47	265	4.687	0.0444	H–24→L (0.42)
48	264	4.689	0.5524	H–3→L+2 (0.42), H–2→L+3 (0.42)
49	264	4.700	0.0826	H–25→L (0.53)

50	263	4.705	0.0433	H–27→L (0.39)
51	263	4.706	0.0359	H–26→L (0.40)
52	263	4.709	0.1207	H–2→L+3 (0.52)
60	259	4.795	0.0620	H–25→L (0.52)
62	257	4.821	0.0570	H→L+8 (0.24)
63	257	4.822	0.0587	H–3→L+5 (0.22), H–2→L+6 (–0.22), H–45→L (–0.22)
65	254	4.873	0.0416	H–39→L (0.50)
66	254	4.885	0.0577	H–46→L (0.59)
69	252	4.918	0.0507	H–2→L+18 (0.29), H–4→L+6 (0.28)
70	252	4.925	0.1287	H–41→L (0.24), H–4→L+4 (–0.23)
71	252	4.926	0.1287	H–42→L (0.24)
76	248	4.990	0.0550	H–1→L+7 (0.44), H→L+8 (–0.40)
77	248	4.990	0.0553	H–1→L+8 (0.42), H→L+7 (0.42)
79	246	5.031	0.0453	H–33→L (0.36)
82	245	5.070	0.1970	H–2→L+8 (0.31)
83	245	5.071	0.1970	H–2→L+7 (0.32)
93	241	5.151	0.0862	H–4→L+10 (0.26), H–3→L+9 (0.26)
94	241	5.152	0.0837	H–3→L+10 (0.26)
96	240	5.175	0.0212	H→L+18 (0.52)
97	240	5.176	0.0237	H–1→L+18 (0.52)
98	239	5.181	0.0387	H–5→L+2 (0.21), H–1→L+10 (–0.20)
99	239	5.181	0.0358	H–1→L+9 (0.22), H→L+10 (–0.21)
100	239	5.196	0.2051	H→L+15 (0.29), H–1→L+16 (0.28)

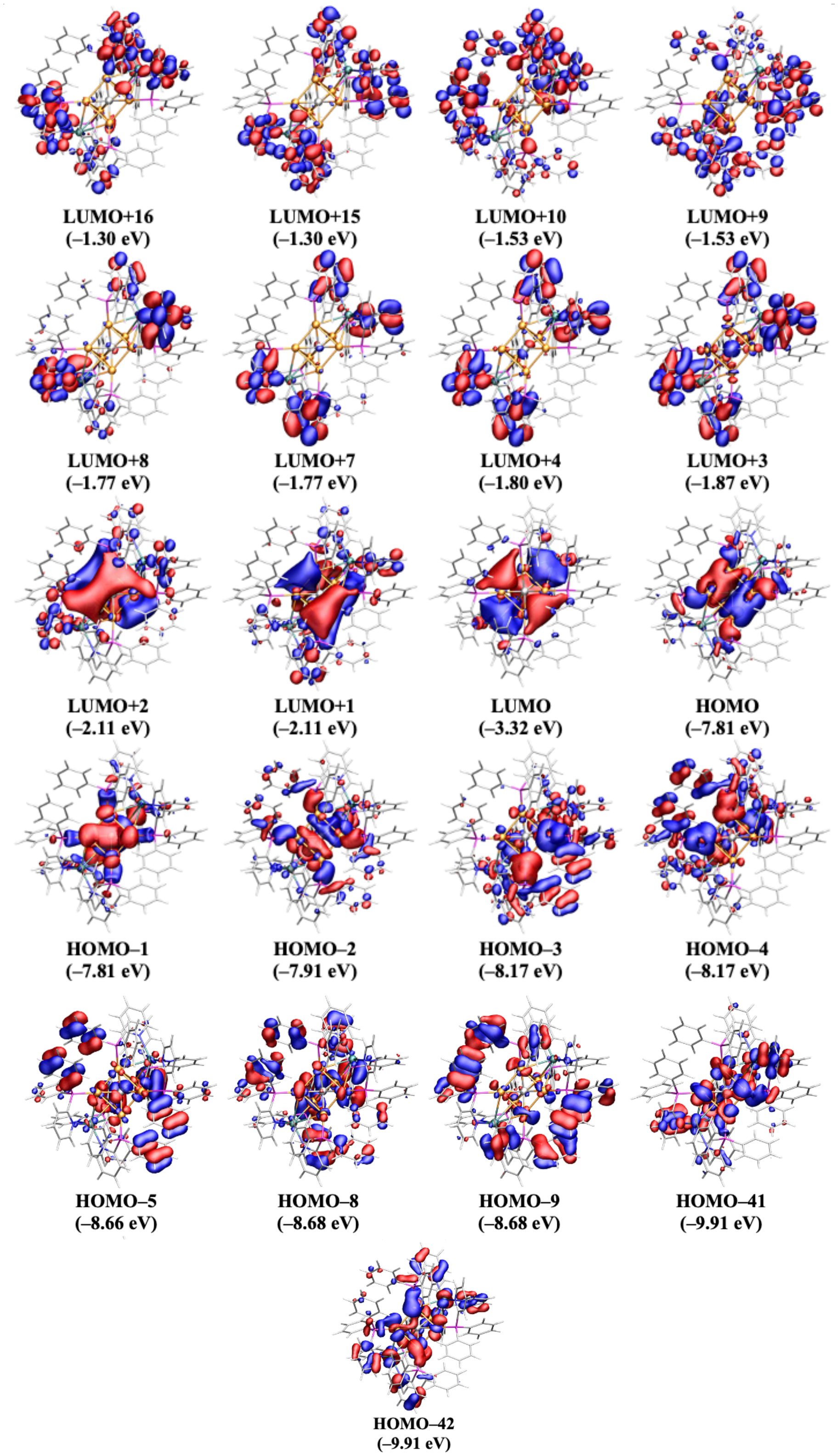

Figure S47. Selected molecular orbitals of **4*** (isovalue = 0.02). The HOMO-LUMO gap is 4.50 eV. The values in the parenthesis are the energy levels of the molecular orbitals. Color code: Au orange; Cu cyan; C gray; N blue.

Table S21. Orbital composition analysis with Mulliken partition for selected molecular orbitals of **4***.

Orbital	C-centre	Au^{I}_6	Ag^{I}_2	$(dppy)_6$
LUMO+16	0.12%	12.42%	2.26%	85.20%
LUMO+15	0.12%	12.38%	2.24%	85.25%
LUMO+10	0.06%	11.60%	1.91%	86.43%
LUMO+9	0.06%	11.69%	1.90%	86.35%
LUMO+8	0.56%	5.29%	2.88%	91.27%
LUMO+7	0.59%	4.86%	2.73%	91.83%
LUMO+4	1.33%	5.20%	2.27%	91.20%
LUMO+3	2.04%	9.24%	0.36%	88.36%
LUMO+2	0.12%	51.05%	–0.12%	48.95%
LUMO+1	0.11%	51.02%	–0.12%	48.98%
LUMO	0.01%	56.92%	17.78%	25.29%
HOMO	28.80%	44.72%	0.57%	25.92%
HOMO–1	28.80%	44.74%	0.57%	25.89%
HOMO–2	26.10%	32.50%	1.41%	39.99%
HOMO–3	0.17%	36.68%	5.22%	57.93%
HOMO–4	0.16%	36.58%	5.21%	58.04%
HOMO–5	2.82%	9.39%	3.84%	83.96%
HOMO–8	0.44%	13.66%	0.87%	85.02%
HOMO–9	0.45%	13.75%	0.59%	85.20%
HOMO–41	0.60%	47.38%	11.02%	41.00%
HOMO–42	0.57%	48.11%	11.15%	40.16%

8. References

1. Lei, Z.; Endo, M.; Ube, H.; Shiraogawa, T.; Zhao, P.; Nagata, K.; Pei, X.-L.; Eguchi, T.; Kamachi, T.; Ehara, M.; Ozawa, T.; Shionoya, M. *N*-Heterocyclic carbene-based C-centered Au(I)-Ag(I) clusters with intense phosphorescence and organelle-selective translocation in cells. *Nat. Commun.* **2022**, *13*, 4288.
2. Jia, J.-H.; Wang, Q.-M. Intensely luminescent gold(I)-silver(I) cluster with hypercoordinated carbon. *J. Am. Chem. Soc.* **2009**, *131*, 16634–16635.
3. Jia, J.-H.; Liang, J.-X.; Lei, Z.; Cao, Z.-X.; Wang, Q.-M. A luminescent gold(I)-copper(I) cluster with unprecedented carbon-centered trigonal prismatic hexagold. *Chem. Commun.* **2011**, *47*, 4739–4741.
4. Asakura, H.; Yamazoe, S.; Misumi, T.; Fujita, A.; Tsukuda, T.; Tanaka, T. xTunes: A new XAS processing tool for detailed and on-the-fly analysis. *Radiat. Phys. Chem.* **2020**, *175*, 108270–108273.
5. Ankudinov, A. L.; Ravel, B.; Rehr, J. J.; Conradson, S. D. Real-space multiple-scattering calculation and interpretation of x-ray-absorption near-edge structure. *Phys. Rev. B* **1998**, *58*, 7565–7576.

6. Becke, A. D. Density-functional thermochemistry. III. The role of exact exchange. *J. Chem. Phys.* **1993**, *98*, 5648–5652.
7. Hay, P. J.; Wadt, W. R. Ab initio effective core potentials for molecular calculations. Potentials for K to Au including the outermost core orbitals. *J. Chem. Phys.* **1985**, *82*, 299–310.
8. Ditchfield, R.; Hehre, W. J.; Pople, J. A. Self-consistent molecular-orbital methods. IX. An extended Gaussian-type basis for molecular-orbital studies of organic molecules. *The J. Chem. Phys.* **1971**, *54*, 724–728.
9. Francl, M. M. *et al.* Self-consistent molecular orbital methods. XXIII. A polarization-type basis set for second-row elements. *J. Chem. Phys.* **1982**, *77*, 3654–3665.
10. Gordon, M. S.; Binkley, J. S.; Pople, J. A.; Pietro, W. J.; Hehre, W. J. Self-consistent molecular-orbital methods. 22. Small split-valence basis sets for second-row elements. *J. Am. Chem. Soc.* **1982**, *104*, 2797–2803.
11. Hariharan, P. C.; Pople, J. A. The influence of polarization functions on molecular orbital hydrogenation energies. *Theoretica Chimica Acta* **1973**, *28*, 213–222.
12. Hehre, W. J.; Ditchfield, R.; Pople, J. A. Self-consistent molecular orbital methods. XII. Further extensions of Gaussian—type basis sets for use in molecular orbital studies of organic molecules. *J. Chem. Phys.* **1972**, *56*, 2257–2261.
13. Yu, H. S.; He, X.; Li, S. L.; Truhlar, D. G. MN15: A Kohn–Sham global-hybrid exchange–correlation density functional with broad accuracy for multi-reference and single-reference systems and noncovalent interactions. *Chem. Sci.* **2016**, *7*, 5032–5051.
14. Tomasi, J.; Mennucci, B.; Cammi, R. Quantum mechanical continuum solvation models. *Chem. Rev.* **2005**, *105*, 2999–3094.
15. O'Boyle, N. M.; Tenderholt, A. L.; Langner, K. M. Cclib: A library for package-independent computational chemistry algorithms. *J. Comput. Chem.* **2008**, *29*, 839–845.
16. Chai, J.-D.; Head-Gordon, M. Long-range corrected hybrid density functionals with damped atom–atom dispersion corrections. *Phys. Chem. Chem. Phys.* **2008**, *10*, 6615–6620.
17. Zhao, Y.; Truhlar, D. G. The M06 suite of density functionals for main group thermochemistry, thermochemical kinetics, noncovalent interactions, excited states, and transition elements: two new functionals and systematic testing of four M06-class functionals and 12 other functionals. *Theo. Chem. Acc.* **2008**, *120*, 215–241.
18. Glendening, E. D.; Reed, A. E.; Carpenter, J. E.; Weinhold, F. NBO Version 3.1.
19. Frisch, M. J. *et al.* Gaussian 16 Rev. A.03. 2016.
